# Comparative effectiveness of second line oral antidiabetic treatments among people with type 2 diabetes mellitus: emulation of a target trial using routinely collected health data

**DOI:** 10.1136/bmj-2023-077097

**Published:** 2024-05-08

**Authors:** Patrick Bidulka, David G Lugo-Palacios, Orlagh Carroll, Stephen O’Neill, Amanda I Adler, Anirban Basu, Richard J Silverwood, Jonathan W Bartlett, Dorothea Nitsch, Paul Charlton, Andrew H Briggs, Liam Smeeth, Ian J Douglas, Kamlesh Khunti, Richard Grieve

**Affiliations:** 1 Department of Non-Communicable Disease Epidemiology, London School of Hygiene and Tropical Medicine, London, WC1E 7HT, UK; 2 Department of Health Services Research and Policy, London School of Hygiene and Tropical Medicine, London, UK; 3 Diabetes Trials Unit, The Oxford Centre for Diabetes, Endocrinology and Metabolism, University of Oxford, Headington, Oxford, UK; 4 The Comparative Health Outcomes, Policy and Economics (CHOICE) Institute, University of Washington School of Pharmacy, Seattle, WA, USA; 5 Centre for Longitudinal Studies, UCL Social Research Institute, University College London, London, UK; 6 Department of Medical Statistics, London School of Hygiene and Tropical Medicine, London, UK; 7 Patient author, Patient Research Champion Team, National Institute for Health and Care Research, London, UK; 8 Diabetes Research Centre, University of Leicester, Leicester, UK

## Abstract

**Objective:**

To compare the effectiveness of three commonly prescribed oral antidiabetic drugs added to metformin for people with type 2 diabetes mellitus requiring second line treatment in routine clinical practice.

**Design:**

Cohort study emulating a comparative effectiveness trial (target trial).

**Setting:**

Linked primary care, hospital, and death data in England, 2015-21.

**Participants:**

75 739 adults with type 2 diabetes mellitus who initiated second line oral antidiabetic treatment with a sulfonylurea, DPP-4 inhibitor, or SGLT-2 inhibitor added to metformin.

**Main outcome measures:**

Primary outcome was absolute change in glycated haemoglobin A_1c_ (HbA_1c_) between baseline and one year follow-up. Secondary outcomes were change in body mass index (BMI), systolic blood pressure, and estimated glomerular filtration rate (eGFR) at one year and two years, change in HbA_1c_ at two years, and time to ≥40% decline in eGFR, major adverse kidney event, hospital admission for heart failure, major adverse cardiovascular event (MACE), and all cause mortality. Instrumental variable analysis was used to reduce the risk of confounding due to unobserved baseline measures.

**Results:**

75 739 people initiated second line oral antidiabetic treatment with sulfonylureas (n=25 693, 33.9%), DPP-4 inhibitors (n=34 464 ,45.5%), or SGLT-2 inhibitors (n=15 582, 20.6%). SGLT-2 inhibitors were more effective than DPP-4 inhibitors or sulfonylureas in reducing mean HbA_1c_ values between baseline and one year. After the instrumental variable analysis, the mean differences in HbA_1c_ change between baseline and one year were −2.5 mmol/mol (95% confidence interval (CI) −3.7 to −1.3) for SGLT-2 inhibitors versus sulfonylureas and −3.2 mmol/mol (−4.6 to −1.8) for SGLT-2 inhibitors versus DPP-4 inhibitors. SGLT-2 inhibitors were more effective than sulfonylureas or DPP-4 inhibitors in reducing BMI and systolic blood pressure. For some secondary endpoints, evidence for SGLT-2 inhibitors being more effective was lacking—the hazard ratio for MACE, for example, was 0.99 (95% CI 0.61 to 1.62) versus sulfonylureas and 0.91 (0.51 to 1.63) versus DPP-4 inhibitors. SGLT-2 inhibitors had reduced hazards of hospital admission for heart failure compared with DPP-4 inhibitors (0.32, 0.12 to 0.90) and sulfonylureas (0.46, 0.20 to 1.05). The hazard ratio for a ≥40% decline in eGFR indicated a protective effect versus sulfonylureas (0.42, 0.22 to 0.82), with high uncertainty in the estimated hazard ratio versus DPP-4 inhibitors (0.64, 0.29 to 1.43).

**Conclusions:**

This emulation study of a target trial found that SGLT-2 inhibitors were more effective than sulfonylureas or DPP-4 inhibitors in lowering mean HbA_1c_, BMI, and systolic blood pressure and in reducing the hazards of hospital admission for heart failure (*v* DPP-4 inhibitors) and kidney disease progression (*v* sulfonylureas), with no evidence of differences in other clinical endpoints.

## Introduction

About 463 million people worldwide (9.3%) have type 2 diabetes mellitus.^1^ In most people this disease is progressive, and it is associated with risks of multiple complications, including cardiovascular disease (CVD) and chronic kidney disease.^2^ Interventions that improve biomarkers of type 2 diabetes mellitus, such as glycated haemoglobin A_1c_ (HbA_1c_), blood pressure, and lipid levels, can reduce the risk of these complications.^3^ ^4^ ^5^ ^6^ International clinical guidelines recommend additional drugs (second line treatment) if glycaemic control is inadequate after metformin monotherapy.^7^ ^8^ ^9^ A recent study of second line treatments for people with type 2 diabetes mellitus across 38 countries reported that the most commonly used oral drugs were dipeptidyl peptidase-4 (DPP-4) inhibitors (48.3%), sulfonylureas (40.9%), and sodium-glucose cotransporter-2 (SGLT-2) inhibitors (8.3%).^10^

Of these oral treatments, SGLT-2 inhibitors are newer and more costly classes of drugs.^11^ In England, SGLT-2 inhibitors are recommended second line treatments in preference to other drug classes for some people with type 2 diabetes mellitus—those with pre-existing CVD, at high risk of CVD, or with kidney disease.^7^ For most people with type 2 diabetes mellitus, however, evidence on the comparative effectiveness of these alternative drugs classes, particularly in relation to reducing HbA_1c_ levels, is insufficient to recommend a particular second line treatment.^7^ An international consensus statement^9^ and guidelines from the National Institute of Health and Care Excellence (NICE)^7^ therefore leaves the choice of second line treatment for most people with type 2 diabetes mellitus to clinicians and patients, which has led to wide variation across groups of primary care providers in England in the proportion of people prescribed each drug class.^12^ Current NICE (2022) guidelines recommend other antidiabetic treatments, such as insulin based therapy and glucagon-like peptide-1 receptor agonists, only if HbA_1c_ levels are not controlled after second line treatment with oral antidiabetics.^7^ Hence in many countries, including England, the proportion of people with type 2 diabetes mellitus who are prescribed glucagon-like peptide-1 receptor agonists as second line treatment is low.^10^ ^12^ ^13^

Most randomised controlled trials assessing the effectiveness and safety of SGLT-2 inhibitors and DPP-4 inhibitors have randomised groups to an active intervention or placebo comparator.^14^ ^15^ ^16^ ^17^ ^18^ ^19^ ^20^ ^21^ ^22^ ^23^ ^24^ ^25^ ^26^ Therefore, although these trials reported fewer CVD and kidney events in people with and without type 2 diabetes mellitus allocated to SGLT-2 inhibitors, the results are difficult to apply to routine clinical practice, where the relevant populations and comparators differ.^16^ ^17^ ^18^ ^19^ ^20^ ^21^ ^22^ ^23^ ^24^ Of the randomised controlled trials with an active comparator, some compared DPP-4 inhibitors with sulfonylureas^27^ ^28^ ^29^ ^30^ or compared SGLT-2 inhibitors with sulfonylureas,^31^ but none compared all three drug classes. Thus the comparative effectiveness of SGLT-2 inhibitors versus alternative second line oral antidiabetic treatments on outcomes important to people with type 2 diabetes mellitus, particularly reduction in HbA_1c_ level, remains unclear. Results from previous observational studies comparing these treatments^32^ ^33^ ^34^ are at risk of bias from residual (unmeasured) confounding. Although a recent observational study^35^ emulated some of the results of the GRADE (Glycemia Reduction Approaches In Diabetes: A Comparative Effectiveness Study) randomised trial,^29^ ^36^ ^37^ neither the trial nor the observational study considered SGLT-2 inhibitors, which limits the applicability of the results to routine clinical practice.

Recent advances in real world data combined with developments in quantitative methods offer important opportunities for generating evidence on comparative effectiveness of treatments with direct relevance to clinical practice.^35^ In this study, we illustrated the potential and challenges of using real world data from Clinical Practice Research Datalink (CPRD) for these purposes. We emulated the design of a hypothetical pragmatic randomised controlled trial by comparing three antidiabetic drug classes (sulfonylureas, DPP-4 inhibitors, and SGLT-2 inhibitors) of interest to the broad population of people with type 2 diabetes mellitus who, according to current NICE guidelines, are eligible for any of these second line treatments. We considered intermediate metabolic outcomes, particularly HbA_1c_ level, but also kidney and cardiovascular related complications. To reduce the risk of unmeasured confounding we used prescriber variation as an instrumental variable to estimate treatment effectiveness from routine data.^38^ ^39^ Our study complements a recent target trial emulation that assessed the comparative effectiveness of alternative second line treatments using data from the Department of United States Veterans Affairs,^40^ but which underrepresented female members of the population (<10%) and in the main analyses assumed that that there was no unmeasured confounding.

We compared the effectiveness of the three most prescribed second line antidiabetic treatments in the UK according to metabolic and other clinical measures (changes from baseline in HbA_1c_ level, estimated glomerular filtration rate (eGFR), body mass index (BMI), and systolic blood pressure) and to adverse clinical endpoints (kidney and cardiovascular outcomes, and death).

## Methods

### Study design

We designed this study according to the target trial framework.^41^ Briefly, a target trial is a hypothetical randomised controlled trial for assessing comparative effectiveness from observational data that requires pre-specification of the main elements of a trial’s protocol, including eligibility criteria, the respective treatment strategies, time zero, and an analysis plan.^41^ The target trial emulation reported in this paper is part of the PERMIT (PERsonalised Medicine for Intensification of Treatment) study, which prespecified the definition of the eligibility criteria and treatment strategies in the published versions of the study protocol^42^ and other elements of the target trial emulation in the statistical analysis plan.^43^ Supplementary table 1 provides details to accompany this paper of how each of the standpoints were emulated (eligibility criteria, treatment assignment, initiation, and strategy, follow-up, outcomes, causal contrasts of interest, and analysis strategy).

We applied target trial principles to primary care data from CPRD to identify people with type 2 diabetes mellitus who had a similar prognosis before initiating any of the three second line antidiabetic treatments under comparison. CPRD covers about 20% of the UK population registered with general practices and includes longitudinal information on primary care diagnoses, prescriptions, personal information, and laboratory test results.^44^ ^45^ Linkage from CPRD to Hospital Episode Statistics in-patient data was available for about 90% of participating practices in England. We accessed information from Hospital Episodes Statistics admitted patient care database on diagnoses, procedures, sociodemographic characteristics, and admission and discharge dates.^46^ Rather than relying on a single data source to ascertain cardiovascular and kidney outcomes, we used linked data from CPRD-Hospital Episodes Statistics as these have been shown to improve capture of these events and reduce risks of misclassification.^47^ ^48^ Information on each person’s vital status was available through linkage to the Office for National Statistics (ONS) death records.^49^ ^50^

### Study population

We defined the study population according to eligibility criteria, which had to be met before time zero (baseline) and was analogous to the time of randomisation in a randomised controlled trial. Time zero was defined by the date of the first prescription for any of the three oral second line treatments that were added to metformin (see supplementary table 1). We followed precedent research by including people with a diagnosis of type 2 diabetes mellitus who were aged 18 years or older,^33^ ^51^ registered with a general practice in England, and who intensified treatment from first line to second line oral antidiabetic treatment between 1 January 2015 and 31 December 2020 with a first ever prescription of sulfonylureas, DPP-4 inhibitors, or SGLT-2 inhibitors added to metformin. Those eligible had to have at least one prescription for metformin monotherapy within 60 days before the first prescription for second line treatment, to ensure their use of metformin monotherapy was continuous before intensification. We excluded individuals with pregnancy recorded within 12 months before initiation of second line treatment and people whose last recorded eGFR was <30 mL/min/1.73m^2^, since prescribing guidelines recommend different treatments for these groups. We also excluded people whose general practices had not consented to the required linkage of Hospital Episodes Statistics data. We followed precedent research in excluding those who were not prescribed metformin on the same day or within 60 days after initiating second line treatment,^33^ as it is unlikely that their treatment with metformin continued. Supplementary tables 1 and 2 present detailed inclusion and exclusion criteria.

### Treatments under comparison

We compared DPP-4 inhibitors with sulfonylureas and SGLT-2 inhibitors with sulfonylureas and DPP-4 inhibitors as second line oral antidiabetic treatments added to metformin. Information was extracted on the prescribed duration of each treatment and any subsequent antidiabetic treatment.

The study used an intention-to-treat approach so that individuals contributed to the treatment group to which they were assigned at baseline until the end of the follow-up period (see supplementary table 1), irrespective of the extent to which they adhered to the treatment prescribed. We defined the end of follow-up as the earliest of the date the general practice stopped contributing to CPRD, the date the individual left the general practice, the date of death, or the last date of available data (31 December 2021 for continuous outcomes or 31 March 2021 for time-to-event outcomes). We described the duration of second line and third line treatments by comparison group.

### Covariates

We have previously described the covariates in detail,^11^ ^42^ and these are summarised in supplementary table 3. Briefly, we defined patient sociodemographic characteristics (age, sex, ethnicity, index of multiple deprivation), time since diagnosis of type 2 diabetes mellitus, year of initiation of second line antidiabetic treatment, NHS region (East of England, London, Midlands, North East and Yorkshire, North West, South East, and South West),^52^ number of patients registered with the participants’ general practice, smoking and alcohol intake status, relevant co-prescriptions (renin-angiotensin system inhibitors or statins) issued within 60 days before baseline, hospital admission (any) in the previous year, and comorbidities recorded at baseline (history of myocardial infarction, unstable angina, previous stroke, ischaemic heart disease, hypoglycaemia, heart failure, history of any cancer, history of proteinuria, advanced eye disease, lower limb amputation, and impaired kidney function (latest eGFR <60 mL/min/1.73m^2^). We also defined HbA_1c_, systolic blood pressure, diastolic blood pressure, eGFR, and BMI^53^ using the most recent measures recorded in primary care.

For the primary endpoint, change in HbA_1c_ level, we only considered the most recent measure within 180 days before time zero as the baseline measure in line with NICE guidance, which recommends that HbA_1C_ is measured every six months.^7^ For systolic and diastolic blood pressure and eGFR we followed previous research in considering the most recent measure within 540 days before baseline^33^ (see supplementary table 3). We considered any values recorded in advance of these time windows as out-dated, and they were not used to define baseline characteristics. For BMI we followed a previously published algorithm in using the most recent measure available, which for most participants was within six months.^53^

### Outcomes

The primary outcome was the absolute change in HbA_1c_ (mmol/mol) level between baseline and one year after each prescription for second line treatment (HbA_1c_ value at one year–HbA_1c_ value at baseline). Treatment groups were compared according to the mean change in HbA_1c_ level. We used the measurement closest in time to the one year follow-up time point and allowed for measures within ±90 days, otherwise the measure was designated as missing.

Secondary outcomes included change in HbA_1c_ level at two years and change in BMI, systolic blood pressure, and eGFR at one year and two years.^33^ We also reported the time to several first events before two years’ follow-up: a ≥40% decline in eGFR from baseline, which could be a marker for the rarer end stage kidney disease outcome^54^; a major adverse kidney event, a composite outcome for the earliest of a decline in eGFR from baseline of 40%, end stage kidney disease, and all cause mortality^55^; hospital admission for heart failure; major adverse cardiovascular event (MACE), a composite outcome for the earliest of myocardial infarction, stroke, or CVD death; and all cause mortality. We also reported time to myocardial infarction and stroke individually. Time to end stage kidney disease and CVD specific mortality could not be reported owing to the low number of events. Individuals were followed until they experienced the event of interest, died, or linked CPRD-Hospital Episodes Statistics data were no longer available (patient/general practice stopped contributing data to the CPRD or 31 March 2021). For these time-to-event measures, we only considered outcomes within the first two years in the base case, as it was anticipated that at later time points a high proportion of individuals would have censored or missing data. Supplementary table 4 provides details on all outcome definitions, including data sources.

### Statistical analysis

We chose to use an instrumental variable analysis to help reduce the risk of confounding from unobserved baseline measures, such as diet and exercise before initiation of second line treatment (see supplementary methods, supplementary table 1, and supplementary figures 1A and 1B).^38^ The instrumental variable was the primary care providers’ tendency to prescribe the three classes of second line treatment. In England, most primary care clinicians work within a group, and over the study’s timeframe this was defined as a clinical commissioning group (CCG), which informed health funding decisions for its respective geographical region. Some CCGs recommended that a relatively high proportion of people had second line treatment with sulfonylureas or DPP-4 inhibitors due in part to the higher cost of SGLT-2 inhibitors. We therefore defined CCGs rather than individual general practices as the unit for the instrumental variable, as this reflected decision making and was strongly associated with choice of second line treatment.^11^ ^12^

**Fig 1:**
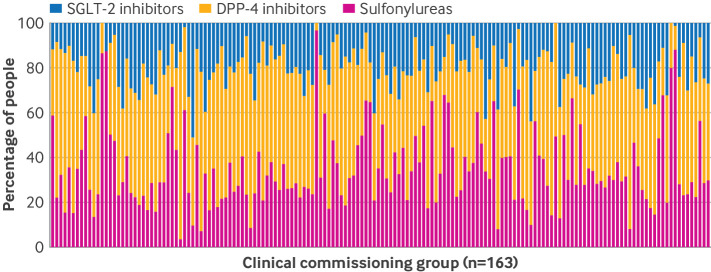
Stacked bar chart illustrating variation in second line antidiabetic treatment prescribed among people included in the study at the clinical commissioning group level in England, 2014-20. DPP-4=dipeptidyl peptidase-4; SGLT-2=sodium-glucose cotransporter-2

We also found wide variation across CCGs in the proportion of people prescribed each of the three classes of second line treatment (fig 1). This natural variation implied that people with a similar prognosis at baseline received a different second line treatment simply according to their CCG. We defined the tendency to prescribe as the proportion of eligible people prescribed each second line treatment within the 12 months preceding the specific baseline (time zero) for each person. A valid instrument must meet four main conditions (see also the direct acyclic graph in supplementary figures 1A and 1B).^38^ Firstly, the instrument must predict the treatment prescribed, which can be formally assessed.^56^ Here, we assessed the relevance of the CCGs tendency to prescribe using a weak instrument test that is robust to heteroscedasticity and clustering by NHS region. Recent work has suggested that to meet the requirement that the instrument is of sufficient strength, the F statistic summarising the association between the instrumental variable and the treatment received must exceed 100.^38^ ^57^ Secondly, the instrument must be independent of covariates that predict the outcomes of interest, which can be partially evaluated. We assessed the extent to which observed prognostic covariates differed across levels of the instrument (see supplementary figures 2A-2C). Thirdly, the instrument must have an effect on the outcomes only through the treatment received, which cannot be evaluated empirically. Large imbalances in measured covariates across levels of the tendency to prescribe would raise concerns about the second and third instrumental variable assumptions. We followed our prespecified protocol^42^ and the statistical analysis plan^43^ and were guided by the direct acyclic graphs (see supplementary figures 1A and 1B) in choosing to adjust for measured contextual and temporal confounders in the second stage (outcome) regression. By including these contextual covariates in the second stage regression we were able to make weaker assumptions, that the tendency to prescribe was independent of the outcome and only had an effect on the outcome through the treatment received after adjusting for any differences in region, general practice size, and time period (see supplementary file). Fourthly, the instrumental variable analysis assumes monotonicity, which implies that as the levels of the instrumental variable change this should have the same direction of effect on the treatment prescribed across similar individuals. However, this assumption cannot be verified.^58^ Indeed, in our study, we cannot observe the same treatment choice for a particular individual according to their attendance at two CCGs with different levels of prescribing preference for SGLT-2 inhibitors (versus DPP-4 inhibitors or sulfonylureas). For the population, this assumption implies that the average treatment choice must increase or decrease monotonically with the level of the instrumental variable.^59^ Hence it is plausible to assume that if a group of patients whose CCG had a moderate preference for prescribing SGLT-2 inhibitors were prescribed this drug class, then a similar group of patients whose CCG had a stronger preference for prescribing SGLT-2 inhibitors would not be prescribed DPP-4 inhibitors or sulfonylureas.^59^

We used the two stage residual inclusion method for the instrumental variable analysis,^60^ which enabled us to assess comparative effectiveness across the full study populations of interest—that is, to report average treatment effects while reducing the risk of bias from unmeasured confounding. The first stage models estimated the probabilities that each person was prescribed each treatment given their baseline covariates and their CCGs tendency to prescribe that treatment.^61^ The second stage outcome models then included generalised residuals from the first stage (propensity score) models. We estimated the outcome models by ordinary least squares for continuous outcomes (eg, HbA_1c_ level at one year) and by Cox proportional hazards models for time-to-event outcomes with an individual frailty.^32^ Models for both stages included all measured baseline covariates, with polynomials and covariate interactions selected through a post-double selection approach using least absolute shrinkage and selection operator regression^62^ ^63^ ^64^ (see supplementary methods table S1). The purpose of including person level covariates in the second stage (outcome regression) was to gain precision in estimating the relative treatment effects.

Some data were missing for outcomes (metabolic and other clinical measures) and baseline covariates (ethnicity, index of multiple deprivation, HbA_1c_, systolic blood pressure, diastolic blood pressure, BMI, eGFR, smoking and alcohol intake status) because the participants’ general practices either had not recorded these measures or had, but outside the requisite time window for a specific time point. At one year and two years, the percentages of missing values were, respectively, 33.7% and 36.4% for HbA_1c_, 44.7% and 47.8% for BMI, 33.6% and 37.2% for systolic blood pressure, and 37.4% and 40.0% for eGFR. For some people, a measurement that was not available at a particular time point (eg, two years) was available at other time points (eg, one year and three years) (see supplementary methods table S2). It was also possible that at any time point, one measure (eg, BMI) was not available, whereas other measures (eg, HbA_1c_, systolic blood pressure, and eGFR) were available.

We chose to handle all missing baseline and longitudinal outcome data by multiple imputation^65^ with chained equations.^66^ This approach assumed data were missing at random. The imputation of each longitudinal outcome at a given time point used all relevant information, including measurements of the same outcome at other time points. This use of auxiliary information can help the study recover more accurate estimates of the unknown outcome values.^67^ This also ensured our study population was comparable at each time point. Partially observed covariates and outcomes^67^ ^68^ were multiple imputed by predictive mean matching with 10 donors,^69^ producing five imputed datasets. The number of imputations was driven by the need to balance computational time with improved inference from increasing the number of imputations (see supplementary methods for further details). The imputation models developed for each covariate were congenial with the form of outcome^70^ (continuous or time to event). For the time-to-event endpoints, it was assumed no data were missing. All imputation models were stratified by second line treatment (DPP-4 inhibitors, SGLT-2 inhibitors, sulfonylureas) and by whether the individual died or was censored before the relevant study end date (see supplementary methods).

We reported differences between the comparison groups according to absolute change in outcomes between baseline and follow-up for continuous measures, and according to time-to-event measures. We reported results overall and according to whether patients had or did not have CVD (at least one of previous myocardial infarction, previous stroke, heart failure, ischaemic heart disease, or unstable angina) recorded before initiation of second line treatment. To recognise statistical uncertainty in the estimates of treatment effects, the data were bootstrapped 500 times, stratified by CCG, treatment group, and death and censoring status to maintain the structure of the original sample across replicates. Within each bootstrap resample we implemented multiple imputation with chained equations,^71^ ^72^ with Rubin’s first rule^65^ applied across the five imputed datasets to obtain overall treatment effects for each bootstrap sample, which we then used to estimate variances and calculate *t* based bootstrap confidence intervals (CI). The imputation procedure and time-to-event analyses were performed with multiple imputation with chained equations and the survival package^73^ ^74^ in R 4.2.2, respectively,^75^ and the analysis of the clinical measures in Stata 17.^76^

### Alternative analyses

We undertook alternative analyses to check the impact of different statistical assumptions on our results. Firstly, we applied complete case analysis rather than multiple imputation with chained equations (base case) to examine whether the results were robust when alternative approaches were applied to handle missing data. Secondly, we applied two stage least squares (continuous outcomes), multivariable linear regression (continuous outcomes), and Cox regression analysis (time to event), adjusting for all measured baseline covariates, to assess the sensitivity of our approach to confounding adjustment. Thirdly, we extended the follow-up period to five years rather than two years. Fourthly, in additional analyses that were not prespecified, we further checked the impact of applying approaches that, as with multivariable regression, assumed no unmeasured confounding but can be less sensitive to the form of outcome regression model. We applied two approaches based on propensity scores—inverse probability of treatment weighting^77^ and inverse probability of treatment weighting with regression adjustment (weighted regression hereafter),^78^ with non-stabilised and stabilised weights.^79^ We also used asymmetrical trimming to understand any effects of large weights in the weighted regression analysis.^80^ ^81^ The weighted regression has the so called double robustness property, in that, subject to the assumption of no unobserved confounding, it can still provide consistent estimates provided either the propensity score or the regression model is correctly specified.^78^ ^82^ The multivariable regression analyses, the inverse probability of treatment weighting, and the weighted regression analyses all estimate the average treatment effects as in the base case. We undertook the alternative analyses on the complete cases only.

### Patient and public involvement

Patient and public involvement advisors, including a coauthor on this paper (PC), helped inform the design and proposed analysis, including the choice of outcome measures. We will reconvene a patient and public involvement workshop to discuss the study findings and co-produce a lay summary that will be available on the PERMIT study website.^83^

## Results

### Study population and baseline characteristics

**Fig 2:**
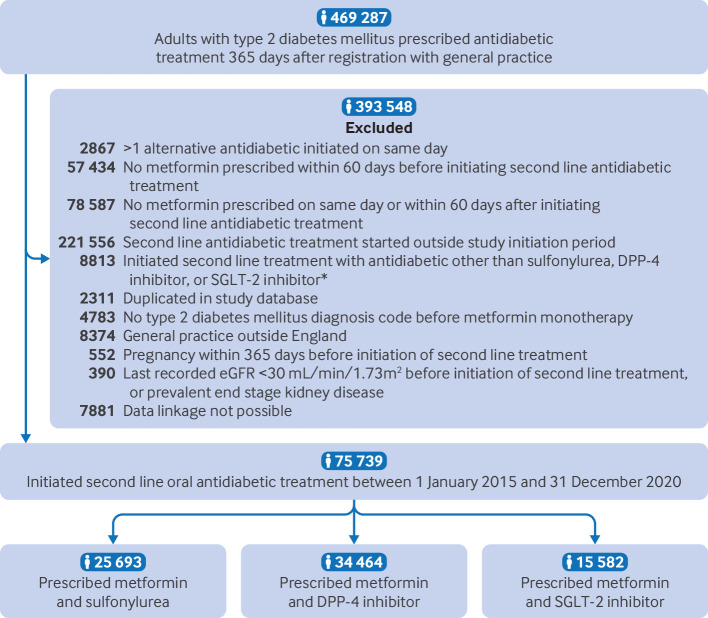
Flow of study population. *Other antidiabetic drugs prescribed as second line treatment included thiazolidinediones, glucagon-like peptide 1 receptor agonists, and insulin. DPP-4=dipeptidyl peptidase-4; SGLT-2=sodium-glucose cotransporter-2

The study population included 75 739 people with type 2 diabetes mellitus who initiated second line oral antidiabetic treatment with sulfonylureas, DPP-4 inhibitors, or SGLT-2 inhibitors and met all eligibility criteria (fig 2). Of these, 25 693 (33.9%) initiated treatment with sulfonylureas, 34 464 (45.5%) with DPP-4 inhibitors, and 15 582 (20.6%) with SGLT-2 inhibitors, in addition to metformin. Supplementary table 5 reports the frequencies of prescribing for each drug within each drug class. The drugs most commonly prescribed within each drug class were gliclazide (sulfonylurea), sitagliptin (DPP-4 inhibitor), and empagliflozin (SGLT-2 inhibitor). People prescribed SGLT-2 inhibitors were younger (56 (standard deviation (SD) 11) years) than those prescribed DPP-4 inhibitors (62 (SD 12) years) or sulfonylureas (60 (SD 13) years) (table 1). The baseline mean HbA_1c_ level was higher for people prescribed sulfonylureas (81 (SD 22) mmol/mol) compared with those prescribed DPP-4 inhibitors (72 (SD 16) mmol/mol) or SGLT-2 inhibitors (75 (SD 17) mmol/mol), and a lower proportion of people prescribed SGLT-2 inhibitors had comorbidities—for example, 17.2% (n=2680) of those prescribed SGLT-2 inhibitors had prevalent CVD compared with 22.8% (n=5858) of those prescribed sulfonylureas and 23.5% (n=8108) prescribed DPP-4 inhibitors. The proportion of people prescribed SGLT-2 inhibitors increased from 7.3% in 2015 to 24.9% in 2020. The median time between recorded BMI and the index date was 19 days (interquartile range (IQR) 0-140 days).

**Table 1:** Baseline characteristics of the primary-secondary care linked study population, stratified by prescribed second line antidiabetic treatment. Values are number (percentage) unless specified otherwise

Characteristics	Sulfonylureas (n=25 693)	DPP-4 inhibitors (n=34 464)	SGLT-2 inhibitors (n=15 582)
Female participants	9840 (38.3)	13 456 (39.0)	5996 (38.5)
Mean (SD) age (years)	60 (13)	62 (12)	56 (11)
Ethnicity:			
White	19 665 (76.5)	27 308 (79.2)	12 543 (80.5)
South Asian	3522 (13.7)	4616 (13.4)	1961 (12.6)
Black	1625 (6.3)	1451 (4.2)	542 (3.5)
Mixed/Other	534 (2.1)	612 (1.8)	231 (1.5)
Missing	347 (1.4)	477 (1.4)	305 (2.0)
Index of multiple deprivation fifth:			
1 (least deprived)	3619 (14.1)	5161 (15.0)	2604 (16.7)
2	4504 (17.5)	6175 (17.9)	2793 (17.9)
3	4955 (19.3)	6642 (19.3)	2953 (19.0)
4	6152 (23.9)	7677 (22.3)	3408 (21.9)
5 (most deprived)	6449 (25.1)	8785 (25.5)	3815 (24.5)
Missing	14 (0.1)	24 (0.1)	9 (0.1)
Year second line antidiabetic treatment was initiated:			
2015	6996 (27.2)	4958 (14.4)	1145 (7.3)
2016	5221 (20.3)	6057 (17.6)	1525 (9.8)
2017	4260 (16.6)	6309 (18.3)	2178 (14.0)
2018	3562 (13.9)	6771 (19.6)	2912 (18.7)
2019	3112 (12.1)	5995 (17.4)	3939 (25.3)
2020	2542 (9.9)	4374 (12.7)	3883 (24.9)
Median (IQR) No of years receiving metformin monotherapy	2.9 (1.1-5.4)	3.6 (1.7-6.3)	2.8 (1.3-5.2)
Median (IQR) mean No of patients registered with general practice	9690 (6250-13 628)	9971 (6538-13 795)	10 143 (6896-13 881)
Last HbA_1c_ value recorded before index date:			
Mean (SD) HbA_1c_ (mmol/mol)	81 (22)	72 (16)	75 (17)
Mean (SD) HbA_1c_ (%)	9.1 (2.1)	8.2 (1.5)	8.5 (1.6)
HbA_1c_ level (mmol/mol):			
<53	713 (2.8)	1053 (3.1)	515 (3.3)
53-74	10 818 (42.1)	21 870 (63.5)	8410 (54.0)
≥75	12 579 (49.0)	10 398 (30.2)	6134 (39.4)
Missing	1583 (6.2)	1143 (3.3)	523 (3.4)
Last blood pressure measure recorded before index date:			
Mean (SD) systolic blood pressure (mm Hg)	132 (14)	132 (14)	133 (14)
Mean (SD) diastolic blood pressure (mm Hg)	78 (9)	77 (9)	80 (9)
Hypertensive status based on last recorded blood pressure measure:			
Normotensive	7123 (27.7)	9424 (27.3)	3664 (23.5)
Hypertensive	18 525 (72.1)	25 002 (72.5)	11 906 (76.4)
Missing	45 (0.2)	38 (0.1)	12 (0.1)
Mean (SD) BMI at index date	31.5 (6.6)	32.2 (6.5)	35.1 (7.0)
BMI at index date:			
Under/normal weight	2718 (10.6)	2782 (8.1)	394 (2.5)
Overweight	8110 (31.6)	10 180 (29.5)	2867 (18.4)
Obese	14 702 (57.2)	21 375 (62.0)	12 283 (78.8)
Missing	163 (0.6)	127 (0.4)	38 (0.2)
Mean (SD) last recorded eGFR before index date (mL/min/1.73m^2^)	91 (19)	88 (19)	97 (15)
eGFR category (mL/min/1.73m^2^):			
Stage 1-2 (≥60)	23 282 (90.6)	30 823 (89.4)	15 186 (97.5)
Stage 3a-3b (30-59)	1770 (6.9)	3199 (9.3)	161 (1.0)
Missing	641 (2.5)	442 (1.3)	235 (1.5)
Comorbidities:			
Prevalent CVD	5858 (22.8)	8108 (23.5)	2680 (17.2)
Lower limb amputation	227 (0.9)	265 (0.8)	76 (0.5)
Heart failure	1457 (5.7)	2007 (5.8)	598 (3.8)
History of myocardial infarction	1644 (6.4)	2226 (6.5)	842 (5.4)
Previous stroke	1378 (5.4)	1678 (4.9)	512 (3.3)
Ischaemic heart disease	4572 (17.8)	6538 (19.0)	2175 (14.0)
Unstable angina	777 (3.0)	1099 (3.2)	362 (2.3)
History of any cancer	4254 (16.6)	5397 (15.7)	1447 (9.3)
Blindness	425 (1.7)	527 (1.5)	140 (0.9)
History of hypoglycaemia	260 (1.0)	302 (0.9)	129 (0.8)
Proteinuria	3658 (14.2)	4679 (13.6)	1585 (10.2)
Co-prescriptions:			
RAS inhibitor	12 584 (49.0)	18 911 (54.9)	8108 (52.0)
Statin	17 729 (69.0)	25 690 (74.5)	10 838 (69.6)
Smoking status:			
Non-smoker	5720 (22.3)	7455 (21.6)	3562 (22.9)
Former smoker	12 640 (49.2)	18 009 (52.3)	7865 (50.5)
Current smoker	7327 (28.5)	8992 (26.1)	4154 (26.7)
Missing	6 (0.0)	8 (0.0)	<5
Alcohol intake:			
Non-drinker	3192 (12.4)	3716 (10.8)	1630 (10.5)
Former drinker	7248 (28.2)	10 009 (29.0)	4179 (26.8)
Current drinker	14 899 (58.0)	20 367 (59.1)	9582 (61.5)
Missing	354 (1.4)	372 (1.1)	191 (1.2)

BMI=body mass index; CVD=cardiovascular disease; DPP-4=dipeptidyl peptidase-4; eGFR=estimated glomerular filtration rate; IQR=interquartile range; RAS=renin-angiotensin system; SD=standard deviation; SGLT-2=sodium-glucose cotransporter-2.

Within two years of follow-up, the median (IQR) time prescribed second line antidiabetic treatment was lower for those using sulfonylureas (248 (IQR 67-671) days) compared with DPP-4 inhibitors (345 (IQR 96-730) days) and SGLT-2 inhibitors (328 (IQR 84-730) days). The proportion of participants who switched to a third line treatment within two years of the index date was 58.8% (sulfonylureas, n=15 107), 51.5% (DPP-4 inhibitors, n=17 749), and 52.5% (SGLT-2 inhibitors, n=8184), with metformin monotherapy the most common third line treatment for all three comparison groups (see supplementary table 6). In each comparison group, the proportions of people whose third line treatment was triple therapy were 25.1% (sulfonylureas), 31.7% (DPP-4 inhibitors), and 21.8% (SGLT-2 inhibitors).

### Empirical assessment of instrumental variable assumptions

The tendency to prescribe met a major requirement for being a valid instrumental variable, in that it was strongly associated with the second line treatment prescribed (assumption 1), with accompanying F statistics of 1902 for DPP-4 inhibitors and 1935 for SGLT-2 inhibitors, which indicated that the instrumental variable was of sufficient strength (F>100).^38^ ^57^ The measured potential confounders were balanced across levels of the tendency to prescribe (assumption 2), aside from time period, which was included within the covariate adjustment of the instrumental variable analysis (see supplementary figures 2A-2C).

### Intermediate metabolic and other clinical measures

**Fig 3:**
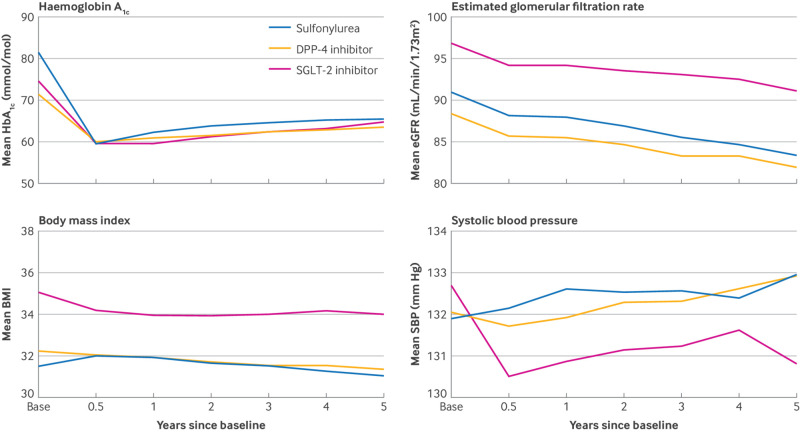
Mean HbA_1c_ (mmol/mol), estimated glomerular filtration rate (mL/min/1.73m^2^), body mass index, and systolic blood pressure (mm Hg) at each follow-up time point of interest, stratified by treatment group. DPP-4=dipeptidyl peptidase-4; eGFR=estimated glomerular filtration rate; HbA_1c_=glycated haemoglobin A_1c_; SBP=systolic blood pressure; SGLT-2=sodium-glucose cotransporter-2

The crude change in mean HbA_1c_ level from baseline to one year follow-up among people with observed follow-up measures was greatest for those prescribed sulfonylureas (−18 mmol/mol) compared with DPP-4 inhibitors (−10 mmol/mol) and SGLT-2 inhibitors (−14 mmol/mol; fig 3, also see supplementary figure 3). Of those people not censored by one year follow-up (n=72 066), 33.7% were missing HbA_1c_ values at this time point (see supplementary methods table 2). Although levels of missing data were higher for those time points that occurred after the onset of the covid-19 pandemic, the levels of missing data remained similar across the comparison groups (see supplementary table 7).

The crude changes in mean BMI and systolic blood pressure from baseline were small across all time points (fig 3, also see supplementary figure 3). The crude change in mean eGFR from baseline to one year follow-up was similar across the three second line treatments of interest (−2 mL/min/1.73m^2^), with smaller decreases in mean eGFR across subsequent follow-up periods among people prescribed SGLT-2 inhibitors rather than sulfonylureas or DPP-4 inhibitors (fig 3, also see supplementary figure 3).

**Fig 4:**
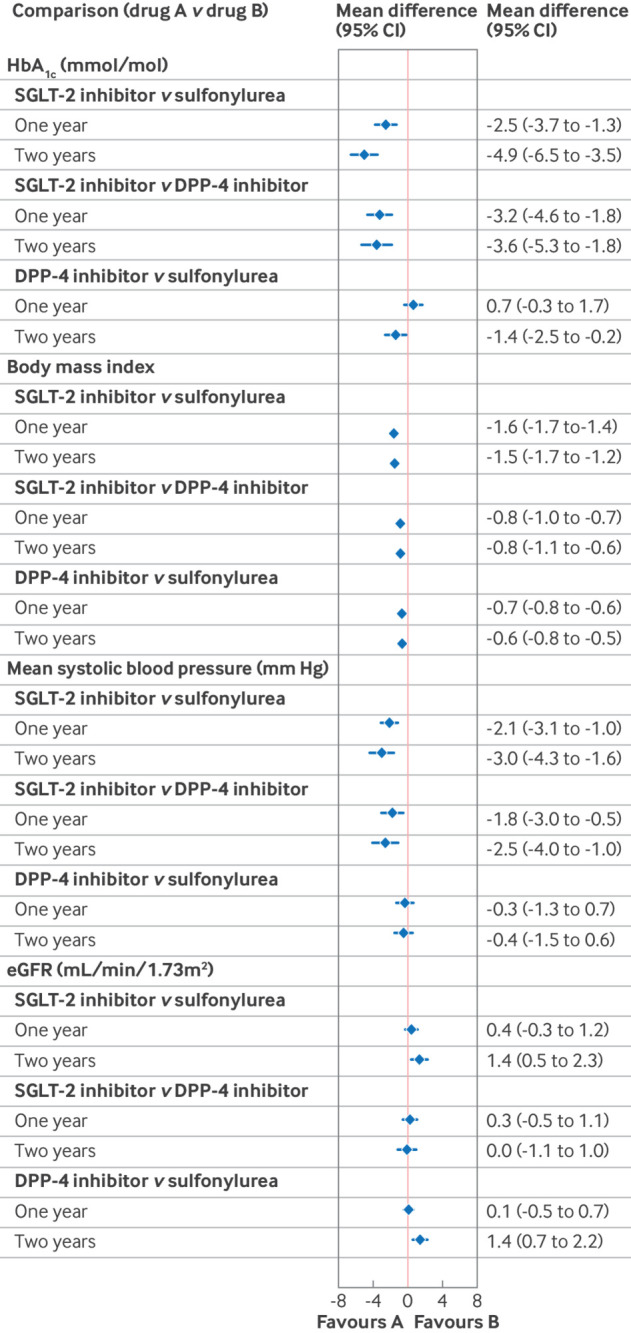
Forest plot showing differences in the mean change between baseline and one year or two years follow-up in continuous clinical measures comparing second line antidiabetic treatments from the instrumental variable analysis to reduce the risk of confounding, and with multiple imputation to account for missing data, which assumes data are missing at random. . CI=confidence interval; DPP-4=dipeptidyl peptidase-4; eGFR=estimated glomerular filtration rate; HbA_1c_=glycated haemoglobin A_1c_; SGLT-2=sodium-glucose cotransporter 2

Figure 4 presents the results from the instrumental variable analysis, which reduces the risk of confounding, and after applying multiple imputation with chained equations to handle the missing data. The results apply to the full study population. Strong evidence was found for SGLT-2 inhibitors being more effective in reducing HbA_1c_ levels between baseline and one year follow-up, with a mean reduction of −2.5 mmol/mol (95% CI −3.7 to −1.3) compared with sulfonylureas and −3.2 mmol/mol (−4.6 to −1.8) compared with DPP-4 inhibitors (fig 4, also see supplementary table 8). After accounting for confounding and missing data, SGLT-2 inhibitors were more effective in improving BMI and systolic blood pressure (fig 4). People prescribed SGLT-2 inhibitors showed a greater reduction in BMI between baseline and one year, with a mean difference of −1.6 (95% CI −1.7 to −1.4) compared with sulfonylureas and −0.8 (−1.0 to −0.7) compared with DPP-4 inhibitors. For systolic blood pressure, the mean difference was −2.1 mm Hg (95% CI −3.1 to −1.0) compared with sulfonylureas and −1.8 mm Hg (−3.0 to −0.5) compared with DPP-4 inhibitors, with these improvements maintained at two years follow-up. SGLT-2 inhibitors led to a slower decline in eGFR at two years follow-up compared with sulfonylureas (mean difference 1.4 mL/min/1.73m^2^, 95% CI 0.5 to 2.3), but not compared with DPP-4 inhibitors (0.0 mL/min/1.73m^2^, −1.1 to 1.0).

### Kidney, cardiovascular, and mortality outcomes

**Fig 5:**
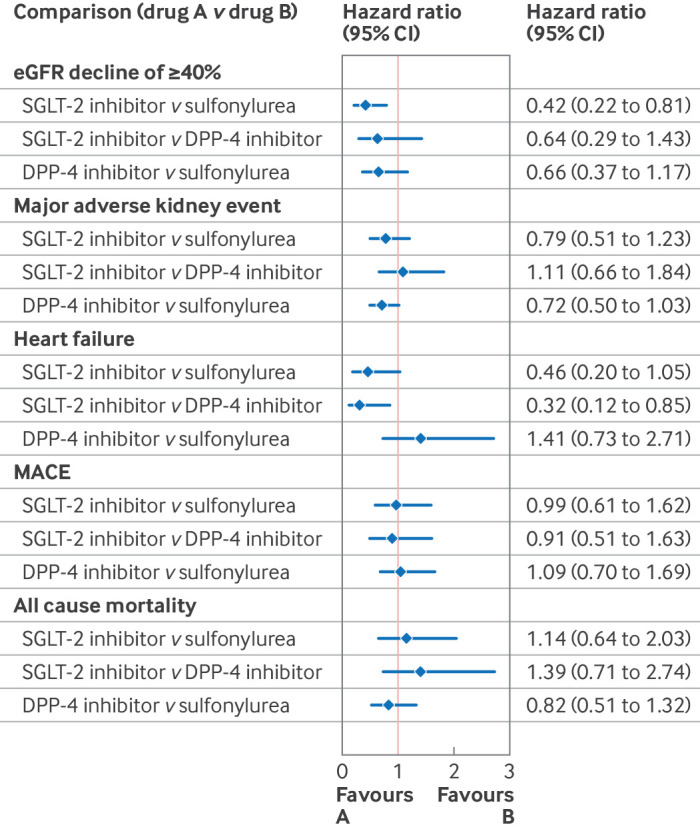
Forest plot showing adjusted hazard ratios of cardiovascular disease and kidney outcomes comparing second line antidiabetic treatments from the instrumental variable analysis to reduce the risk of confounding, and with multiple imputation to account for missing data, which assumes data are missing at random. . CI=confidence interval; MACE=major adverse cardiovascular event (composite for the earliest of myocardial infarction, stroke, or cardiovascular death); eGFR=estimated glomerular filtration rate; DPP-4=dipeptidyl peptidase-4; SGLT-2=sodium-glucose cotransporter 2

People prescribed SGLT-2 inhibitors had lower crude rates of all adverse kidney, cardiovascular, and mortality events compared with those prescribed sulfonylureas and DPP-4 inhibitors (see supplementary table 9 and supplementary figures 4-9). After reducing the risk of confounding and addressing the missing data, we found that over two years follow-up (base case), SGLT-2 inhibitors were more effective in preventing a ≥40% decline in eGFR from baseline versus sulfonylureas (hazard ratio 0.42, 95% CI 0.22 to 0.81), but the estimated hazard ratios for SGLT-2 inhibitors compared with DPP-4 inhibitors were highly uncertain (0.64, 0.29 to 1.43) (fig 5). The rates of admission to hospital for heart failure were lower for SGLT-2 inhibitors compared with sulfonylureas (0.46, 0.20 to 1.05) and with DPP-4 inhibitors (0.32, 0.12 to 0.85). For the other endpoints, we found no evidence of a difference in the comparative effectiveness of the second line antidiabetic treatments (fig 5, also see supplementary table 10). We found no evidence that having CVD before starting second line treatment was associated with modified relative effectiveness of these three treatments (see supplementary tables 11 and 12).

### Alternative analyses

The findings of the complete case analyses were similar when applying multiple imputation to deal with missing data (see supplementary tables 10 and 13). The results were also similar if the risk of confounding was dealt with using two stage least squares, an alternative instrumental variable approach (see supplementary table 14). The regression analyses that assumed no unmeasured confounders existed reported that the benefits of SGLT-2 inhibitors were greater than for the base case and more precisely estimated (see supplementary tables 10 and 15). When the study time frame was extended to five years, the gains after initial receipt of SGLT-2 inhibitors were maintained, although by this time point few people had complete follow-up information or were still prescribed the same second line treatment (see supplementary tables 6, 8, 10, and 13-15). The results were similar to the main analyses if inverse probability of treatment weighting or weighted regression were used to reduce the risk of confounding due to observed baseline covariates (see supplementary tables 16-20 and supplementary figures 10 and 11).

## Discussion

In this comparative effectiveness study, we found that second line treatment with SGLT-2 inhibitors for people with type 2 diabetes mellitus was more effective than sulfonylureas or DPP-4 inhibitors in reducing mean HbA_1c_ levels, BMI, and systolic blood pressure after the risk of confounding was reduced using an instrumental variable analysis. SGLT-2 inhibitors were also more effective at reducing the hazards of hospital admission for heart failure (compared with DPP-4 inhibitors) and ≥40% decline in eGFR (compared with sulfonylureas). We did not find strong evidence for other meaningful differences for the other study endpoints over the two year study period.

A major concern of any study aiming to assess comparative effectiveness from routine data is bias from confounding, particularly unmeasured prognostic differences between comparison groups. This risk of bias can never be eliminated. A crucial advantage of our study design, however, was that it followed recommended methods of target trial emulation in prespecifying the population eligibility criteria, time zero, treatment comparisons, outcomes, and analyses.^41^ ^84^ ^85^ In our main analysis we used an instrumental variable to further reduce the risk of residual confounding. We were therefore able to provide useful evidence about the comparative effectiveness of these three treatments as they were prescribed in routine clinical practice for a diverse population of people with type 2 diabetes mellitus.

The aim of the PERMIT study was to assess the relative effectiveness of the three most common second line treatments for an unselected population in routine clinical practice. In contrast, published randomised controlled trials have aimed to show the safety and efficacy of one of these drug classes compared with placebo in selected populations. For the comparison of SGLT-2 inhibitors versus placebo, published randomised controlled trials do not include general populations of people with type 2 diabetes mellitus who meet the eligibility criteria of national guidelines for these three second line treatments (see supplementary table 21).^7^ It is therefore challenging to compare the results of the PERMIT study with those of the published randomised controlled trials.

In supplementary tables 21-22, we describe the results of the PERMIT study alongside those of the corresponding randomised controlled trials for common endpoints such as hospital admission for heart failure, MACE, major adverse kidney events, and all cause death. We found that the point estimates for the PERMIT target trial emulation fall within the estimated 95% CI of the corresponding treatment effects reported in the randomised controlled trials—that is, they met previously defined criteria for agreement^85^ (see supplementary table 22). This concordance also applied to the few published randomised controlled trials, including the GRADE trial,^29^ ^36^ that compared two active treatments—DPP-4 inhibitors and sulfonylureas for general populations of people with type 2 diabetes mellitus. Unlike the GRADE trial, the PERMIT study did not exclude people with HbA_1c_ levels outside the range 6.8-8.5%. A previous target trial emulated the GRADE trial in applying strict eligibility criteria, but, unlike our study, it was unable to investigate MACE, heart failure, and all cause mortality owing to low event rates from a small study population. Our larger study found protective effects of SGLT-2 inhibitors for heart failure compared with DPP-4 inhibitors, similar to meta-analyses of randomised controlled trials 86 87 and observational studies.^32^ However, even with this relatively large sample, the number of people followed over the full follow-up period was insufficient to detect other clinically important differences for outcomes such as major adverse kidney events, and to investigate end stage kidney disease and CVD specific mortality individually.

In our alternative analysis, we made the common assumption of no unmeasured confounding, and found that after adjusting for all measured confounders, SGLT-2 inhibitors were associated with greater improvement in all endpoints, including all cause mortality. People prescribed SGLT-2 inhibitors, however, had fewer comorbidities and were likely to be healthier according to unmeasured baseline characteristics. A previous study that considered uptake of SGLT-2 inhibitors as second line antidiabetic treatment also reported that compared with people who received sulfonylureas or DPP-4 inhibitors, those who received SGLT-2 inhibitors were healthier and at lower risk of all cause death.^34^ For an endpoint such as all cause death, it is particularly challenging to capture all the potential confounders from routine data sources (see supplementary figure 1B). For this endpoint, important potential confounders include the individual’s overall health, diet, exercise, and lifestyle before second line treatment. If an instrumental variable is valid, it reduces the risk of bias from these unmeasured confounders, whereas approaches such as regression do not. Hence, the finding from the regression analysis that within the two year follow-up period SGLT-2 inhibitors were associated with reduced hazards of all cause mortality compared with sulfonylureas or DPP-4 inhibitors could reflect these unmeasured baseline differences (ie, residual confounding).

### Strengths and weaknesses and comparison with other studies

In this study, we directly compared the three most commonly prescribed second line antidiabetic drug treatments using a large, linked dataset that is representative of the UK primary care population in terms of age and sex.^44^ ^45^ Our direct comparison of sulfonylureas, DPP-4 inhibitors, and SGLT-2 inhibitors contrasts with previous trials^16^ ^17^ ^18^ ^20^ ^26^ ^29^ ^36^ and meta-analyses,^86^ ^87^ which did not include an active second line treatment as a comparator. We did not restrict the study population to those with baseline HbA_1c_ levels in a particular range, as has been done in many previous randomised controlled trials.^16^ ^17^ ^18^ ^19^ This study therefore includes people with a broader range of glycaemic control, which is reflective of the UK primary care population with type 2 diabetes mellitus.

We add to the evidence reported in previous observational studies,^33^ ^34^ ^35^ ^40^ ^88^ which make direct comparisons between antidiabetic treatments, by using an instrumental variable analysis as the main analysis to reduce the risk of confounding from both measured and unmeasured baseline confounders, and we provide evidence on comparative effectiveness for those three drug classes that are most commonly prescribed in publicly funded health systems for a general population of people with type 2 diabetes mellitus. We investigated not only intermediate metabolic and other clinical measures but also adverse kidney and cardiovascular events, which are important to patients. The benefits we observed of SGLT-2 inhibitors improving HbA_1c_ levels, BMI, and systolic blood pressure and reducing the risks of admission to hospital for heart failure (compared with DPP-4 inhibitors) and ≥40% decline in eGFR (compared with sulfonylureas) are indicative of a causal mechanism that has some biological plausibility.

Our directed acyclic graphs provided a framework for the analysis, which recognised that second line treatment with SGLT-2 inhibitors in routine practice could improve any of the intermediate clinical endpoints listed and which may in turn lead to reduced risks of subsequent events. In particular, the pharmacological action of SGLT-2 inhibitors—namely, reducing blood pressure and cardiac preload and after load through diuretic mechanisms,^89^ would imply protective effects on hospital admissions for heart failure and on kidney endpoints; however, this would not necessarily translate to immediate protective effects during an ST elevated myocardial infarction or acute rupture of a coronary plaque.

We acknowledge limitations in our study. We did not consider glucagon-like peptide-1 receptor agonists because this class was rarely prescribed as a second line antidiabetic treatment in the UK during the study period,^12^ ^13^ and they are still not recommended as second line treatment for people with type 2 diabetes mellitus.^12^ The prescribing of glucagon-like peptide-1 receptor agonists is increasing in the US, however, and warrants further study as the number of people prescribed these drugs increases in routinely collected data. Our instrumental variable analysis relied on three major assumptions. Although we were able to empirically show that the instrument strongly predicts receipt of treatment (assumption 1), we could only partially evaluate whether the instrument was balanced across confounders (assumption 2). We adjusted for measured confounders in the second stage of the regression model to account for any residual imbalances across levels of the instrument, in particular according to time period and contextual measures such as region and general practice size. If, however, assumption 2 is not met, then unmeasured confounders would be imbalanced across levels of the instrument, leading to biased estimates. We must also assume that the instrument, the tendency to prescribe, does not directly impact outcomes except through the treatment prescribed (assumption 3). We could not test this assumption, and it is possible it would be violated if, for example, after adjusting for region and practice size, those CCGs that had a higher tendency to prescribe SGLT-2 inhibitors also delivered higher quality of care that improved outcomes independent of the second line treatment prescribed.

The PERMIT study used routine data, and the requisite outcome data were not available for all those included. For continuous measures, the proportion of people with missing values at the one year time point ranged from 33.7% (HbA_1c_) to 44.7% (BMI). In the main analysis we dealt with these missing data for all the continuous outcomes along with any missing information on covariates with multiple imputation, and we undertook complete case analysis as alternative analyses. The results from these alternative approaches that make different underlying assumptions about why the data were missing were similar. For the time-to-event endpoints, we used linked primary and secondary care and ONS death datasets to ascertain cardiovascular, kidney, and mortality outcomes to improve the capture of events, rather than relying on a single source. However, a limitation shared with other target trial emulations using routine data is that it is not known if data on events pertaining to kidney disease or CVD is missing. People may experience an event that is diagnosed and recorded in outpatient clinics but not recorded in the linked primary-secondary care (inpatient) data. For major events such as myocardial infarction or stroke, levels of under-recording in the linked data are likely to be small and similar across the comparison groups and lead to reduced statistical power rather than bias in the estimates of relative effectiveness.

Although the study did consider endpoints up to five years after initiation of second line treatment, by this time point levels of missing data were high (from 46.9% for HbA_1C_ to 59.4% for BMI), and after two years most people will have stopped their second line treatment. Hence, although we have reported results for the prespecified five year endpoint, caution is needed when interpreting these results, given the levels of missing data.

### Policy implications

This study provides evidence that SGLT-2 inhibitors might offer clinically important benefits when provided in routine clinical practice compared with common alternative oral antidiabetic drugs that are added to metformin for people with type 2 diabetes mellitus. These findings apply to a wide range of people with type 2 diabetes mellitus and therefore complement the evidence available from randomised controlled trials^16^ ^17^ ^18^ ^19^ ^20^ ^21^ ^22^ ^23^ ^24^ and previous studies that have emulated trials.^35^ ^40^ In recent updated guidelines, NICE and other health technology assessment agencies have published guidance and guidelines that are neutral about the use of SGLT-2 inhibitors versus DPP-4 inhibitors versus sulfonylureas as second line treatments, except for people at high risk of CVD, or for people with pre-existing CVD, including heart failure, or with kidney disease. For these subgroups, SGLT-2 inhibitors are recommended in addition to metformin. Our study reported similar advantages for SGLT-2 inhibitors (compared with sulfonylureas and DPP-4 inhibitors) as second line treatments for people who did not have pre-existing CVD as well as for those who did have CVD before second line treatment. Future guidelines could draw from this study and related evidence to also recommend SGLT-2 inhibitors for those without CVD, including those at relatively low risk of subsequent CVD.

More work is needed to understand the long term effectiveness and cost effectiveness of increasing the use of SGLT-2 inhibitors for people with type 2 diabetes mellitus. Future research can use the information from this study to predict whether SGLT-2 inhibitors can lead to sufficient improvement in long term outcomes—for example, from reduced incidence and costs of complications such as retinopathy, amputation, or end stage kidney disease, to justify any additional costs. Further research is also required to assess the comparative effectiveness of glucagon-like peptide-1 receptor agonists with the three alternative second line oral antidiabetic treatments among people with type 2 diabetes mellitus, and to assess how best to personalise the order in which these treatments are prescribed.

### Conclusions

We found that for a broad population of people with type 2 diabetes mellitus, SGLT-2 inhibitors were more effective second line treatments in routine clinical practice compared with DPP-4 inhibitors or sulfonylureas in improving HbA_1c_ levels, BMI, and systolic blood pressure. SGLT-2 inhibitors were also found to be more effective at reducing the hazards of hospital admission for heart failure (compared with DPP-4 inhibitors) and ≥40% decline in eGFR (compared with sulfonylureas). We did not find evidence for differences in the other study endpoints over the two year study period.

**What is already known on this topic**

Placebo controlled randomised trials showed that sodium-glucose cotransporter-2 (SGLT-2) inhibitors are cardioprotective and kidney protective among people with type 2 diabetes mellitus (T2DM)
NICE guidelines recommend SGLT-2 inhibitors with metformin as second line oral antidiabetic treatment for people with T2DM and cardiovascular disease CVD), or at high risk of CVD; however, for the broader population with T2DM without these indications, these guidelines recommend sulfonylureas, DPP-4 inhibitors, or SGLT-2 inhibitors along with metformin
The comparative effectiveness of these three second line treatments has not been assessed directly in randomised controlled trials, and evidence from observational studies is prone to confounding by indication

**What this study adds**

SGLT-2 inhibitors were found to be more effective than sulfonylureas or DPP-4 inhibitors in lowering mean haemoglobin A_1c_ levels, body mass index, and systolic blood pressure for a broad population of people with T2DM
SGLT-2 inhibitors were found to be more effective than sulfonylureas or DPP4-inhibitors in reducing the hazards of hospital admission for heart failure (*v* DPP-4 inhibitors) and kidney disease progression (*v* sulfonylureas) for a broad population of people with T2DM
A target trial design was combined with an instrumental variable analysis to help reduce the risk of bias from confounding and to supplement previous studies in providing useful evidence that applies directly to routine clinical practice

## Data Availability

Owing to data sharing restrictions, the data used in this study cannot be shared directly. Researchers may, however, apply to use Clinical Practice Research Datalink (CPRD) data linked with other health datasets. Further instructions are available on the CPRD website (https://cprd.com/). Codelists to create exposure, outcome, and covariates are published on LSHTM DataCompass (https://datacompass.lshtm.ac.uk/id/eprint/3743/ ).

## Web extra

Extra material supplied by authors

## Notes

Contributors: PB, DGLP, OC, SON, AB, KK, AIA, and RG conceived and designed the study. All authors critically appraised and contributed to the study design and analysis plan. PB requested and extracted the data. PB, DGLP, OC, and SON performed the data management and analysis. PB, DGLP, OC, SON, and RG wrote the manuscript with contributions from all authors. All authors critically revised and approved the manuscript. PB and RG are the guarantors. PB and DGLP share first authorship and contributed equally. The corresponding author attests that all listed authors meet authorship criteria and that no others meeting the criteria have been omitted.

Transparency: The lead authors (PB and DGLP) and the manuscript’s guarantors (PB and RG) affirm that the manuscript is an honest, accurate, and transparent account of the study being reported; that no important aspects of the study have been omitted; and that any discrepancies from the study as originally planned have been explained.

Dissemination to participants and related patient and public communities: After publication, the results will be disseminated to policy makers, including those at the National Institute for Health and Care Excellence. We have shared preliminary methodology and results related to this work with two patient panels. With the help of our patient and public involvement and clinical colleagues, we will disseminate the study results after publication with patients, the public, and healthcare professionals through press releases, media communications, social media postings, and presentations at scientific conferences.

Provenance and peer review: Not commissioned; externally peer reviewed.
