# Supplementary material for: Comparative effectiveness of second line oral antidiabetic treatments among people with type 2 diabetes mellitus: emulation of a target trial using routinely collected health data
Source: BMJ. 2024 May 8;385:e077097. doi: 10.1136/bmj-2023-077097 (PMC11077536; doi:10.1136/bmj-2023-077097)
Supplement: Supplementary file 1 — Supplementary information: Supplementary methods, tables, and figures [file bidp077097.ww.pdf]

Comparative effectiveness of alternative second-line oral antidiabetic treatments on metabolic, kidney, and cardiovascular outcomes amongst people with type 2 diabetes mellitus: a cohort study using routinely collected health data

Supplementary materials

SUPPLEMENTARY METHODS 4

Supplementary methods table S1: Summary of (i) analysis variables adjusted for in the continuous and survival analyses, (ii) survival information included in the imputation models for the analysis variables, and (iii) continuous outcome information included in the imputation models for the analysis variables 6

Supplementary methods table S2: Percentage of observations which are missing at time point t after accounting for censoring 7

Supplementary methods table S3: Fitted imputation models for each partially-observed variable used in the continuous and survival analyses. Analysis covariates, Survival information and Continuous outcomes are specified in **Supplementary methods table 1**. 9

SUPPLEMENTARY TABLES 12

Supplementary table 1: Summary of target trial emulation 13

Supplementary table 2: Details of inclusion and exclusion criteria for the study population 17

Supplementary table 3: Details of covariate data sources and definitions 18

Supplementary table 4: Details of outcome data sources and definitions 21

Supplementary table 5: Frequency of individual drug substances within each drug class included as the treatments of interest in this study 23

Supplementary table 6: Time spent on second-line antidiabetic treatment, overall and stratified by treatment group 24

Supplementary table 7: Describing the proportion of the study population who initiate second-line oral antidiabetic treatment during the COVID-19 pandemic, and corresponding missingness in the primary outcome by treatment group 26

Supplementary table 8: Differences in the change in continuous clinical measures for the three second-line antidiabetic treatment comparisons for the main analysis (2SRI, bootstrap-multiple imputation) 27

Supplementary table 9: Crude event counts and rates (95% CI) for time-to-event kidney and cardiovascular outcomes up to 2-years follow-up 29

Supplementary table 10: Summary of results from main analysis for kidney, cardiovascular, and mortality time-to-event outcomes, as well as summary of results for alternative analyses for kidney, cardiovascular, and mortality outcomes 30

Supplementary table 11: Differences in the change in continuous clinical measures for the three second-line antidiabetic treatment comparisons according to CVD status prior to initiation of second-line treatment (2SRI, bootstrap-multiple imputation) 33

Supplementary table 12: Summary of results from the subgroup analysis comparing kidney, cardiovascular, and mortality outcomes according to CVD status prior to initiation of second-line treatment 35

Supplementary table 13: Differences in the change in continuous clinical measures for the three second-line antidiabetic treatment comparisons for 2SRI analysis (complete cases) 37

Supplementary table 14: Differences in the change in continuous clinical measures for the three second-line antidiabetic treatment comparisons for 2 stage-least squares (2SLS) instrumental variable analysis on complete cases 39

Supplementary table 15: Differences in the change in continuous clinical measures for the three second-line antidiabetic treatment comparisons for ordinary least squares (OLS) regression adjusted for measured confounders (complete cases) 41

Supplementary table 17: Differences in the change in continuous clinical measures for the three second-line antidiabetic treatment comparisons (inverse probability of treatment weighting (IPTW), complete cases) 49

Supplementary table 18: Differences in the change in continuous clinical measures for the three second-line antidiabetic treatment comparisons (inverse probability of treatment weighting (IPTW)-weighted regression, complete cases) 51

Supplementary table 19: Main features of the study populations and comparison groups for the PERMIT study and relevant randomised controlled trials (RCTs) that included a randomisation to either SGLT2i or DPP4i 54

Supplementary table 20: Results from main analysis of the PERMIT study and those from relevant RCTs for common endpoints 56

SUPPLEMENTARY FIGURES 58

Supplementary figure 1A: Directed acyclic graph (DAG) illustrating the causal relationship between the instrument, exposure, and primary outcome (change in HbA1c from baseline to 1-year follow-up)............59

Supplementary figure 1B: Directed acyclic graph (DAG) illustrating the causal relationship between the instrument, exposure, and all-cause mortality (secondary outcome) 61

Supplementary figure 2A: Covariate balance plots according to levels of the instrumental variable for SU 63

Supplementary figure 2B: Covariate balance plots according to levels of the instrumental variable for DPP4i 66

Supplementary figure 2C: Covariate balance plots according to levels of the instrumental variable for SGLT2i............69

Supplementary figure 3: Mean change in HbA1c (mmol/mol), eGFR (mL/min/1.73m^2), BMI (kg/m^2), and SBP (mm Hg) from baseline during follow-up............72

Supplementary figure 4: Cumulative failure curve for time to 40% decline in eGFR from baseline up to 2 years follow-up by exposure status............73

Supplementary figure 5: Cumulative failure curve for end-stage kidney disease (ESKD) up to 2-years follow-up stratified by exposure status............74

Supplementary figure 6: Cumulative failure curve for major adverse kidney event (MAKE) up to 2-years follow-up by exposure status 75

Supplementary figure 7: Cumulative failure curve for heart failure hospitalisation up to 2-years follow-up by exposure status 76

Supplementary figure 8: Cumulative failure curve for 3-point major adverse cardiovascular event (MACE) by exposure status 77

Supplementary figure 9: Cumulative failure curve for all-cause mortality by exposure status............78

Supplementary figure 10: Propensity score distribution before adjustment for the inverse probability of treatment weighting analysis (IPTW)............79

Supplementary figure 11: Propensity score distribution after adjustment for the inverse probability of treatment analysis (IPTW) 80

SUPPLEMENTARY METHODS

INSTRUMENTAL VARIABLE ANALYSIS

We followed a two-stage residual inclusion (2SRI) approach in the main analysis to reduce the risk of bias from unmeasured confounding and allow us to identify the average treatment effects (ATEs). We compared these estimates to those from using 2-stage least squares (2SLS) (alternative analysis).

2-stage least squares (2SLS):

In the first stage, we estimated linear probability models by regressing indicators for each of the treatments of interest (DPP4i or SGLT2i using SU as the reference category) on the instruments (TTP for either treatment), and the baseline covariates including NHS region and time period. In the second stage, we regressed the outcome of interest on the baseline covariates and the predicted probability of being prescribed each treatment obtained from the first stage. The two stages were estimated jointly so that standard errors reflected the uncertainty of both stages. However, when effects are heterogeneous, that is they vary with respect to observed or unobserved covariates, this approach estimates the *local* average treatment effects (LATE) since the effects relate to the compliers whose treatment assignment is altered by the instrument (or in the case of a continuous IV, a weighted average of LATEs), which is less relevant for decision making compared with the overall average treatment effect. We therefore consider 2SRI to be the more informative approach here since estimates relate to the full population provided the first stage model is correctly specified.

2-stage residual inclusion:

For DPP4i and SGLT2i, we estimated first-stage probit models for whether or not the patient was prescribed this treatment, as a function of the baseline covariates and the tendency to prescribe that treatment. For continuous outcomes (e.g., HbA1c at 12 months), we then estimated a second stage regression model, using ordinary least squares including the generalised residuals[1] from the first stage models, all measured baseline covariates. For the censored time-to-event outcomes (e.g., time to 3-point MACE), in the second stage we estimated Cox proportional hazards models that account for individual frailty,[2] in addition to covariates and the observed residuals from the first stage models.[3]

Variable selection:

In addition to the covariates listed, the 2SRI and 2SLS models also considered the quadratic forms of age and baseline HbA1c as well as two sets of interactions. The first set of interactions are those between baseline HbA1c with age, sex and baseline BMI. The second set of interactions are the products of the IV (for the first stage models) or the treatment indicator variables (for the second stage models) with baseline HbA1c, eGFR, BMI, systolic blood pressure and age.

To prevent overfitting the models, we used the Least Absolute Shrinkage and Selection Operator (LASSO) regression algorithm[4, 5] to inform which of the interactions above are relevant in each case. The LASSO aims to find the set of coefficients that minimise the sum-of-squares loss function subject to a constraint on the sum of absolute values of coefficients. This results in a linear regression in which only a small number of covariates have non-zero coefficients that can then be included in the model in question. Specifically, we used the 'rigorous LASSO' approach[6] which places a high priority on controlling overfitting, thus often producing parsimonious models.

By partialling out variables prior to penalisation, we ensure these variables are always included in the selected models and only penalise (and potentially discard) variables in the interaction sets. We use the 'rigorous LASSO' approach[6] which places a high priority on controlling overfitting, thus often producing parsimonious models.[7] By partialling out variables prior to penalization, we ensure these variables are always included in the selected models and only penalise (and potentially discard) variables in the interaction sets.[7]

To select the variables included in the estimation of the 2SRI models, we estimated the first and second stage models for each outcome using rigorous LASSO. The final set of covariates used to estimate effects for each outcome included all the covariates mentioned in the *Covariates* section plus the interactions that were selected in at least one model of the respective outcome. For the Cox proportional hazards models, all final selected covariates were assessed for violation of the proportional hazards assumption using Schoenfeld residuals.

Estimating treatment effects:

From the second stage models using the selected variables, we calculated the difference in the average absolute change in predicted outcomes (continuous measures) and times to event between the comparison groups, providing estimates of the treatment effect according to individual-level covariates. We aggregate these estimates to report results overall and according to whether or not patients had pre-existing CVD (at least one of previous MI, previous stroke, CHF, IHD, or unstable angina).

All standard errors were calculated with non-parametric bootstrapping as described below, and accounted for clustering of individuals within NHS region, treatment arm and censoring status.

HANDLING OF MISSING DATA, CENSORING, AND LOSS TO FOLLOW-UP

The PERMIT study uses routine linked data (CPRD and HES) which raises several challenges for the statistical analysis. Missing data may occur due to:

- Non-attendance at a GP within the requisite time period for the study outcome definition (+/- 3 months either side of timepoints 0.5, 1, 2, 3, 4 and 5 years)
- Information not being recorded during a GP visit
- Tests not being done during a GP visit

In addition to missing data, all patients are not fully followed from baseline to five years. For example, a patient enrolled in December 2020 will have 12 months follow-up to 2021 and can only be included in the analysis models for the continuous outcomes for the periods between baseline and 6 months or 1 year, as they are unobserved for subsequent timepoints. The final challenge is related to 'loss to follow-up' or 'dropouts', as a patient may stop attending GP appointments before death or censoring (end of follow-up or patient/GP stops contributing to CPRD) has occurred.

Supplementary methods table 1 presents the full list of covariates which are adjusted for in both sets of analyses, summarises the seven survival outcomes and the four continuous clinical outcomes measures at timepoints 0.5, 1, 2, 3, 4 and 5 years.

Supplementary methods table S1: Summary of (i) analysis variables adjusted for in the continuous and survival analyses, (ii) survival information included in the imputation models for the analysis variables, and (iii) continuous outcome information included in the imputation models for the analysis variables

Analysis Covariates		
Baseline age	Sex	Ethnicity
Index of multiple deprivation	Days since 2nd line treatment assignment	Practice size in 2014
Renin	Statin	Myocardial Infarction
Unstable Angina	Stroke	Hypoglycaemia
Heart Failure	Cancer history	Proteinuria history
Advanced eye disease	Lower extremity amputation	Lower extremity amputation
CKD	Baseline HbA1c	Baseline systolic blood pressure
Baseline diastolic blood pressure	Baseline eGFR	Baseline BMI

Smoking status	Alcohol status	Year of first 2 line initiation
Practice region	IHD	Hospital attendance in last year
Age-squared	HbA1c-squared	HbA1c*Baseline Age
HbA1c*BMI	HbA1c*Sex	
Nelson-Aalen Estimates & Event indicator information		
MACE	MI	Stroke
Heart Failure hospitalisation	Death	End stage kidney disease (ESKD)
eGFR decline from 40%	Composite kidney	
Continuous clinical measures for time t= 0.5, 1, 2, 3, 4 and 5 years		
Change in HbA1c at time *t*	Change in eGFR at time *t*	Change in BMI at time *t*
Change in SBP at time *t*		

Missing data is present in in both the continuous outcomes, and also several covariates which are used in the analysis of the continuous and survival outcomes. The percentage of missing values in the analysis covariates are available in Table 1 of the main paper. **Supplementary methods table 2** presents the percentage missing for those not censored at time *t* for each clinical measure. The longitudinal clinical measures (HbA1c, BMI, SBP, eGFR) could be unavailable at any timepoint (6 months, 1, 2, 3 or 5 years). For example, a patient could have observed HbA1c values at all time points except for year 2.

Supplementary methods table S2: Percentage of observations which are missing at time point t after accounting for censoring

**Time *t***	**N**	**Percentage (%) missing in continuous measures at timepoint *t***			
		**HbA1c**	**BMI**	**SBP**	**eGFR**
6 months	72,066	32.1	50.0	39.0	42.2
1 year	66,702	33.7	44.7	33.6	37.4
2 years	52,962	36.4	47.8	37.2	40.0
3 years	39,099	38.6	50.0	39.6	42.1
4 years	26,366	40.6	52.9	43.5	43.7
5 years	15,651	46.9	59.4	51.0	48.1

Multiple imputation

We used Multiple imputation by chained equations[8] to handle both missing values in analysis covariates and missingness in the continuous outcomes,[9, 10] which will generate five imputed datasets.

Predictive mean matching with 10 donors[11] was used to impute all categorical and continuous partially-observed variables to improve robustness to misspecification of the imputation model. We assumed that data were `missing at random' (MAR). For missing values in the continuous outcome measures, this assumption implies that this missingness is at random, i.e. at random conditional on all other measures in the model including all preceding and subsequent levels of the measure in question, and the levels of any measures that were available at the timepoint in question. Some measurements taken repeatedly over time, e.g., HbA1c and BMI, were missing at baseline for some individuals, and the same rationale for supporting the underlying MAR assumption would apply here as for outcomes with intermittent missingness, given that measurements for time periods prior to baseline and during the subsequent follow-up periods were available for the imputation models.

For the analysis covariates, ethnicity had the greatest proportion of missing values. Previous literature has shown that conducting a MAR analysis for ethnicity can lead to similar point estimates as implementing missing data methods under the `missing not at random' assumption.[12, 13] Here, our base case analysis used multiple imputation for ethnicity, along with the other covariates, and we examined robustness to the assumed missing data mechanism by undertaking complete-case analysis in a sensitivity analysis.

Imputation model specification

Due to the non-linear trajectory of the continuous outcomes, all continuous measures from 6 months to 5 years were used when imputing a continuous outcome at time *t.* For example, a patient's observed HbA1c values from baseline, 6 months and 2-5 years would be used to impute their unobserved year 1 HbA1c value, in addition to any auxiliary information which would improve the imputed value. The imputation models for the analysis covariates included information on both the survival and continuous outcomes to ensure congeniality[14] between the covariates and each continuous and survival outcome. The imputation models for all partially-observed covariates are specified in **Supplementary methods table 3**. Interactions included in the analysis models were treated as `just another variable'[15] and imputed using MICE with PMM.

Supplementary methods table S3: Fitted imputation models for each partially-observed variable used in the continuous and survival analyses.

**Partially-observed**	**Covariates adjusted for in each imputation model***
Covariates	
Ethnicity	Analysis Covariates<br>All Nelson-Aalen Estimates & Event indicators<br>All continuous outcomes for t=0.5, 1, …, 5yrs
Index of multiple deprivation	
Baseline Hba1c	
Baseline eGFR	
BMI	
Smoking status	
Alcohol status	
Systolic blood pressure	
Diastolic blood pressure	
Baseline Hba1c squared	
Baseline Hba1c * Age at baseline	
Baseline Hba1c * BMI	
Baseline Hba1c * Sex	
Continuous clinical outcomes	
Hba1c at time *t*	Analysis Covariates<br>All Continuous outcomes for t=0.5, 1, …, 5yrs
eGFR at time *t*	
BMI at time *t*	
Systolic blood pressure at time *t*	
*For patients who died, their imputation models also included time to death from baseline.	

Analysis covariates, Survival information and Continuous outcomes are specified in Supplementary methods table 1.

Imputation model stratification and follow-up status

The imputation models were stratified by (i) treatment assignment (SGLT2i, DPP4i, SU), and (ii) status across the follow-up period ((i) a patient has died during follow-up; (ii) a patient is fully followed for 5 years, and (iii) censored as a patient no longer contributes to the study due to either reaching the end of the study monitoring period, or the patient/practice no longer contributing to CPRD). For patients who are censored, we assume that this is `completely at random' as censoring pertains to administrative reasons or due to the end of the follow-up period, which are unlikely to be related to the patient's

characteristics of interest in our analyses, such as their prognosis. For people who have died, their corresponding missing values prior to death are likely to differ to those who were alive at a given follow-up timepoint, and hence missing values for these people were imputed separately from those with full follow-up, or who stopped contributing before full follow-up was reached.

In total, 9 imputation models were used to impute each partially-observed variable depending on which treatment and follow-up status strata they belonged to. The three imputation models for patients who died (Death-SU; Death-SGLT2i; Death-DPP4i) also included a "time to death from baseline", in addition to the variables specified in **Supplementary methods table 3**, to recognise that this may be predictive of the missing outcome.

Due to the specification of the imputation models (**Supplementary methods table 3**) and the MICE[16] package in R, it was not possible to restrict imputing missing continuous outcome values up to the point of death or the point of no longer contributing to the study. Instead, missing values in the continuous clinical measures were imputed for all timepoints t=0.5, 1,…, 5 years. Censoring rules were then applied post-imputation before running the statistical analyses.

Post-imputation estimation of treatment effects and confidence intervals

The relative treatment effects were estimated in each imputed dataset using two-stage residual inclusion IV (with a frailty inclusion for time-to-event outcomes when using Cox proportional hazards).[3] Rubin's rules[17] was applied to obtain an overall treatment effect:

$$\hat{\hat{\theta}}_d = M^{-1} \sum_{m=1}^{M} \hat{\theta}_{m,d}$$

where *d* = (i) SGLT2i vs. SU, (ii) SGLT2i vs. DPP4i or (iii) DPP4i vs. SU. For Cox proportional hazards, Rubin's rules were applied on the log-hazard scale. The analysis model of interest was applied to each of the five multiply imputed datasets (M=5). This number of imputations was chosen as the overall analytical framework (IV residual inclusion) required that standard errors were estimated with the non-parametric bootstrap i.e. each of the nine imputation models were applied within each of the 500 bootstrap replications. The choice of M=5[18, 19] was a balance between recognising the importance of the number of imputed datasets for improved inference and the impact on computational time when running MI, non-parametric bootstrapping, IV residual inclusion and a Cox proportional hazards model with a frailty inclusion term.

Confidence intervals for the treatment effects were estimated using bootstrap sampling (BS), stratifying by region, treatment group, death and censoring status to maintain similar sampling patterns within each bootstrap sample. The original unimputed data were bootstrapped 500 times, and within each bootstrap sample, MI was applied (BS-then-MI).[20, 21]

Within each bootstrap sample $b = 1, \dots, 500$, we took the same approach to handling missing data and implementing the analysis model, as previously specified. Rubin's rules were applied to the *M* imputed datasets of bootstrap sample *b* to get an overall treatment effect for each drug comparison *d*:

$$\hat{\hat{\theta}}_{b,d} = M^{-1} \sum_{m=1}^{M} \hat{\theta}_{m,d}$$

The 500 estimates of $\hat{\hat{\theta}}_{b,d}$ was used to estimate variance and calculate *t*-based confidence intervals.

SUPPLEMENTARY TABLES

Supplementary table 1: Summary of target trial emulation

	Target trial	Emulation
**Eligibility criteria**	Inclusion criteria:<br>• People aged ≥18 years with a T2DM diagnosis.<br>• Initiate first-line oral antidiabetic treatment with metformin monotherapy.<br>• Initiate second-line oral antidiabetic treatment with one of SU, DPP4i or SGLT2i added on to metformin.<br><br>Exclusion criteria:<br>• Women with a record of pregnancy within 12 months prior to second-line treatment initiation.<br>• People whose last recorded eGFR<30mL/min/1.73m^2.<br>• People whose primary care data cannot be linked to secondary care data (essential for outcome definitions).	Inclusion criteria:<br>• People aged ≥18 years with a T2DM diagnosis.<br>• Initiate first-line oral antidiabetic treatment with metformin monotherapy.<br>• Initiate second-line oral antidiabetic treatment with one of SU, DPP4i or SGLT2i added on to metformin.<br>• At least one metformin prescription within 60 days prior to second-line treatment initiation.<br>• At least one metformin prescription on the same day or within 60 days post-second-line treatment initiation.<br><br>Exclusion criteria:<br>• Women with a record of pregnancy within 12 months prior to second-line treatment initiation.<br>• People whose last recorded eGFR<30mL/min/1.73m^2.<br>• People whose primary care data cannot be linked to secondary care data (essential for outcome definitions).
**Treatment assignment**	Participants randomly assigned to add one of SU, DPP4i, or SGLT2i to metformin monotherapy.	We used the tendency to prescribe DPP4i or SGLT2i versus SU as the instrumental variable for receipt of these alternative second-line oral antidiabetic treatments. The instrumental variable analysis aimed to reduce the risk of confounding (thus mimicking randomisation in the target

	Target trial	Emulation
		trial) (see details in Main Text (Methods pages 14-15, Supplementary figures 1A-B).
**Treatment initiation**	Initiation of one of SU, DPP4i or SGLT2i, all added to metformin, at randomisation.	We used GP prescriptions from the CPRD for one of SU, DPP4i, or SGLT2i, added to metformin monotherapy. The day of first prescription for SU, DPP4i, or SGLT2i served as the index date. All participants must also have had a prescription for metformin on the same day or within 60 days post index date to ensure that participants are adding on to metformin monotherapy rather than stopping metformin when switching to SU or DPP4i, or SGLT2i.[22, 23]
**Treatment strategy**	The duration of second-line treatment, and then all subsequent treatments, including reversion to monotherapy, or further intensification with additional oral treatments of insulin was determined over follow-up.<br><br>Participants may change their treatment through the course of the study. Changes may be captured using additional GP prescribing data.	The duration of second-line treatment was extracted from prescription data.<br><br>Information was collected on whether participants changed their treatment during study follow-up, and the form of treatment and duration of any subsequent treatment during the follow-up period.<br><br>All continuous courses of treatment were defined using the duration field of the CPRD prescribing data. A grace period of 60 days was added to the end of each prescription to allow for delays in filling new prescriptions for a continuous course of treatment. (See also causal contrasts).
**Follow-up**	Follow-up starts at treatment initiation. Participants are followed until 31 December 2021. Death/outcome of interest are censoring events.	Follow-up started at treatment initiation. Participants were followed until the outcome date, or 31 December 2021 (continuous outcomes defined in primary care, e.g., HbA1c) or 31 March 2021 (time-to-event outcomes defined in primary or secondary care, e.g., MACE). Linked hospital data

	Target trial	Emulation
		were only available up to 31 March 2021. Death/outcome of interest are censoring events.
**Outcomes**	**Primary outcome:**<br>Change in HbA1c (mmol/mol) at 1 year follow-up.<br><br>**Secondary outcomes:**<br>Change in BMI, systolic blood pressure, eGFR at 0.5, 1, 2, 3, 4, 5 years follow-up, and change in HbA1c at 0.5, 2, 3, 4, 5 years follow-up.<br><br>40% decline in eGFR from baseline.<br><br>Major adverse kidney event (MAKE): composite of 40% decline in eGFR from baseline, end-stage kidney disease, or all-cause death.<br><br>Heart failure hospitalisation.<br><br>Major adverse cardiovascular event (MACE): stroke, myocardial infarction, or cardiovascular-specific death.<br><br>All-cause death.	**Primary outcome:**<br>Change in HbA1c (mmol/mol) at 1 year follow-up.<br><br>**Secondary outcomes:**<br>Change in BMI, systolic blood pressure, eGFR at 0.5, 1, 2, 3, 4, 5 years follow-up, and change in HbA1c at 0.5, 2, 3, 4, 5 years follow-up.<br><br>40% decline in eGFR from baseline.<br><br>Major adverse kidney event (MAKE): composite of 40% decline in eGFR from baseline, end-stage kidney disease, or all-cause death.<br><br>Heart failure hospitalisation.<br><br>Major adverse cardiovascular event (MACE): stroke, myocardial infarction, or cardiovascular-specific death.<br><br>All-cause death.
**Causal contrasts of interest**	Intention-to-treat<br>average treatment effect.	Intention-to-treat<br>average treatment effect.
**Analysis plan to estimate causal**	Multivariable survival analysis adjusting for any chance imbalances in the treatment groups. Average treatment effect estimated as change scores with 95% confidence intervals (mean change in outcome from baseline) for	Applied 2SRI model. In the first stage we estimated propensity score models to estimate probabilities that each person was prescribed each treatment based on their

	Target trial	Emulation
**contrasts of interest**	continuous outcomes and as hazard ratios with 95% confidence intervals for time-to-event outcomes.	baseline covariates and their clinical commissioning group's tendency to prescribe that treatment.<br><br>The second stage outcome models included the generalised residuals from the first stage models in an ordinary least squares (OLS) regression model (continuous outcomes) or Cox proportional hazards model (time-to-event outcomes) with an individual frailty. Models in both stages will include all measured baseline covariates, with additional polynomials and covariate interactions selected by Least Absolute Shrinkage and Selection Operator (LASSO) regression. The rationale for including contextual variables in the outcome regression model was to enable the IV approach to make more plausible assumptions (see text and supplement).<br><br>Alternative analyses included multivariable regression analysis, adjusting for measured confounders.<br><br>Post-hoc, we conducted an inverse probability of treatment (IPTW) analysis, and an IPTW-weighted regression (doubly robust), as a further alternative analysis.<br><br>Average treatment effects were reported as change scores (mean change in outcome from baseline) or hazard ratios with 95% confidence intervals.

Supplementary table 2: Details of inclusion and exclusion criteria for the study population

**Inclusion criteria**	**Exclusion criteria**
• Aged ≥18 years (the study is of adults only).<br>• T2DM diagnosis code, to avoid including people prescribed antidiabetic drugs for other indications (e.g., polycystic ovarian syndrome).<br>• Prescribed metformin monotherapy as first-line oral antidiabetic treatment, on the same day or following a T2DM diagnosis.<br>• Registered with GP in England with acceptable data standards flag by CPRD (to help ensure adequate data availability).<br>• Registered with GP for ≥1 year prior to first metformin prescription (to help ensure adequate baseline data availability and reduce recording of past events as incident).<br>• Initiate SU, DPP4i, or SGLT2i between 1 January 2015 to 31 December 2020 (the study period).<br>• At least 1 metformin prescription within 60 days prior to new second-line drug, and at least one metformin prescription on the same day or within 60 days after new second-line drug, to ensure the person is adding on to metformin and not switching.<br>• Linked to HES/ONS/IMD data (to help ensure outcomes are captured).	• Prescribed >1 non-metformin antidiabetic drugs on the date of second-line treatment initiation (beyond study scope).<br>• Initiates second-line oral antidiabetic treatment with drug class other than SU, DPP4i, or SGLT2i (beyond study scope).<br>• Latest eGFR recorded by the GP is <30mL/min/1.73m^2 (since at the time of data-collection most GPs would not have prescribed metformin for people with eGFR <30ml/min; the results from the DAPA-CKD trial (which did randomise people with eGFR less than 30ml/min/1.73m^2) were only available towards the very end of the study period and are unlikely to have informed decisions taken in primary care).<br>• Women who have a record of pregnancy in primary care within 1 year prior to second-line antidiabetic treatment initiation (since guidelines are different for this group).

Supplementary table 3: Details of covariate data sources and definitions

Covariable	Data source	Details
Age	CPRD	CPRD<br>Age at baseline derived using the year of birth
Sex	CPRD	CPRD<br>Sex recorded in CPRD
Ethnicity	CPRD, HES	CPRD<br>Clinical code (Read or Snomed) indicating ethnicity, further categorised into four categories (White, South Asian, Black, Mixed/Other)<br><br>HES<br>Demographic data entered at in-patient hospitalisation, further categorised into four categories (White, South Asian, Black, Mixed/Other)<br><br>Where CPRD ethnicity is missing, HES ethnicity is used to define people's ethnicity. Where ethnicities disagree, that recorded in CPRD is used.
Deprivation quintile	Index of Multiple Deprivation (IMD)	IMD<br>Deprivation quintile based on a composite index assigned to each Lower layer Super Output Area or neighbourhood (small area level) in England, assigned to each patient based on residence.
Time since type 2 diabetes diagnosis	CPRD	CPRD<br>Days between the first diagnosis code (Read or Snomed) for T2DM and baseline
Time on first-line (metformin monotherapy)	CPRD	CPRD<br>Days between the first prescription for metformin and baseline
GP size	CPRD	CPRD<br>Number of patients actively registered with the GP to which the patient belongs, derived using the CPRD denominator file, uses 2014 figures

Covariable	Data source	Details
NHS Region	CPRD	CPRD<br>The region in which the GP practice is located to which each patient is registered. Regions include: East of England, London, Midlands, North East and Yorkshire, North West, South East, and South West
Co-prescriptions prescribed within 60 days of baseline (including RASi and statins)	CPRD	CPRD<br>At least one prescription for the drug class of interest in the prescription history in the primary care record, within 60 days of baseline.
Comorbidities at baseline defined in primary and secondary care (including previous MI, unstable angina, stroke, hypoglycaemia, CHF)	CPRD and HES	CPRD<br>Diagnosis code (Read or Snomed) for each comorbidity prior to or the same day as baseline<br><br>HES<br>Diagnosis code (ICD-10) for each comorbidity prior to or the same day as baseline in any diagnostic position of any episode of a spell
Comorbidities at baseline defined in primary care (cancer (any), advanced eye disease, lower extremity amputation, proteinuria)	CPRD	CPRD<br>Diagnosis code (Read or Snomed) for comorbidity prior to or the same day as baseline
HbA1c	CPRD	CPRD<br>Laboratory test recording the most recent HbA1c recorded within 180 days prior to baseline. We chose this time window because NICE guidance recommends HbA1c be measured at least every 6-months.[24] Units reported as mmol/mol (tests recording HbA1c in % will be converted to mmol/mol).
eGFR and eGFR/CKD status	CPRD	CPRD<br>Using the eGFR derived from serum creatinine using the CKD-EPI equation without adjustment for ethnicity recorded within 540 days prior to baseline, we will group people as either having eGFR≥60mL/min/1.73m^2 or eGFR<60mL/min/1.73m^2 (indicating impaired kidney function). We chose the

Covariable	Data source	Details
		540 day window since the Quality Outcomes Framework (Pay for Performance for England) recommend that patients with T2DM have a full clinical review annually, with an additional half-year (180 days) added to account for delays in arranging appointments and data entry.[23]
SBP and DBP	CPRD	CPRD<br>Clinical measures captured in CPRD within 540 days prior to baseline. The 540 day window was used for the same reasons outlined as for eGFR (see above).
BMI	CPRD	CPRD<br>BMI derived from weight and height measures entered by the GP (preferred), or BMI entered directly by the GP prior to the index date. An algorithm defined by Bhaskaran et al was used.[25, 26]
Smoking status	CPRD	CPRD<br>Clinical codes describing smoking status in the primary care record, using an algorithm previously defined in CPRD data
Alcohol status	CPRD	CPRD<br>Clinical codes describing alcohol intake in the primary care record, using an algorithm previously defined in CPRD data
In-patient hospitalisation (any reason) in the past year	HES	HES<br>At least one spell (hospitalisation) recorded in the patient's secondary care record (HES admitted patient care record) in the year prior to baseline

Supplementary table 4: Details of outcome data sources and definitions

Type of outcome	Outcome	Data source	Details
Continuous	Absolute change in HbA1c	CPRD	CPRD<br>Laboratory measures of HbA1c
Continuous	Absolute change in eGFR	CPRD	CPRD<br>Laboratory measures of serum creatinine, converted to eGFR using the 2009 CKD-EPI equation without adjustment for ethnicity
Continuous	Absolute change in BMI	CPRD	CPRD<br>Measures of body weight and height, using a previously developed algorithm to define BMI in CPRD data[26]
Continuous	Absolute change in SBP	CPRD	CPRD<br>Measures of systolic blood pressure
Time-to-event	MACE,<br>including MI, stroke, and CVD-specific mortality	CPRD, HES, ONS	CPRD<br>Diagnosis codes for MI, stroke<br><br>HES<br>Diagnosis codes for MI, stroke in the first or second diagnostic position of any episode in a spell)<br><br>ONS<br>Death date and CVD-specific ICD-10 code as main cause of death (any ICD-10 code with ‘I’ as the first digit (e.g., I00-I99).
Time-to-event	MI	CPRD, HES	CPRD<br>Diagnosis code for MI<br><br>HES<br>Diagnosis code for MI in the first or second diagnostic position of any episode in a spell

Type of outcome	Outcome	Data source	Details
Time-to-event	Stroke	CPRD, HES	CPRD<br>Diagnosis code for stroke<br><br>HES<br>Diagnosis code for stroke in the first or second episode of any episode in a spell
Time-to-event	All-cause mortality	ONS	ONS<br>Death date
Time-to-event	Heart failure hospitalisation	HES	HES<br>Diagnosis code for HF in the first or second diagnostic position of any episode in a spell
Time-to-event	Major adverse kidney event (MAKE), a composite outcome including 40% decline in eGFR, ESKD, and all-cause mortality	CPRD, ONS	CPRD<br>40% decline in eGFR at baseline (using eGFR derived from laboratory measures of serum creatinine)<br>ESKD (clinical codes diagnosing ESKD/chronic dialysis/kidney transplant)<br><br>ONS<br>Death date
Time-to-event	40% decline in eGFR from baseline, which could be a proxy for the rarer ESKD outcome[27]	CPRD	CPRD<br>Laboratory measures for serum creatinine to derive eGFR using the 2009 CKD-EPI equation without correction for ethnicity
Time-to-event	ESKD		CPRD<br>Clinical codes for diagnosis of ESKD, or dialysis/kidney transplant codes

Supplementary table 5: Frequency of individual drug substances within each drug class included as the treatments of interest in this study

Drug class	Drug substance name	n	%
SU	Glibenclamide	24	0.0
	Gliclazide	24,768	32.7
	Glimepiride	807	1.1
	Glipizide	80	0.1
	Tolbutamide	14	0.0
DPP4i	alogliptin benzoate	10,051	13.3
	Linagliptin	8,063	10.6
	Saxagliptin	1,296	1.7
	Sitagliptin	14,915	19.7
	Vildagliptin	139	0.2
SGLT2i	Canagliflozin	2,606	3.4
	Dapagliflozin	6,207	8.2
	Empagliflozin	6,732	8.9
	Ertugliflozin	37	0.0
Total		75,739	100.0

Supplementary table 6: Time spent on second-line antidiabetic treatment, overall and stratified by treatment group

Second-line treatment prescribed	People prescribed each 2nd line treatment, n (col %)	Median days on continuous 2nd line antidiabetic treatment (IQR) during the first 2 years of follow-up	Median days on continuous 2nd line antidiabetic treatment (IQR) during complete follow-up	People who initiate 3rd line treatment within 2 years of 2nd line treatment initiation, n (col %)	3rd line treatment prescribed*	People prescribed each 3rd line treatment, n (col %)	Median days on continuous 3rd line antidiabetic treatment during complete follow-up
**Overall**	75,739 (100)	307 (83-730)	307 (83-748)	41,040 (100)	-	63,872	83 (30-264)
**Metformin + SU**	25,693 (34)	248 (67-671)	248 (67-671)	15,107 (59)	**Metformin monotherapy**	10,100 (45)	111 (61-359)
					**SU monotherapy**	4,983 (22)	66 (27-94)
					**Triple therapy**	5,669 (25)	112 (27-416)
					**Other**	1,875 (8)	81 (63-125)
**Metformin + DPP4i**	34,464 (45)	345 (96-730)	345 (96-801)	17,749 (52)	**Metformin monotherapy**	9,599 (34)	97 (41-320)
					**DPP4i monotherapy**	6,915 (24)	66 (27-92)
					**Triple therapy**	9,052 (32)	139 (27-489)
					**Other**	3,028 (11)	93 (62-247)
**Metformin + SGLT2i**	15,582 (21)	328 (84-730)	328 (84-743)	8,184 (53)	**Metformin monotherapy**	5,084 (40)	90 (42-265)
					**SGLT2i monotherapy**	3,357 (27)	62 (27-86)
					**Triple therapy**	2,755 (22)	124 (27-413)
					**Other**	1,455 (11)	90 (64-263)

*Other types of 3rd line treatment include monotherapies with insulin, thiazolidinediones, glucagon-like peptide 1 receptor agonists (GLP1RA), and no treatment (de-prescribed).

Supplementary table 7: Describing the proportion of the study population who initiate second-line oral antidiabetic treatment during the COVID-19 pandemic, and corresponding missingness in the primary outcome by treatment group

	**Initiate second-line oral antidiabetic treatment during COVID-19 period**		**Missingness in HbA1c at 1-year (primary outcome) pre-COVID-19[1]**		**Missingness in HbA1c at 1-year (primary outcome) during COVID-19[2]**	
**Second-line antidiabetic treatment**	**n**	**% (row) of total study population**	**n missing**	**% (row) missing of total study population**	**n missing**	**% (row) missing of total study population**
**Total**	7,553	17.5	24,448	35.9	3,544	46.9
**MET-SU**	1,835	7.1	8,613	36.1	869	47.4
**MET-DPP4i**	2,996	8.7	10,808	34.4	1,363	45.5
**MET-SGLT2i**	2,722	17.5	5,027	39.1	1,312	48.2

[1]Pre-COVID-19: prior to 23 March 2020 (the date of the first UK lockdown)

[2]COVID-19: 23 March 2020 to the end of the study follow-up for continuous outcomes (31 December 2021)

Supplementary table 8: Differences in the change in continuous clinical measures for the three second-line antidiabetic treatment comparisons for the main analysis (2SRI, bootstrap-multiple imputation)

			**Year of follow-up**					
**Outcome**	**Comparison**		**0.5**	**1**	**2**	**3**	**4**	**5**
**Difference in the change in HbA1c (mmol/mol) from baseline**		**N =**	72,066	66,702	52,962	39,099	26,366	15,651
	**DPP4i vs SU**	**Estimate (95% CI)**	2.89 (1.99, 3.80)	0.68 (-0.31, 1.68)	-1.40 (-2.55, -0.24)	-1.84 (-3.21, -0.47)	-2.33 (-3.94, -0.72)	-2.78 (-5.10, -0.45)
	**SGLT2i vs SU**	**Estimate (95% CI)**	2.11 (1.09, 3.12)	-2.51 (-3.72, -1.30)	-4.95 (-6.46, -3.45)	-6.50 (-8.47, -4.52)	-5.35 (-7.98, -2.73)	-1.77 (-6.89, 3.35)
	**SGLT2i vs DPP4i**	**Estimate (95% CI)**	-0.79 (-1.93, 0.35)	-3.20 (-4.58, -1.81)	-3.56 (-5.28, -1.84)	-4.66 (-6.90, -2.41)	-3.02 (-5.95, -0.09)	1.01 (-4.75, 6.76)
**Difference in the change in BMI (kg/m²) from baseline**		**N =**	72,066	66,702	52,962	39,099	26,366	15,651
	**DPP4i vs SU**	**Estimate (95% CI)**	-0.52 (-0.68, -0.37)	-0.70 (-0.83, -0.56)	-0.64 (-0.80, -0.48)	-0.57 (-0.78, -0.35)	-0.79 (-1.05, -0.53)	-0.68 (-1.09, -0.28)
	**SGLT2i vs SU**	**Estimate (95% CI)**	-1.41 (-1.57, -1.25)	-1.55 (-1.72, -1.37)	-1.50 (-1.74, -1.23)	-1.55 (-1.92, -1.19)	-1.32 (-1.81, -0.83)	-1.83 (-2.80, -0.85)
	**SGLT2i vs DPP4i**	**Estimate (95% CI)**	-0.89 (-1.06, -0.71)	-0.85 (-1.03, -0.66)	-0.85 (-1.12, -0.58)	-0.99 (-1.39, -0.59)	-0.52 (-1.07, 0.02)	-1.14 (-2.20, -0.09)
**Difference in the change in eGFR (mL/min/1.73m²) from baseline**		**N =**	72,066	66,702	52,962	39,099	26,366	15,651
	**DPP4i vs SU**	**Estimate (95% CI)**	-0.14 (-0.71, 0.44)	0.14 (-0.46, 0.73)	1.44 (0.69, 2.19)	2.85 (1.97, 3.74)	3.40 (2.26, 4.55)	4.01 (2.42, 5.61)
	**SGLT2i vs SU**	**Estimate (95% CI)**	-0.21 (-0.87, 0.46)	0.44 (-0.29, 1.18)	1.39 (0.49, 2.30)	1.99 (0.69, 3.28)	3.66 (1.97, 5.36)	5.99 (2.83, 9.15)
	**SGLT2i vs DPP4i**	**Estimate (95% CI)**	-0.07 (-0.87, 0.69)	0.31 (-0.53, 1.14)	-0.04 (-1.09, 1.01)	-0.87 (-2.35, 0.61)	0.26 (-1.81, 2.33)	1.98 (-1.56, 5.51)

			Year of follow-up					
**Outcome**	**Comparison**		**0.5**	**1**	**2**	**3**	**4**	**5**
**Difference in the change in SBP (mm Hg) from baseline**		**N =**	72,066	66,702	52,962	39,099	26,366	15,651
	**DPP4i vs SU**	**Estimate (95% CI)**	-0.80 (-1.70, 0.09)	-0.31 (-1.29, 0.66)	-0.43 (-1.47, 0.62)	-0.94 (-2.13, 0.24)	-1.28 (-2.70, 0.13)	-0.55 (-2.76, 1.66)
	**SGLT2i vs SU**	**Estimate (95% CI)**	-2.57 (-3.60, -1.54)	-2.07 (-3.10, -1.04)	-2.97 (-4.31, -1.62)	-3.11 (-4.83, -1.40)	-0.96 (-3.19, 1.26)	-5.64 (-9.73, -1.56)
	**SGLT2i vs DPP4i**	**Estimate (95% CI)**	-1.77 (-2.91, -0.62)	-1.76 (-2.99, -0.53)	-2.54 (-4.05, -1.03)	-2.17 (-4.24, -0.11)	0.32 (-2.27, 2.91)	-5.09 (-9.82, -0.36)

Supplementary table 9: Crude event counts and rates (95% CI) for time-to-event kidney and cardiovascular outcomes up to 2-years follow-up

Outcome	Exposure	No. of events	Person-time	Rate per 1000 PY	95% CI (lower)	95% CI (upper)
**≥40% decline in eGFR**	**Overall**	1252	125.47	9.98	9.44	10.55
	**SU**	500	43.64	11.46	10.50	12.51
	**DPP4i**	618	58.07	10.64	9.84	11.52
	**SGLT2i**	134	23.76	5.64	4.76	6.68
**ESKD**	**Overall**	66	126.39	0.52	0.41	0.66
	**SU**	22	44.02	0.50	0.33	0.76
	**DPP4i**	38	58.50	0.65	0.47	0.89
	**SGLT2i**	6	23.86	0.25	0.11	0.56
**MAKE**	**Overall**	3187	125.44	25.41	24.54	26.30
	**SU**	1487	43.63	34.08	32.39	35.86
	**DPP4i**	1433	58.05	24.69	23.44	26.00
	**SGLT2i**	267	23.76	11.24	9.97	12.67
**Heart failure hospitalisation**	**Overall**	821	125.78	6.53	6.10	6.99
	**SU**	360	43.75	8.23	7.42	9.12
	**DPP4i**	398	58.22	6.84	6.20	7.54
	**SGLT2i**	63	23.82	2.64	2.07	3.39
**MACE**	**Overall**	2172	124.71	17.42	16.70	18.16
	**SU**	896	43.33	20.68	19.37	22.08
	**DPP4i**	989	57.74	17.13	16.09	18.23
	**SGLT2i**	287	23.63	12.14	10.82	13.63
**All-cause death**	**Overall**	2043	126.43	16.16	15.47	16.88
	**SU**	1039	44.04	23.59	22.20	25.07
	**DPP4i**	864	58.53	14.76	13.81	15.78
	**SGLT2i**	140	23.86	5.87	4.97	6.92

Supplementary table 10: Summary of results from main analysis for kidney, cardiovascular, and mortality time-to-event outcomes, as well as summary of results for alternative analyses for kidney, cardiovascular, and mortality outcomes

Outcome	Treatment comparison	Analysis method*	Hazard ratio	95% CI (lower)	95% CI (upper)
**≥40% decline in eGFR**	DPP4i vs SU	Base (2-years follow-up max)	0.66	0.37	1.17
		Complete case (CC)	0.78	0.39	1.58
		Multivariable regression – CC	0.98	0.85	1.12
		Base (5-years follow-up max)	0.65	0.42	0.99
	SGLT2i vs SU	Base (2-years follow-up max)	0.42	0.22	0.81
		Complete case (CC)	0.40	0.17	0.91
		Multivariable regression – CC	0.78	0.61	0.99
		Base (5-years follow-up max)	0.47	0.24	0.92
	SGLT2i vs DPP4i	Base (2-years follow-up max)	0.64	0.29	1.43
		Complete case (CC)	0.51	0.18	1.39
		Multivariable regression – CC	0.80	0.63	1.01
		Base (5-years follow-up max)	0.73	0.33	1.59
**MAKE**	DPP4i vs SU	Base (2-years follow-up max)	0.72	0.50	1.03
		Complete case (CC)	0.86	0.56	1.32
		Multivariable regression – CC	0.74	0.68	0.80
		Base (5-years follow-up max)**	-	-	-
	SGLT2i vs SU	Base (2-years follow-up max)	0.79	0.51	1.23
		Complete case (CC)	0.81	0.48	1.39
		Multivariable regression – CC	0.59	0.51	0.68
		Base (5-years follow-up max)**	-	-	-
	SGLT2i vs DPP4i	Base (2-years follow-up max)	1.11	0.66	1.84
		Complete case (CC)	0.94	0.50	1.79
		Multivariable regression – CC	0.80	0.69	0.92
		Base (5-years follow-up max)**	-	-	-
**Heart failure hospitalisation**	DPP4i vs SU	Base (2-years follow-up max)	1.41	0.73	2.71
		Complete case (CC)	1.26	0.63	0.63
		Multivariable regression – CC	0.74	0.63	0.87

Outcome	Treatment comparison	Analysis method*	Hazard ratio	95% CI (lower)	95% CI (upper)
		Base (5-years follow-up max)	1.25	0.74	2.11
	SGLT2i vs SU	Base (2-years follow-up max)	0.46	0.20	1.05
		Complete case (CC)	0.43	0.16	1.11
		Multivariable regression – CC	0.50	0.37	0.69
		Base (5-years follow-up max)	0.63	0.28	1.42
	SGLT2i vs DPP4i	Base (2-years follow-up max)	0.32	0.12	0.85
		Complete case (CC)	0.34	0.11	1.06
		Multivariable regression – CC	0.68	0.50	0.93
		Base (5-years follow-up max)	0.51	0.20	1.30
**MACE**	DPP4i vs SU	Base (2-years follow-up max)	1.09	0.70	1.69
		Complete case (CC)	1.02	0.64	1.63
		Multivariable regression – CC	0.82	0.74	0.92
		Base (5-years follow-up max)	0.90	0.63	1.27
	SGLT2i vs SU	Base (2-years follow-up max)	0.99	0.61	1.62
		Complete case (CC)	0.93	0.53	1.63
		Multivariable regression – CC	0.83	0.71	0.96
		Base (5-years follow-up max)	1.12	0.70	1.80
	SGLT2i vs DPP4i	Base (2-years follow-up max)	0.91	0.51	1.63
		Complete case (CC)	0.91	0.48	1.72
		Multivariable regression – CC	1.00	0.87	1.16
		Base (5-years follow-up max)	1.25	0.72	2.16
**All-cause mortality**	DPP4i vs SU	Base (2-years follow-up max)	0.82	0.51	1.32
		Complete case (CC)	0.93	0.55	1.58
		Multivariable regression – CC	0.63	0.56	0.70
		Base (5-years follow-up max)	0.90	0.63	1.29
	SGLT2i vs SU	Base (2-years follow-up max)	1.14	0.64	2.03
		Complete case (CC)	1.17	0.61	2.27
		Multivariable regression – CC	0.50	0.41	0.62
		Base (5-years follow-up max)	1.25	0.77	2.05
		Base (2-years follow-up max)	1.39	0.71	2.74

Outcome	Treatment comparison	Analysis method*	Hazard ratio	95% CI (lower)	95% CI (upper)
	SGLT2i vs DPP4i	Complete case (CC)	1.26	0.59	2.68
		Multivariable regression – CC	0.80	0.66	0.98
		Base (5-years follow-up max)	1.39	0.77	2.50
**Myocardial infarction**	DPP4i vs SU	Base (2-years follow-up max)	1.41	0.74	2.68
		Complete case (CC)	1.19	0.60	2.35
		Multivariable regression - CC	0.83	0.70	0.98
		Base (5-years follow-up max)	0.87	0.52	1.46
	SGLT2i vs SU	Base (2-years follow-up max)	1.35	0.67	2.71
		Complete case (CC)	1.18	0.53	2.61
		Multivariable regression - CC	0.92	0.75	1.13
		Base (5-years follow-up max)	1.82	0.92	3.59
	SGLT2i vs DPP4i	Base (2-years follow-up max)	0.95	0.38	2.40
		Complete case (CC)	0.99	0.37	2.64
		Multivariable regression - CC	1.11	0.90	1.36
		Base (5-years follow-up max)	2.09	0.92	4.75
**Stroke**	DPP4i vs SU	Base (2-years follow-up max)	1.26	0.62	2.56
		Complete case (CC)	1.26	0.63	2.54
		Multivariable regression - CC	0.82	0.70	0.97
		Base (5-years follow-up max)	0.92	0.54	1.57
	SGLT2i vs SU	Base (2-years follow-up max)	0.63	0.30	1.31
		Complete case (CC)	0.73	0.32	1.69
		Multivariable regression - CC	0.74	0.58	0.96
		Base (5-years follow-up max)	0.65	0.30	1.39
	SGLT2i vs DPP4i	Base (2-years follow-up max)	0.50	0.21	1.21
		Complete case (CC)	0.58	0.22	1.51
		Multivariable regression - CC	0.90	0.70	1.16
		Base (5-years follow-up max)	0.71	0.30	1.64

***** Base method is the main analysis (2 stage-residual inclusion (2SRI) instrumental variable analysis with multiple imputation to account for missing data, assuming data are missing at random)

** Models could not converge for MAKE outcome extended to 5-years follow-up

Supplementary table 11: Differences in the change in continuous clinical measures for the three second-line antidiabetic treatment comparisons according to CVD status prior to initiation of second-line treatment (2SRI, bootstrap-multiple imputation)

			Year of follow-up					
			Six months		Year 1		Year 2	
Outcome	Comparison		CVD	No CVD	CVD	NO CVD	CVD	NO CVD
**Difference in the change in HbA1c (mmol/mol) from baseline**		N =	15,564	56,502	14,296	52,406	11,191	41,771
	DPP4i vs SU	Estimate	2.80	2.90	0.24	0.78	-1.53	-1.37
		(95% CI)	(1.77, 3.84)	(1.97, 3.84)	(-0.96, 1.44)	(-0.20, 1.76)	(-2.88, -0.18)	(-2.57, -0.18)
	SGLT2i vs SU	Estimate	2.90	1.89	-1.69	-2.70	-5.13	-4.88
		(95% CI)	(1.66, 4.15)	(0.85, 2.94)	(-3.21, -0.16)	(-3.90, -1.50)	(-6.89, -3.36)	(-6.32, -3.44)
	SGLT2i vs DPP4i	Estimate	0.10	-1.01	-1.93	-3.48	-3.59	-3.51
		(95% CI)	(-1.23, 1.44)	(-2.18, 0.16)	(-3.51, -0.34)	(-4.81, -2.15)	(-5.61, -1.57)	(-5.20, -1.81)
**Difference in the change in BMI (kg/m²) from baseline**		N =	15,564	56,502	14,296	52,406	11,191	41,771
	DPP4i vs SU	Estimate	-0.42	-0.55	-0.63	-0.72	-0.46	-0.70
		(95% CI)	(-0.62, -0.22)	(-0.71, -0.40)	(-0.79, -0.48)	(-0.86, -0.58)	(-0.65, -0.28)	(-0.86, -0.53)
	SGLT2i vs SU	Estimate	-1.29	-1.44	-1.51	-1.55	-1.48	-1.50
		(95% CI)	(-1.49, -1.10)	(-1.60, -1.28)	(-1.73, -1.29)	(-1.73, -1.37)	(-1.77, -1.18)	(-1.74, -1.26)
	SGLT2i vs DPP4i	Estimate	-0.87	-0.88	-0.88	-0.83	-1.01	-0.80
		(95% CI)	(-1.10, -0.65)	(-1.06, -0.70)	(-1.09, -.066)	(-1.01, -0.64)	(-1.33, -0.70)	(-1.07, -0.54)
**Difference in the change in eGFR (mL/min/1.73m²) from baseline**		N =	15,564	56,502	14,296	52,406	11,191	41,771
	DPP4i vs SU	Estimate	-0.31	-0.07	-0.22	0.25	1.51	1.42
		(95% CI)	(-0.96, 0.34)	(-.66, 0.52)	(-0.93, 0.49)	(-0.35, 0.84)	(0.65, 2.36)	(0.68, 2.15)
	SGLT2i vs SU	Estimate	0.26	-0.32	0.55	0.41	2.17	1.19
		(95% CI)	(-0.58, 1.09)	(-1.02, 0.38)	(-0.39, 1.49)	(-0.32, 1.13)	(1.03, 3.32)	(0.27, 2.10)
	SGLT2i vs DPP4i	Estimate	0.57	-0.25	0.77	0.16	0.67	-0.23
		(95% CI)	(-0.28, 1.42)	(-1.03, 0.53)	(-0.20, 1.75)	(-0.67, .98)	(-0.61, 1.94)	(-1.27, 0.80)

			Year of follow-up					
			Six months		Year 1		Year 2	
Outcome	Comparison		CVD	No CVD	CVD	NO CVD	CVD	NO CVD
Difference in the change in SBP (mm Hg) from baseline		N =	15,564	56,502	14,296	52,406	11,191	41,771
	DPP4i vs SU	Estimate (95% CI)	-1.18 (-2.21, -0.15)	-0.68 (-1.58, 0.22)	-0.88 (-1.98, 0.22)	-0.16 (-1.14, 0.83)	-0.32 (-1.51, 0.87)	-0.42 (-1.49, 0.64)
	SGLT2i vs SU	Estimate (95% CI)	-2.76 (-4.09, -1.43)	-2.55 (-3.59, -1.51)	-2.45 (-3.86, -1.05)	-2.03 (-3.07, -0.99)	-2.82 (-4.53, -1.11)	-3.00 (-4.33, -1.66)
	SGLT2i vs DPP4i	Estimate (95% CI)	-1.58 (-2.95, -0.21)	-1.87 (-2.96, -0.78)	-1.58 (-3.11, -0.04)	-1.88 (-3.09, -0.66)	-2.50 (-4.29, -0.71)	-2.57 (-4.09, -1.05)

Supplementary table 12: Summary of results from the subgroup analysis comparing kidney, cardiovascular, and mortality outcomes according to CVD status prior to initiation of second-line treatment

**Outcome**	**Treatment comparison**	**Cardiovascular disease (CVD) status**	**Hazard ratio**	**95% CI (lower)**	**95% CI (upper)**
**≥40% decline in eGFR**	DPP4i vs SU	No CVD	0.64	0.36	1.15
		CVD	0.69	0.36	1.32
	SGLT2i vs SU	No CVD	0.41	0.21	0.80
		CVD	0.48	0.19	1.18
	SGLT2i vs DPP4i	No CVD	0.64	0.28	1.44
		CVD	0.69	0.24	1.98
**MAKE**	DPP4i vs SU	No CVD	0.69	0.48	1.00
		CVD	0.75	0.50	1.14
	SGLT2i vs SU	No CVD	0.76	0.49	1.18
		CVD	0.95	0.54	1.66
	SGLT2i vs DPP4i	No CVD	1.09	0.65	1.83
		CVD	1.26	0.67	2.35
**Heart failure hospitalisation**	DPP4i vs SU	No CVD	1.57	0.79	3.09
		CVD	1.36	0.77	2.40
	SGLT2i vs SU	No CVD	0.43	0.18	1.02
		CVD	0.28	0.10	0.75
	SGLT2i vs DPP4i	No CVD	0.36	0.09	1.41
		CVD	1.15	0.73	1.82
**MACE**	DPP4i vs SU	No CVD	0.95	0.58	1.55
		CVD	1.09	0.61	1.97
	SGLT2i vs SU	No CVD	0.82	0.45	1.49
		CVD	0.77	0.48	1.26
	SGLT2i vs DPP4i	No CVD	0.88	0.50	1.54
		CVD	1.05	0.58	1.89

Outcome	Treatment comparison	Cardiovascular disease (CVD) status	Hazard ratio	95% CI (lower)	95% CI (upper)
**All-cause mortality**	DPP4i vs SU	No CVD	1.47	0.67	3.24
		CVD	1.36	0.68	2.7
	SGLT2i vs SU	No CVD	1.68	0.70	4.01
		CVD	1.38	0.71	2.68
	SGLT2i vs DPP4i	No CVD	1.46	0.73	2.9
		CVD	1.15	0.56	2.35

Supplementary table 13: Differences in the change in continuous clinical measures for the three second-line antidiabetic treatment comparisons for 2SRI analysis (complete cases)

			Year of follow-up					
**Outcome**	**Comparison**		**0.5**	**1**	**2**	**3**	**4**	**5**
**Difference in the change in HbA1c (mmol/mol) from baseline**		**N =**	46,900	42,441	32,364	23,082	15,126	8,022
	**DPP4i vs SU**	**Estimate**	2.64	0.39	-1.76	-3.04	-1.71	-2.25
		**(95% CI)**	(1.51, 3.77)	(-0.87, 1.65)	(-3.25, -0.26)	(-4.64, -1.45)	(-3.88, 0.45)	(-5.02, 0.53)
	**SGLT2i vs SU**	**Estimate**	2.02	-3.02	-6.11	-8.16	-7.51	-0.78
		**(95% CI)**	(0.79, 3.25)	(-4.54, -1.49)	(-8.14, -4.08)	(-10.85, -5.46)	(-11.05, -3.98)	(-6.11, 4.55)
	**SGLT2i vs DPP4i**	**Estimate**	-0.62	-3.41	-4.35	-5.11	-5.80	1.47
		**(95% CI)**	(-2.03, 0.79)	(-5.13, -1.69)	(-6.64, -2.06)	(-8.10, -2.12)	(-9.97, -1.63)	(-4.72, 7.66)
**Difference in the change in BMI (kg/m^2) from baseline**		**N =**	33,508	34,431	25,809	18,253	11,577	5,903
	**DPP4i vs SU**	**Estimate**	-0.53	-0.70	-0.78	-0.61	-1.02	-0.55
		**(95% CI)**	(-0.78, -0.28)	(-0.89, -0.51)	(-1.02, -0.54)	(-0.97, -0.26)	(-1.44, -0.60)	(-1.11, 0.01)
	**SGLT2i vs SU**	**Estimate**	-1.41	-1.57	-1.49	-1.74	-1.37	-1.77
		**(95% CI)**	(-1.62, -1.19)	(-1.83, -1.31)	(-1.83, -1.14)	(-2.27, -1.21)	(-2.12, -0.63)	(-3.06, -0.47)
	**SGLT2i vs DPP4i**	**Estimate**	-0.87	-0.87	-0.71	-1.13	-0.35	-1.22
		**(95% CI)**	(-1.16, -0.59)	(-1.14, -0.59)	(-1.10, -0.31)	(-1.73, -0.54)	(-1.20, 0.49)	(-2.68, 0.25)
**Difference in the change in eGFR (mL/min/1.73m^2) from baseline**		**N =**	39,113	39,337	30,034	21,398	14,060	7,659
	**DPP4i vs SU**	**Estimate**	-0.18	0.10	1.49	3.93	3.72	5.29
		**(95% CI)**	(-0.92, 0.55)	(-.72, 0.92)	(0.42, 2.55)	(2.74, 5.12)	(2.15, 5.28)	(3.00, 7.57)
	**SGLT2i vs SU**	**Estimate**	-0.28	0.73	1.99	2.36	4.69	6.98
		**(95% CI)**	(-1.17, 0.60)	(-0.24, 1.71)	(0.70, 3.28)	(0.27, 4.46)	(2.13, 7.26)	(2.98, 10.98)
	**SGLT2i vs DPP4i**	**Estimate**	-0.10	0.63	0.50	-1.57	0.98	1.69
		**(95% CI)**	(-1.06, 0.85)	(-0.45, 1.72)	(-1.06, 2.07)	(-3.96, 0.83)	(-2.00, 3.95)	(-3.05, 6.44)

Outcome	Comparison		Year of follow-up 0.5	1	2	3	4	5
Difference in the change in SBP (mm Hg) from baseline		N =	40,588	41,049	30,967	21,972	13,832	7,100
	DPP4i vs SU	Estimate	-0.78	0.08	-1.23	-1.05	-1.08	1.78
		(95% CI)	(-2.06, 0.50)	(-1.22, 1.38)	(-2.72, 0.26)	(-2.76, 0.65)	(-3.09, 0.93)	(-1.14, 4.70)
	SGLT2i vs SU	Estimate	-3.15	-1.94	-2.97	-4.04	-1.23	-4.57
		(95% CI)	(-4.58, -1.72)	(-3.40, -0.48)	(-4.77, -1.17)	(-6.33, -1.75)	(-4.49, 2.03)	(-9.62, 0.48)
	SGLT2i vs DPP4i	Estimate	-2.37	-2.01	-1.74	-2.99	-0.15	-6.35
		(95% CI)	(-4.03, -0.71)	(-3.77, -0.26)	(-3.93, 0.45)	(-5.75, -0.22)	(-3.89, 3.59)	(-12.44,-0.26)

Supplementary table 14: Differences in the change in continuous clinical measures for the three second-line antidiabetic treatment comparisons for 2 stage-least squares (2SLS) instrumental variable analysis on complete cases

			Year of follow-up					
**Outcome**	**Comparison**		**0.5**	**1**	**2**	**3**	**4**	**5**
**Difference in the change in HbA1c (mmol/mol) from baseline**		**N =**	46,900	42,441	32,364	23,082	15,126	8,022
	**DPP4i vs SU**	**Estimate**	1.84	-0.24	-1.81	-2.91	-1.46	-1.00
		**(95% CI)**	(0.43, 3.25)	(-1.76, 1.28)	(-3.61, -0.01)	(-4.96, -0.86)	(-4.12, 1.21)	(-4.62, 2.62)
	**SGLT2i vs SU**	**Estimate**	1.42	-4.86	-6.80	-11.58	-11.20	-13.80
		**(95% CI)**	(-0.36, 3.21)	(-7.19, -2.52)	(-10.00, -3.59)	(-15.97, -7.18)	(-17.16, -5.24)	(-24.31, -3.30)
	**SGLT2i vs DPP4i**	**Estimate**	-0.41	-4.61	-4.98	-8.67	-9.74	-12.81
		**(95% CI)**	(-2.52, 1.69)	(-7.30, -1.93)	(-8.74, -1.23)	(-13.67, -3.67)	(-16.51, -2.98)	(-24.637, -0.98)
**Difference in the change in BMI (kg/m^2) from baseline**		**N =**	33,508	34,431	25,809	18,253	11,577	5,903
	**DPP4i vs SU**	**Estimate**	-0.41	-0.71	-0.73	-0.58	-1.24	-0.83
		**(95% CI)**	(-0.68, -0.14)	(-0.95, -0.46)	(-1.05, -0.40)	(-1.02, -0.14)	(-1.80, -0.67)	(-1.72, 0.06)
	**SGLT2i vs SU**	**Estimate**	-1.41	-1.80	-1.78	-1.86	-2.11	-0.98
		**(95% CI)**	(-1.71, -1.12)	(-2.14, -1.47)	(-2.29, -1.28)	(-2.65, -1.07)	(-3.17, -1.06)	(-3.35, 1.39)
	**SGLT2i vs DPP4i**	**Estimate**	-1.00	-1.10	-1.06	-1.28	-0.88	-0.15
		**(95% CI)**	(-1.36, -0.64)	(-1.45, -0.74)	(-1.62, -0.49)	(-2.13, -0.43)	(-2.05, 0.29)	(-2.86, 2.56)
**Difference in the change in eGFR (mL/min/1.73m^2) from baseline**		**N =**	39,113	39,337	30,034	21,398	14,060	7,659
	**DPP4i vs SU**	**Estimate**	-0.06	0.28	1.28	3.73	3.87	4.70
		**(95% CI)**	(-0.76, 0.64)	(-0.50, 1.07)	(0.26, 2.30)	(2.57, 4.88)	(2.33, 5.41)	(2.43, 6.97)
	**SGLT2i vs SU**	**Estimate**	0.40	1.10	2.03	3.35	5.36	9.03
		**(95% CI)**	(-0.59, 1.39)	(0.05, 2.15)	(0.70, 3.36)	(1.21, 5.49)	(2.69, 8.03)	(5.00, 13.07)
	**SGLT2i vs DPP4i**	**Estimate**	0.46	0.82	0.75	-0.38	1.49	4.33
		**(95% CI)**	(-0.50, 1.42)	(-0.25, 1.88)	(-0.80, 2.30)	(-2.76, 2.00)	(-1.61, 4.59)	(-0.43, 9.09)

			Year of follow-up					
**Outcome**	**Comparison**		**0.5**	**1**	**2**	**3**	**4**	**5**
**Difference in the change in SBP (mm Hg) from baseline**		**N =**	40,588	41,049	30,967	21,972	13,832	7,100
	**DPP4i vs SU**	**Estimate**	-0.77	-0.02	-1.33	-0.79	-1.57	1.72
		**(95% CI)**	(-1.99, 0.44)	(-1.24, 1.20)	(-2.72, 0.06)	(-2.40, 0.82)	(-3.54, 0.40)	(-1.25, 4.69)
	**SGLT2i vs SU**	**Estimate**	-3.35	-1.57	-2.42	-4.12	-1.10	-3.14
		**(95% CI)**	(-4.89, -1.81)	(-3.11, -0.03)	(-4.35, -0.50)	(-6.64, -1.60)	(-4.36, 2.16)	(-8.44, 2.15)
	**SGLT2i vs DPP4i**	**Estimate**	-2.58	-1.56	-1.09	-3.33	0.47	-4.86
		**(95% CI)**	(-4.25, -0.91)	(-3.32, 0.21)	(-3.29, 1.10)	(-6.20, -0.46)	(-3.37, 4.30)	(-11.48, 1.76)

Supplementary table 15: Differences in the change in continuous clinical measures for the three second-line antidiabetic treatment comparisons for ordinary least squares (OLS) regression adjusted for measured confounders (complete cases)

			Year of follow-up					
**Outcome**	**Comparison**		**0.5**	**1**	**2**	**3**	**4**	**5**
**Difference in the change in HbA1c (mmol/mol) from baseline**		**N =**	46,900	42,441	32,364	23,082	15,126	8,022
	**DPP4i vs SU**	**Estimate (95% CI)**	3.30 (3.00, 3.60)	1.30 (0.96, 1.65)	0.09 (-0.32, 0.51)	-0.22 (-0.71, 0 .28)	-0.57 (-1.18, 0.03)	-0.67 (-1.52, 0.18)
	**SGLT2i vs SU**	**Estimate (95% CI)**	1.72 (1.36, 2.09)	-1.38 (-1.78, -0.98)	-1.73 (-2.26, -1.21)	-2.04 (-2.71, -1.38)	-1.66 (-2.59, -0.73)	-0.89 (-2.27, 0.49)
	**SGLT2i vs DPP4i**	**Estimate (95% CI)**	-1.58 (-1.94, -1.22)	-2.68 (-3.07, -2.29)	-1.83 (-2.34, -1.32)	-1.83 (-2.49, -1.16)	-1.09 (-1.99, -0.19)	-0.22 (-1.59, 1.15)
**Difference in the change in BMI (kg/m^2) from baseline**		**N =**	33,508	34,431	25,809	18,253	11,577	5,903
	**DPP4i vs SU**	**Estimate (95% CI)**	-0.53 (-0.59, -0.47)	-0.60 (-0.66, -0.54)	-0.52 (-0.59, -0.44)	-0.53 (-0.63, -0.44)	-0.50 (-0.62, -0.38)	-0.42 (-0.60, -0.23)
	**SGLT2i vs SU**	**Estimate (95% CI)**	-1.22 (-1.28, -1.15)	-1.27 (-1.35, -1.20)	-1.12 (-1.21, -1.03)	-0.98 (-1.11, -0.85)	-0.76 (-0.94, -0.58)	-0.83 (-1.11, -0.56)
	**SGLT2i vs DPP4i**	**Estimate (95% CI)**	-0.68 (-0.74, -0.63)	-0.67 (-0.73, -0.61)	-0.60 (-0.69, -0.52)	-0.45 (-0.57, -0.33)	-0.26 (-0.44, -0.09)	-0.42 (-0.67, -0.16)
**Difference in the change in eGFR (mL/min/1.73m^2) from baseline**		**N =**	39,113	39,337	30,034	21,398	14,060	7,659
	**DPP4i vs SU**	**Estimate (95% CI)**	-0.21 (-0.41, -0.01)	-0.19 (-0.40, 0.02)	0.08 (-0.17, 0.33)	-0.08 (-0.40, 0.24)	0.57 (0.18, 0.97)	0.46 (-0.15, 1.07)
	**SGLT2i vs SU**	**Estimate (95% CI)**	-0.10 (-0.35, 0.14)	0.02 (-0.25, 0.29)	0.79 (0.45, 1.13)	1.06 (0.61, 1.51)	1.65 (1.01, 2.28)	1.90 (1.04, 2.75)
	**SGLT2i vs DPP4i**	**Estimate (95% CI)**	0.10 (-0.11, 0.32)	0.21 (-0.03, 0.44)	0.71 (0.40, 1.02)	1.14 (0.70, 1.59)	1.07 (0.49, 1.66)	1.44 (0.54, 2.33)

			Year of follow-up					
**Outcome**	**Comparison**		**0.5**	**1**	**2**	**3**	**4**	**5**
**Difference in the change in SBP (mm Hg) from baseline**		**N =**	40,588	41,049	30,967	21,972	13,832	7,100
	**DPP4i vs SU**	**Estimate**	-0.71	-0.86	-0.54	-0.40	0.01	-0.06
		**(95% CI)**	(-1.02, -0.40)	(-1.16, -0.56)	(-0.90, -0.18)	(-0.84, 0.04)	(-0.51, 0.54)	(-0.79, 0.68)
	**SGLT2i vs SU**	**Estimate**	-2.35	-2.09	-1.83	-1.53	-0.86	-1.69
		**(95% CI)**	(-2.76, -1.94)	(-2.47, -1.71)	(-2.30, -1.37)	(-2.12, -0.93)	(-1.68, -0.04)	(-2.81, -0.57)
	**SGLT2i vs DPP4i**	**Estimate**	-1.64	-1.23	-1.30	-1.12	-0.87	-1.63
		**(95% CI)**	(-2.02, -1.26)	(-1.57, -0.88)	(-1.74, -0.85)	(-1.69, -0.56)	(-1.63, -0.11)	(-2.71, -0.55)

Supplementary table 16: Standard mean differences in variables before and after propensity score weighting for the primary outcome (change in HbA1c from baseline to 1-year)

**Standard mean differences - Before and after weighting - Change in HbA1c at 1 year**						
**Variable**	**Mean for SU**	**Mean for DPP4i**	**Std. diff**	**Wtd Mean for**	**Wtd Mean for**	**Std.Diff**
Balance for DPP4i vs SU before and after weighting						
Female	0.40	0.39	0.00	0.40	0.40	-0.01
HbA1c (mmol/mol)	80.28	71.01	-0.50	74.96	75.54	0.03
HbA1c squared (mmol/mol)	6917.40	5276.75	-0.50	5969.97	6117.76	0.05
Age, years	60.39	62.05	0.14	60.24	60.23	0.00
Age squared, years	3794.92	3998.51	0.14	3774.56	3772.92	0.00
BMI (kg/m^2)	31.52	32.26	0.11	32.89	32.56	-0.05
SBP (mm Hg)	131.69	131.86	0.01	131.97	131.84	-0.01
eGFR (mL/min/1.73m^2)	90.68	88.28	-0.13	90.58	90.76	0.01
DBP (mm Hg)	77.73	77.13	-0.07	77.77	77.76	0.00
HbA1c x Age	4813.01	4378.28	-0.32	4483.15	4514.23	0.02
HbA1c x BMI	2532.95	2297.67	-0.30	2466.61	2455.00	-0.01
Alcohol – non-drinker	0.11	0.10	-0.05	0.10	0.11	0.01
Alcohol – ex-drinker	0.30	0.30	0.00	0.29	0.30	0.01
Amputation	0.01	0.01	-0.01	0.01	0.01	0.00
Ethnicity – Black/Mixed/Other	0.08	0.06	-0.10	0.06	0.07	0.01
Ethnicity – South Asian	0.14	0.13	-0.02	0.13	0.13	0.01
Blindness	0.02	0.01	-0.02	0.01	0.01	0.00
History of cancer	0.17	0.16	-0.01	0.15	0.15	0.00
Heart failure	0.05	0.06	0.02	0.05	0.05	0.00
Hospitalisation in the past year	0.26	0.23	-0.06	0.24	0.24	-0.01
Hypoglycaemia	0.01	0.01	0.00	0.01	0.01	0.01
Previous myocardial infarction	0.06	0.06	0.02	0.06	0.06	0.00
RASi prescription	0.50	0.56	0.11	0.53	0.53	0.00

Standard mean differences - Before and after weighting - Change in HbA1c at 1 year						
**Variable**	**Mean for SU**	**Mean for DPP4i**	**Std. diff**	**Wtd Mean for**	**Wtd Mean for**	**Std.Diff**
Smoking – non-smoker	0.22	0.21	-0.02	0.21	0.22	0.01
Smoking – ex-smoker	0.50	0.54	0.07	0.53	0.52	-0.01
Statin prescription	0.71	0.75	0.10	0.73	0.73	0.00
Previous stroke	0.05	0.05	-0.02	0.05	0.05	0.00
Ischaemic heart disease	0.17	0.19	0.05	0.18	0.18	0.00
Unstable angina	0.03	0.03	0.03	0.03	0.03	0.00
Proteinuria	0.15	0.15	-0.03	0.14	0.14	0.00
General practice size	11430.50	11605.67	0.02	11526.03	11560.54	0.00
Days since T2DM diagnosis	2170.79	2436.91	0.13	2264.09	2250.86	-0.01
IMD – 1 (least deprived)	0.14	0.15	0.03	0.15	0.15	-0.01
IMD – 2	0.17	0.18	0.03	0.18	0.18	0.00
IMD – 3	0.20	0.19	-0.01	0.19	0.19	-0.01
IMD – 4	0.24	0.22	-0.04	0.23	0.23	0.00
Region – North East	0.07	0.02	-0.27	0.04	0.04	0.01
Region – North West	0.18	0.22	0.10	0.21	0.21	-0.01
Region – Yorkshire	0.04	0.04	0.01	0.04	0.04	0.00
Region – East Midlands	0.03	0.03	-0.03	0.03	0.03	0.00
Region – West Midlands	0.14	0.19	0.14	0.17	0.17	0.01
Region – East England	0.05	0.04	-0.04	0.04	0.04	0.00
Region – South East	0.14	0.15	0.03	0.17	0.16	-0.01
Region – South West	0.11	0.12	0.04	0.11	0.12	0.01
Year – 2016	0.22	0.19	-0.06	0.19	0.19	0.00
Year – 2017	0.17	0.20	0.07	0.18	0.19	0.01
Year – 2018	0.15	0.22	0.18	0.20	0.19	-0.01
Year – 2019	0.10	0.15	0.13	0.15	0.14	-0.01
Year – 2020	0.06	0.08	0.07	0.09	0.09	0.01

Standard mean differences - Before and after weighting - Change in HbA1c at 1 year						
**Variable**	**Mean for SU**	**Mean for DPP4i**	**Std. diff**	**Wtd Mean for**	**Wtd Mean for**	**Std.Diff**
HbA1c x Female	31.18	27.72	-0.09	29.52	29.41	0.00
Balance for SGLT2i vs SU before and after weighting						
Female	0.40	0.39	0.00	0.40	0.40	-0.01
HbA1c (mmol/mol)	80.28	71.01	-0.50	74.96	75.54	0.03
HbA1c squared (mmol/mol)	6917.40	5276.75	-0.50	5969.97	6117.76	0.05
Age, years	60.39	62.05	0.14	60.24	60.23	0.00
Age squared, years	3794.92	3998.51	0.14	3774.56	3772.92	0.00
BMI (kg/m^2)	31.52	32.26	0.11	32.89	32.56	-0.05
SBP (mm Hg)	131.69	131.86	0.01	131.97	131.84	-0.01
eGFR (mL/min/1.73m^2)	90.68	88.28	-0.13	90.58	90.76	0.01
DBP (mm Hg)	77.73	77.13	-0.07	77.77	77.76	0.00
HbA1c x Age	4813.01	4378.28	-0.32	4483.15	4514.23	0.02
HbA1c x BMI	2532.95	2297.67	-0.30	2466.61	2455.00	-0.01
Alcohol – non-drinker	0.11	0.10	-0.05	0.10	0.11	0.01
Alcohol – ex-drinker	0.30	0.30	0.00	0.29	0.30	0.01
Amputation	0.01	0.01	-0.01	0.01	0.01	0.00
Ethnicity – Black/Mixed/Other	0.08	0.06	-0.10	0.06	0.07	0.01
Ethnicity – South Asian	0.14	0.13	-0.02	0.13	0.13	0.01
Blindness	0.02	0.01	-0.02	0.01	0.01	0.00
History of cancer	0.17	0.16	-0.01	0.15	0.15	0.00
Heart failure	0.05	0.06	0.02	0.05	0.05	0.00
Hospitalisation in the past year	0.26	0.23	-0.06	0.24	0.24	-0.01
Hypoglycaemia	0.01	0.01	0.00	0.01	0.01	0.01
Previous myocardial infarction	0.06	0.06	0.02	0.06	0.06	0.00
RASi prescription	0.50	0.56	0.11	0.53	0.53	0.00
Smoking – non-smoker	0.22	0.21	-0.02	0.21	0.22	0.01

Standard mean differences - Before and after weighting - Change in HbA1c at 1 year						
**Variable**	**Mean for SU**	**Mean for DPP4i**	**Std. diff**	**Wtd Mean for**	**Wtd Mean for**	**Std.Diff**
Smoking – ex-smoker	0.50	0.54	0.07	0.53	0.52	-0.01
Statin prescription	0.71	0.75	0.10	0.73	0.73	0.00
Previous stroke	0.05	0.05	-0.02	0.05	0.05	0.00
Ischaemic heart disease	0.17	0.19	0.05	0.18	0.18	0.00
Unstable angina	0.03	0.03	0.03	0.03	0.03	0.00
Proteinuria	0.15	0.15	-0.03	0.14	0.14	0.00
General practice size	11430.50	11605.67	0.02	11526.03	11560.54	0.00
Days since T2DM diagnosis	2170.79	2436.91	0.13	2264.09	2250.86	-0.01
IMD – 1 (least deprived)	0.14	0.15	0.03	0.15	0.15	-0.01
IMD – 2	0.17	0.18	0.03	0.18	0.18	0.00
IMD – 3	0.20	0.19	-0.01	0.19	0.19	-0.01
IMD – 4	0.24	0.22	-0.04	0.23	0.23	0.00
Region – North East	0.07	0.02	-0.27	0.04	0.04	0.01
Region – North West	0.18	0.22	0.10	0.21	0.21	-0.01
Region – Yorkshire	0.04	0.04	0.01	0.04	0.04	0.00
Region – East Midlands	0.03	0.03	-0.03	0.03	0.03	0.00
Region – West Midlands	0.14	0.19	0.14	0.17	0.17	0.01
Region – East England	0.05	0.04	-0.04	0.04	0.04	0.00
Region – South East	0.14	0.15	0.03	0.17	0.16	-0.01
Region – South West	0.11	0.12	0.04	0.11	0.12	0.01
Year – 2016	0.22	0.19	-0.06	0.19	0.19	0.00
Year – 2017	0.17	0.20	0.07	0.18	0.19	0.01
Year – 2018	0.15	0.22	0.18	0.20	0.19	-0.01
Year – 2019	0.10	0.15	0.13	0.15	0.14	-0.01
Year – 2020	0.06	0.08	0.07	0.09	0.09	0.01
HbA1c x Female	31.18	27.72	-0.09	29.52	29.41	0.00

Supplementary table 17: Mean, minimum, and maximum unstabilised weights by treatment arm for each continuous outcome in the inverse probability of treatment weighting (IPTW) analysis

**Outcome / Follow up year**	**SU**			**DPP4i**			**SGLT2i**			**Total**		
	**Mean**	**Minimum**	**Maximum**	**Mean**	**Minimum**	**Maximum**	**Mean**	**Minimum**	**Maximum**	**Mean**	**Minimum**	**Maximum**
**HbA1c**												
0.5	3.04	1.02	31.75	2.17	1.16	45.58	4.80	1.11	223.29	2.98	1.02	223.29
1	2.99	1.01	35.24	2.13	1.12	69.00	5.08	1.10	113.78	2.98	1.01	112.78
2	2.84	1.02	40.43	2.11	1.16	74.75	5.82	1.16	202.23	2.98	1.01	202.23
3	2.61	1.02	29.70	2.12	1.17	21.02	6.93	1.21	219.47	2.97	1.02	219.47
4	2.35	1.01	18.33	2.22	1.23	101.52	7.89	1.24	101.52	2.96	1.01	101.52
5	2.19	1.02	31.44	2.33	1.26	24.35	9.10	1.28	245.77	3.00	1.02	245.77
**BMI**												
0.5	3.17	1.01	39.38	2.16	1.10	33.11	4.45	1.09	78.06	2.97	1.01	78.06
1	3.10	1.01	34.77	2.11	1.14	46.35	4.89	1.10	86.87	2.97	1.01	86.87
2	2.91	1.01	33.57	2.09	1.15	32.02	5.63	1.13	232.93	2.98	1.01	232.93
3	2.68	1.03	27.67	2.10	1.16	24.82	6.62	1.21	190.69	2.98	1.03	190.69
4	2.45	1.02	25.13	2.17	1.23	20.59	7.62	1.20	288.58	2.98	1.02	288.58
5	2.22	1.02	12.91	2.31	1.23	24.33	9.01	1.28	225.04	2.02	1.02	225.04
**eGFR**												
0.5	3.01	1.01	32.59	2.14	1.16	48.63	4.81	1.10	220.61	2.99	1.01	220.61
1	3.04	1.01	38.19	2.11	1.12	58.50	5.05	1.10	100.22	2.97	1.01	100.22
2	2.88	1.01	35.32	2.09	1.17	81.15	5.84	1.15	210.11	2.98	1.01	210.11
3	2.66	1.01	30.92	2.09	1.16	35.24	6.91	1.20	224.47	2.98	1.01	224.47
4	2.40	1.01	18.89	2.19	1.23	23.32	7.89	1.19	105.86	2.96	1.01	105.86
5	2.23	1.02	28.32	2.29	1.22	23.98	9.01	1.23	298.99	3.00	1.02	298.99
**SBP**												
0.5	2.99	1.91	32.16	2.14	1.11	43.93	5.06	1.10	234.98	2.98	1.01	234.98
1	2.97	1.01	35.40	2.12	1.16	55.75	2.12	1.16	55.75	5.22	1.10	211.14
2	2.78	1.02	33.66	2.11	1.16	56.65	5.93	1.12	221.53	2.98	1.02	221.53
3	2.59	1.02	27.41	2.12	1.18	50.55	7.13	1.21	209.47	2.98	1.02	209.47

Outcome / Follow up year	SU			DPP4i			SGLT2i			Total		
	Mean	Minimum	Maximum	Mean	Minimum	Maximum	Mean	Minimum	Maximum	Mean	Minimum	Maximum
4	2.33	1.01	16.96	2.23	1.23	23.13	8.03	1.20	110.06	2.96	1.01	110.06
5	2.17	1.02	17.64	2.34	1.27	20.96	9.38	1.28	232.45	3.01	1.02	232.45

BMI: body-mass index; DPP4i: dipeptidyl peptidase 4 inhibitors; eGFR: estimated glomerular filtration rate; HbA1c: haemoglobin A1c; SBP: systolic blood pressure; SGLT2i: sodium-glucose co-transporter 2 inhibitors; SU: sulfonylureas

Supplementary table 18: Differences in the change in continuous clinical measures for the three second-line antidiabetic treatment comparisons (inverse probability of treatment weighting (IPTW), complete cases)

			Year of follow-up					
**Outcome**	**Comparison**		**0.5**	**1**	**2**	**3**	**4**	**5**
**Difference in the change in HbA1c (mmol/mol) from baseline**		**N =**	46,900	42,441	32,364	23,082	15,126	8,022
	**DPP4i vs SU**	**Estimate (95% CI)**	2.72 (2.24, 3.19)	0.83 (0.33, 1.32)	-0.51 (-1.15, 0.13)	-0.65 (-1.21, -0.09)	-1.07 (-1.74, -0.39)	-0.97 (-1.90, -0.05)
	**SGLT2i vs SU**	**Estimate (95% CI)**	1.18 (0.51, 1.85)	-1.88 (-2.61, -1.15)	-1.61 (-2.44, -0.79)	-2.04 (-3.00, -1.08)	-0.96 (-2.05, 0.12)	-0.62 (-2.27, 1.04)
	**SGLT2i vs DPP4i**	**Estimate (95% CI)**	-1.54 (-2.29, -0.78)	-2.70 (-3.49, -1.91)	-1.10 (-2.03, -0.18)	-1.40 (-2.38, -0.42)	0.10 (-1.02, 1.22)	0.36 (-1.36, 2.07)
**Difference in the change in BMI (kg/m²) from baseline**		**N =**	33,508	34,431	25,809	18,253	11,577	5,903
	**DPP4i vs SU**	**Estimate (95% CI)**	-0.51 (-0.58, -0.44)	-0.57 (-0.65, -0.49)	-0.42 (-0.51, -0.34)	-0.47 (-0.60, -0.35)	-0.44 (-0.58, -0.31)	-0.29 (-0.54, -0.03)
	**SGLT2i vs SU**	**Estimate (95% CI)**	-0.44 (-1.32, -1.16)	-1.30 (-1.39, -1.20)	-1.08 (-1.20, -0.96)	-0.97 (-1.13, -0.80)	-0.81 (-1.05, -0.57)	-0.56 (-1.00, -0.12)
	**SGLT2i vs DPP4i**	**Estimate (95% CI)**	-0.73 (-0.79, -0.67)	-0.73 (-0.81, -0.65)	-0.66 (-0.76, -0.55)	-0.49 (-0.64, -0.35)	-0.37 (-0.60, -0.13)	-0.27 (-0.69, 0.15)
**Difference in the change in eGFR (mL/min/1.73m²) from baseline**		**N =**	39,113	39,337	30,034	21,398	14,060	7,659
	**DPP4i vs SU**	**Estimate (95% CI)**	-0.19 (-0.41, 0.04)	-0.19 (-0.42, 0.04)	0.02 (-0.26, 0.30)	-0.10 (-.47, 0.26)	0.53 (0.10, 0.96)	0.44 (-0.19, 1.06)
	**SGLT2i vs SU**	**Estimate (95% CI)**	-0.08 (-0.43, 0.27)	-0.08 (-0.42, 0.25)	0.96 (0.49, 1.44)	1.24 (0.50, 1.98)	1.60 (0.75, 2.44)	3.24 (1.10, 5.37)
	**SGLT2i vs DPP4i**	**Estimate (95% CI)**	0.10 (-0.23, 0.44)	0.11 (-0.22, 0.43)	0.94 (0.48, 1.41)	1.34 (0.60, 2.08)	1.06 (0.23, 1.90)	2.80 (0.66, 4.94)
		**N =**	40,588	41,049	30,967	21,972	13,832	7,100

			Year of follow-up					
**Outcome**	**Comparison**		**0.5**	**1**	**2**	**3**	**4**	**5**
**Difference in the change in SBP (mm Hg) from baseline**	**DPP4i vs SU**	**Estimate**	-0.60	-0.85	-0.28	-0.50	0.01	0.09
		**(95% CI)**	(-0.95, -0.25)	(-1.20, -0.50)	(-0.68, 0.12)	(-0.96, -0.04)	(-0.55, .56)	(-0.72, 0.90)
	**SGLT2i vs SU**	**Estimate**	-2.25	-2.10	-1.61	-1.33	-1.30	-1.02
		**(95% CI)**	(-2.90, -1.59)	(-2.81, -1.40)	(-2.57, -0.65	(-2.33, -0.33)	(-2.33, -o.27)	(-4.06, 2.02)
	**SGLT2i vs DPP4i**	**Estimate**	-1.64	-1.24	-1.33	-0.83	-1.31	-1.16
		**(95% CI)**	(-2.28, -1.00)	(-1.93, -0.55)	(-2.27, -0.39)	(-1.82, 0.16)	(-2.35, -0.28)	(-4.15, 1.84)

Supplementary table 19: Differences in the change in continuous clinical measures for the three second-line antidiabetic treatment comparisons (inverse probability of treatment weighting - regression adjustment (IPTW-RA or 'weighted regression') analysis, complete cases)

			Year of follow-up					
**Outcome**	**Comparison**		**0.5**	**1**	**2**	**3**	**4**	**5**
**Difference in the change in HbA1c (mmol/mol) from baseline**		**N =**	46,900	42,441	32,364	23,082	15,126	8,022
	**DPP4i vs SU**	**Estimate (95% CI)**	3.29 (2.97, 3.62)	1.31 (0.94, 1.68)	0.09 (-0.35, 0.52)	-0.29 (-0.79, 0.20)	-0.75 (-1.36, -0.14)	-0.56 (-1.42, 0.29)
	**SGLT2i vs SU**	**Estimate (95% CI)**	1.73 (1.30, 2.17)	-1.23 (-1.73, -0.73	-1.30 (-2.00, -0.60)	-1.84 (-2.65, -1.03)	-1.13 (-2.23, -0.03)	-1.00 (-2.41, 0.41)
	**SGLT2i vs DPP4i**	**Estimate (95% CI)**	-1.56 (-1.99, -1.13)	-2.54 (-3.02, -2.05)	-1.38 (-2.06, -0.71)	-1.55 (-2.34, -0.76)	-0.37 (-1.47, 0.72)	-0.44 (-1.87, 0.99)
**Difference in the change in BMI (kg/m^2) from baseline**		**N =**	33,508	34,431	25,809	18,253	11,577	5,903
	**DPP4i vs SU**	**Estimate (95% CI)**	-0.53 (-0.60, -0.46)	-0.59 (-0.67, -0.52)	-0.46 (-0.54, -0.38)	-0.52 (-0.62, -0.41)	-0.49 (-0.62, -0.37)	-0.35 (-0.55, -0.14)
	**SGLT2i vs SU**	**Estimate (95% CI)**	-1.23 (-1.31, -1.15)	-1.30 (-1.39, -1.22)	-1.08 (-1.18, -0.97)	-0.95 (-1.09, -0.81)	-0.81 (-1.00, -0.63)	-0.70 (-0.97, -0.43)
	**SGLT2i vs DPP4i**	**Estimate (95% CI)**	-0.70 (-0.76, -0.64)	-0.71 (-0.78, -0.64)	-0.62 (-0.71, -0.52)	-0.43 (-0.56, -0.31)	-0.32 (-0.50, -0.14)	-0.35 (-0.60, -0.10)
**Difference in the change in eGFR (mL/min/1.73m^2) from baseline**		**N =**	39,113	39,337	30,034	21,398	14,060	7,659
	**DPP4i vs SU**	**Estimate (95% CI)**	-0.17 (-0.39, 0.04)	-0.16 (-0.38, 0.05)	0.07 (-0.19, 0.34)	-0.09 (-0.43, 0.25)	0.54 (0.12, 0.96)	0.42 (-0.18, 1.03)
	**SGLT2i vs SU**	**Estimate (95% CI)**	0.10 (-0.24, 0.43)	0.12 (-0.23, 0.47)	1.21 (0.75, 1.67)	1.42 (0.80, 2.04)	1.59 (0.70, 2.49)	2.79 (1.72, 3.85)
	**SGLT2i vs DPP4i**	**Estimate (95% CI)**	0.27 (-0.04, 0.58)	0.28 (-0.05, 0.61)	1.14 (0.70, 1.57)	1.51 (0.90, 2.12)	1.06 (0.16, 1.95)	2.36 (1.29, 3.44)
		**N =**	40,588	41,049	30,967	21,972	13,832	7,100

			Year of follow-up					
**Outcome**	**Comparison**		**0.5**	**1**	**2**	**3**	**4**	**5**
**Difference in the change in SBP (mm Hg) from baseline**	**DPP4i vs SU**	**Estimate**	-0.66	-0.93	-0.37	-0.52	-0.06	-0.08
		**(95% CI)**	(-0.99, -0.33)	(-1.26, -0.59)	(-0.74, 0.00)	(-0.95, -.085)	(-0.59, 0.47)	(-0.83, 0.67)
	**SGLT2i vs SU**	**Estimate**	-2.47	-2.29	-1.84	-1.34	-0.90	-1.67
		**(95% CI)**	(-2.97, -1.96)	(-2.83, -1.75)	(-2.45, -1.23)	(-2.06, -0.63)	(-1.80, 0.09)	(-3.15, -0.19)
	**SGLT2i vs DPP4i**	**Estimate**	-1.81	-1.36	-1.48	-0.83	-0.84	-1.58
		**(95% CI)**	(-2.29, -1.34)	(-1.86, -0.86)	(-2.06, -0.90)	(-1.53, -0.14)	(-1.82, 0.15	(-3.06, -0.10)

Supplementary table 20: Differences in the change in HbA1c for the three second-line antidiabetic treatment comparisons (inverse probability of treatment weighting – regression adjustment (IPTW-RA or 'weighted regression'), complete cases – asymmetric trimming[28, 29]

			0.5 Year of follow-up		1 Year of follow-up	
**Outcome**	**Comparison**		**Original**	**Asymmetrically Trimmed**	**Original**	**Asymmetrically Trimmed**
**Difference in the change in HbA1c (mmol/mol) from baseline**		**N =**	46,900	46,177	42,441	41,779
	**DPP4i vs SU**	**Estimate (95% CI)**	2.72 (2.24, 3.19)	3.24 (2.87, 3.61)	0.83 (0.33, 1.32)	1.35 (0.95, 1.75)
	**SGLT2i vs SU**	**Estimate (95% CI)**	1.18 (0.51, 1.85)	1.65 (1.20, 2.10)	-1.88 (-2.61, -1.15)	-1.21 (-1.72, -0.70)
	**SGLT2i vs DPP4i**	**Estimate (95% CI)**	-1.54 (-2.29, -0.78)	-1.58 (-2.06, -1.11)	-2.70 (-3.49, -1.91)	-2.57 (-3.08, -2.06)

Supplementary table 21: Main features of the study populations and comparison groups for the PERMIT study and relevant randomised controlled trials (RCTs) that included a randomisation to either SGLT2i or DPP4i

**Study**	**Year**	**Key study eligibility criteria**	**'Active' treatment**	**'Comparator'**	**Antidiabetic treatment prior to randomisation, % of study population[1]**				
					**Metformin**	**SU**	**DPP4i**	**Insulin**	**GLP1-RA**
PERMIT[30]	2023	T2DM, general 2nd line initiators, eGFR>30mL/min/1.73m^2, no antidiabetic treatment prior to randomisation except metformin	SGLT2i or DPP4i	DPP4i or SU	100	0	0	0	0
EMPA-REG[31]	2015	T2DM, established CVD, HbA1c 7-9%, eGFR>30mL/min/1.73m^2, BMI<45kg/m^2	SGLT2i (empagliflozin)	Placebo	74	42	11	48	3
CANVAS-R[32]	2017	T2DM, at high-CVD risk, HbA1c 7.0-10.5%	SGLT2i (canagliflozin)	Placebo	77	43	12	50	4
DECLARE-TIMI 58[33]	2019	T2DM, at high-CVD risk, HbA1c 6.5-12, creatinine clearance ≥60mL/min	SGLT2i (dapagliflozin)	Placebo	82	43	17	41	4
CAROLINA[34]	2019	T2DM, at high-CVD risk, HbA1c 6.5-8.5%	DPP4i (linagliptin)	SU (glimepiride)	84	29	Unknown	Unknown	Unknown
ERTUGLIFLOZIN CVOT[35]	2020	T2DM, established CVD, HbA1c 7.0-10.5%, eGFR>30mL/min/1.73m^2	SGLT2i (ertugliflozin)	Placebo	77	41	11	48	3
CREDENCE[36]	2019	T2DM, CKD, HbA1c 6.5-12.0%	SGLT2i (canagliflozin)	Placebo	58	29	17	66	4
EMPA-Kidney[37]	2023	People with (96.6%) or without (3.4%) T2DM, eGFR 45-90mL/min/1.73m^2	SGLT2i (empagliflozin)	Placebo	10	9	13	25	5
GRADE[38, 39]	2023	T2DM, excluded if major CVD in past year, HbA1c 6.8-8.5%, treated with MET alone	DPP4i (sitagliptin)	SU (glimepiride)	100	0	0	0	0
DAPA-HF[40]	2019	People with (41.8%) or without (58.2%) T2DM, with heart failure, eGFR>30mL/min/1.73m^2	SGLT2i (dapagliflozin)	Placebo	51	23	16	27	1

Study	Year	Key study eligibility criteria	'Active' treatment	'Comparator'	Antidiabetic treatment prior to randomisation, % of study population[1]				
					**Metformin**	**SU**	**DPP4i**	**Insulin**	**GLP1-RA**
DAPA-CKD[41]	2020	People with (67%) or without (33%) T2DM, eGFR 25-75mL/min/1.73m^2, ACEI/ARB	SGLT2i (dapagliflozin)	Placebo	Unknown	Unknown	Unknown	Unknown	Unknown
EMPEROR-Reduced[42]	2020	People with (49.8%) or without (50.2%) T2DM, heart failure, BMI<45kg/m^2	SGLT2i (empagliflozin)	Placebo	Unknown	Unknown	Unknown	Unknown	Unknown

ACEI/ARB: angiotensin converting enzyme inhibitor/angiotensin receptor blocker: BMI: body-mass index; CKD: chronic kidney disease; CVD: cardiovascular disease; DPP4i: dipeptidyl peptidase-4 inhibitor; eGFR: estimated glomerular filtration rate; GLP1-RA: glucagon-like peptide-1 receptor agonist; HbA1c: haemoglobin A1c; MACE-3: 3-point major adverse cardiovascular event; MAKE: major adverse kidney event; SGLT2i: sodium-glucose co-transporter 2 inhibitor; SU: sulfonylurea; T2DM: type 2 diabetes mellitus

[1]Note that for some studies people received more than one antidiabetic treatment prior to randomisation. For these studies the sum of the percentages can exceed 100%.

Supplementary table 22: Results from main analysis of the PERMIT study and those from relevant RCTs for common endpoints

			**Outcome, All reported with hazard ratios (HR) (95% confidence intervals (CI))**				
**Study**	**Year**	**Simplified summary of study population**	**Heart failure hospitalisation**	**MACE-3**	**MAKE**	**40% decline in eGFR**	**All-cause mortality**
**Studies comparing DPP4i versus SU**							
PERMIT (DPP4i v SU)	2023	T2DM	1.41 (0.73, 2.71)	1.09 (0.70, 1.69)	0.72 (0.50, 1.03)	0.66 (0.37, 1.17)	0.82 (0.51, 1.32)
CAROLINA[34] (DPP4i v SU)	2019	T2DM, CVD	1.21 (0.92, 1.59)	0.98 (0.84, 1.14)	N/A	N/A	0.91 (0.78, 1.06)
GRADE[39] (DPP4i v SU)	2023	T2DM	0.99 (0.60, 1.64)	1.16 (0.82, 1.64)	0.93 (0.75, 1.18)	N/A	0.93 (0.61, 1.45)
**Studies comparing SGLT2i versus placebo or active comparator**							
PERMIT (SGLT2i v SU)	2023	T2DM	0.46 (0.20, 1.05)	0.99 (0.61, 1.62)	0.79 (0.51, 1.23)	0.42 (0.22, 0.81)	1.14 (0.64, 2.03)
PERMIT (SGLT2i v DPP4i)	2023	T2DM	0.32 (0.12, 0.85)	0.91 (0.51, 1.63)	1.11 (0.66, 1.84)	0.64 (0.29, 0.81)	1.39 (0.71, 2.74)
EMPA-REG[31] (SGLT2i v placebo)	2015	T2DM, CVD	0.65 (0.50, 0.85)	0.86 (0.74, 0.99)	N/A	N/A	0.68 (0.57, 0.82)
CANVAS-R[32] (SGLT2i v placebo)	2017	T2DM, CVD	0.67 (0.52, 0.87)	0.86 (0.75, 0.97)	0.60 (0.47, 0.77)	N/A	0.87 (0.74, 1.01)
DECLARE-TIMI 58[33] (SGLT2i v placebo)	2019	T2DM, CVD	0.76 (0.61, 0.88)	0.93 (0.84, 1.03)	0.53 (0.43, 0.66)	N/A	0.93 (0.82, 1.04)
ERTUGLIFLOZIN CVOT[35] (SGLT2i v placebo)	2020	T2DM, CVD	0.70 (0.54, 0.90)	0.97 (0.85, 1.11)	0.81 (0.63, 1.04)	N/A	0.93 (0.80, 1.08)
CREDENCE[36] (SGLT2i v placebo)	2019	T2DM, CKD	0.61 (0.47, 0.80)	0.80 (0.67, 0.95)	0.70 (0.59, 0.82)	0.60 (0.48, 0.76)	0.83 (0.68, 1.02)
EMPA-Kidney[37] (SGLT2i v placebo)	2023	T2DM, CKD	0.84 (0.67, 1.07)	N/A	0.72 (0.64, 0.82)	0.70 (0.61, 0.81)	0.87 (0.67, 1.07)
DAPA-HF[40] (SGLT2i v placebo)	2019	CVD	0.70 (0.59, 0.83)	N/A	0.71 (0.44, 1.16)	N/A	0.83 (0.71, 0.97)
DAPA-CKD[41]	2020	CKD	0.71 (0.55, 0.92)	N/A	0.61 (0.51, 0.72)	0.53 (0.42, 0.67)	0.69 (0.53, 0.88)

			Outcome,<br>All reported with hazard ratios (HR) (95% confidence intervals (CI))				
**Study**	**Year**	**Simplified summary of study population**	**Heart failure hospitalisation**	**MACE-3**	**MAKE**	**40% decline in eGFR**	**All-cause mortality**
(SGLT2i v placebo)							
EMPEROR-Reduced[42] (SGLT2i v placebo)	2020	CVD	0.69 (0.59, 0.81)	N/A	0.50 (0.32, 0.77)	N/A	0.92 (0.77, 1.10)

CKD: chronic kidney disease; CVD: cardiovascular disease; DPP4i: dipeptidyl peptidase-4 inhibitor; eGFR: estimated glomerular filtration rate; MACE-3: 3-point major adverse cardiovascular event; MAKE: major adverse kidney event; N/A: not applicable (in this case, the study did not include this outcome); SGLT2i: sodium-glucose co-transporter 2 inhibitor; SU: sulfonylurea; T2DM: type 2 diabetes mellitus

SUPPLEMENTARY FIGURES

Supplementary figure 1A: Directed acyclic graph (DAG) illustrating the causal relationship between the instrument, exposure, and primary outcome (change in HbA1c from baseline to 1-year follow-up)

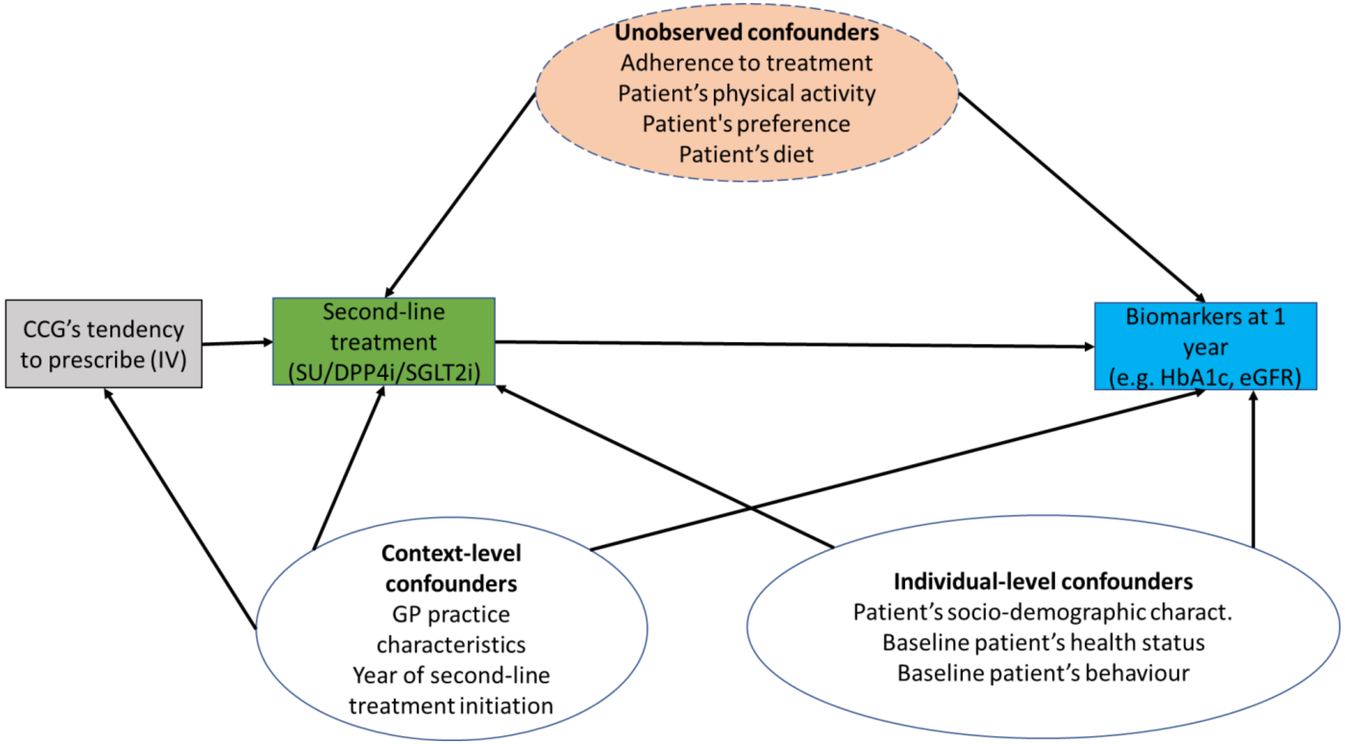

Commentary: This directed acyclic graph illustrates that the receipt of second-line treatment is subject to unmeasured and (context- and individual-level) observed factors that confounds the link between treatment and the outcome of interest (biomarkers at 1 year). This figure suggests that the CCG's tendency to prescribe (the proposed instrumental variable) predicts the second-line treatment received by a patient registered in that CCG, but does not have a direct effect on the health outcome of interest. That is, it is assumed that the only path through which the CCGs tendency to prescribe influences the biomarkers at 1 year is through its influence in the treatment received. Thus, this reflects the explicit assumption of an IV design that the instrument is not independently associated with outcomes, unobserved confounders and individual-level confounders. The DAG allows for an association between context-level confounders (such as GP practice list size) and the IV as

larger practices may have different prescription patterns compared to smaller practices. The individual-level confounders considered in this hypothesised causal diagram were classified in three broad categories: patient's socio-demographic characteristics (age, sex, etc.), baseline health status (e.g. relevant comorbidities, biomarkers and medications such as statins and renins), and baseline behaviour (alcohol and smoking status). For simplicity, this figure does not reflect all the existing correlations amongst different factors, for example the one existing between unobserved and individual-level confounders.

Supplementary figure 1B: Directed acyclic graph (DAG) illustrating the causal relationship between the instrument, exposure, and all-cause mortality (secondary outcome)

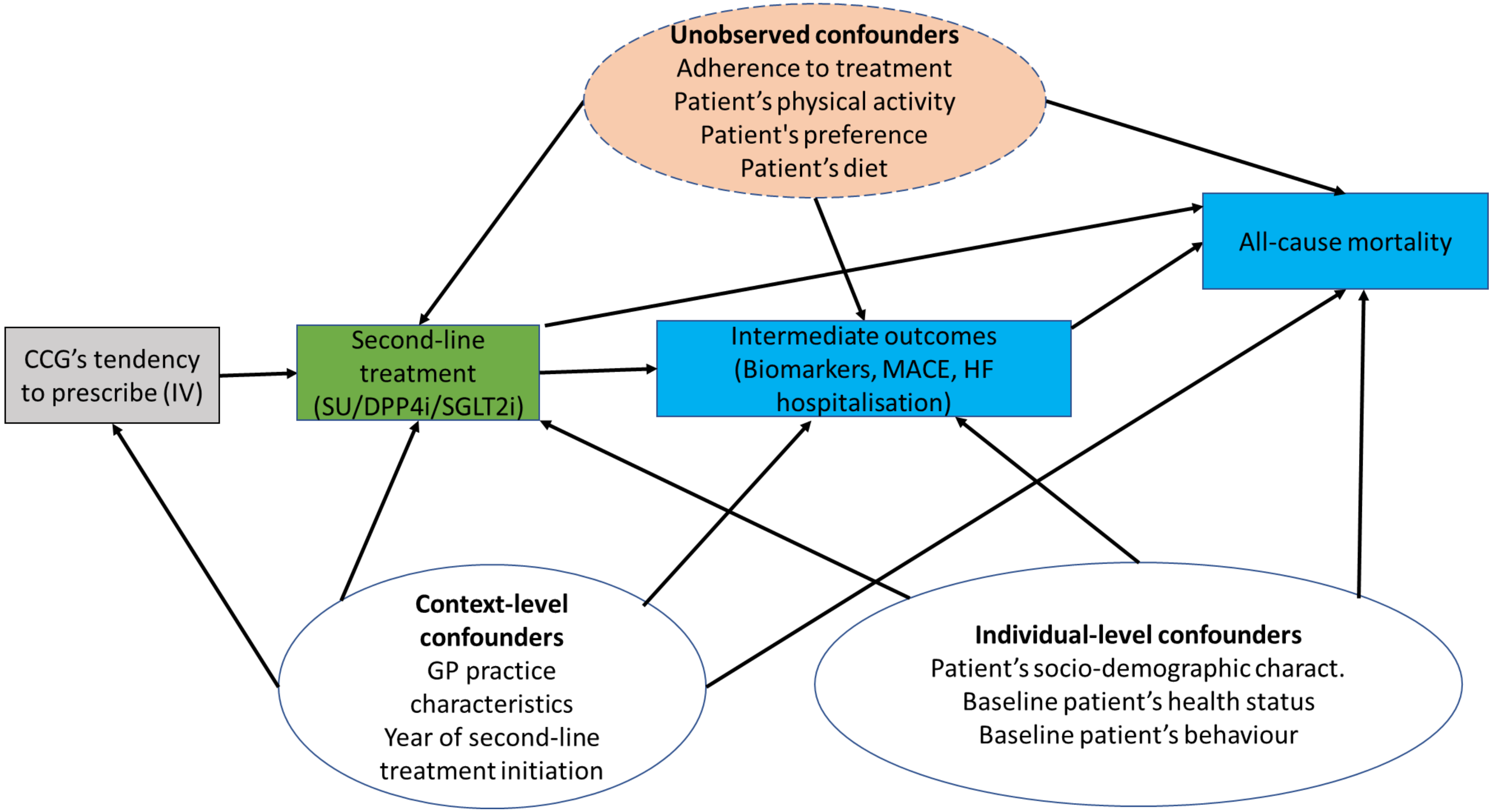

Commentary:

This DAG builds on the same structure as the previous one, but considers biomarkers at one year from intensification along with other adverse events such as MACE and hospitalisations due to heart failure as intermediate outcomes on the pathway from treatment to all-cause

mortality. It can be seen that all-cause mortality, the long-term outcome of interest, is also subject to unobserved and measured confounders, but that the only path through which the IV influences both intermediate and long-term outcomes is through its influence in the treatment received.

Supplementary figure 2A: Covariate balance plots according to levels of the instrumental variable for SU

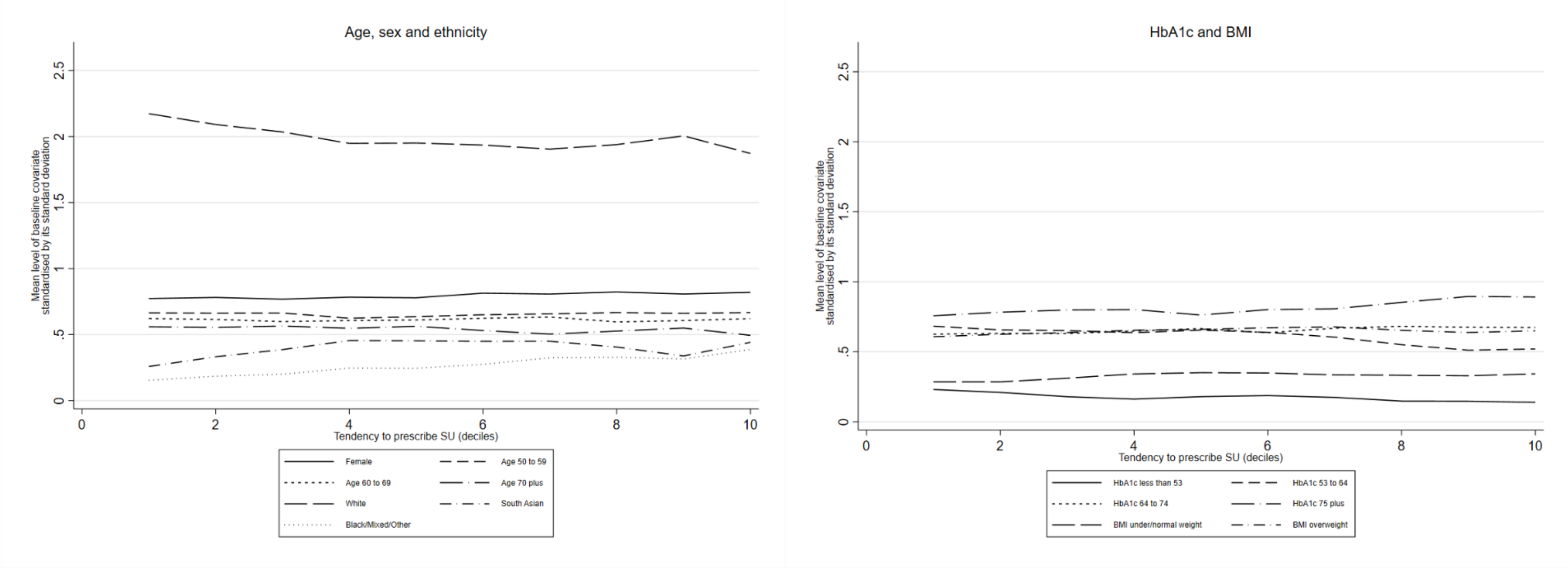

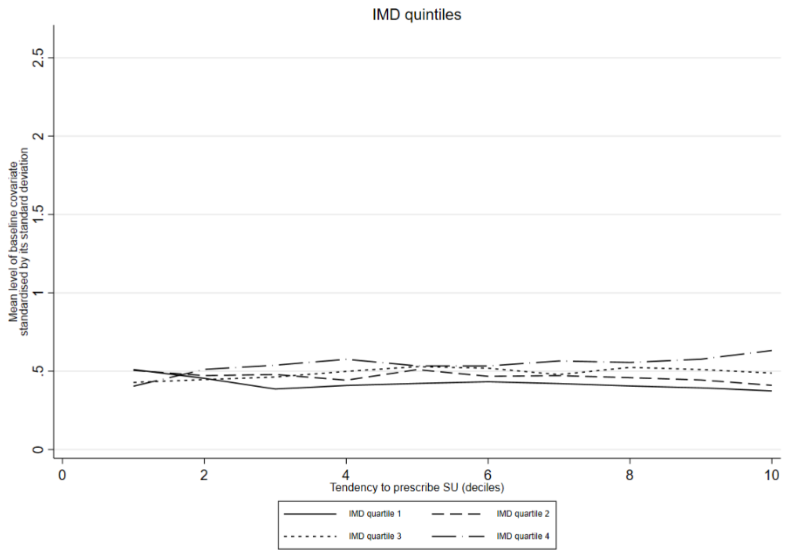
IMD quintiles
Mean level of baseline covariate standardised by its standard deviation
0
.5
1
1.5
2
2.5
0
2
4
6
8
10
Tendency to prescribe SU (deciles)
IMD quartile 1
IMD quartile 2
IMD quartile 3
IMD quartile 4

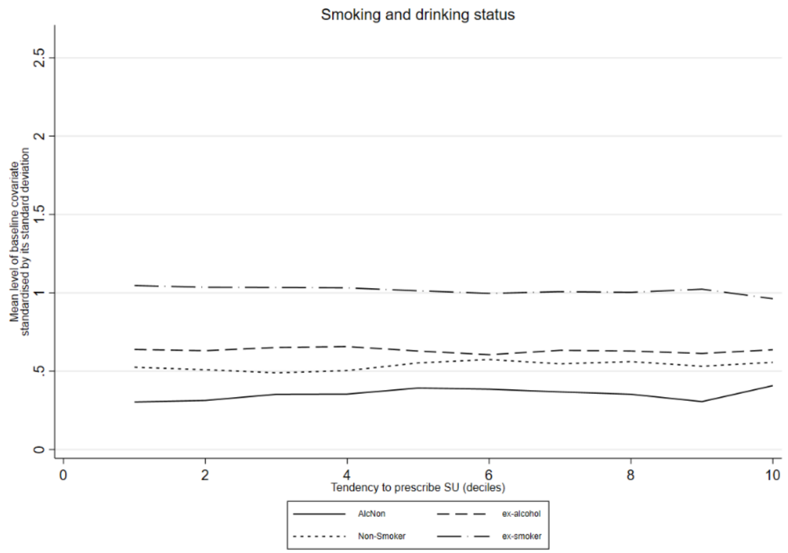
Smoking and drinking status
Mean level of baseline covariate standardised by its standard deviation
0
.5
1
1.5
2
2.5
0
2
4
6
8
10
Tendency to prescribe SU (deciles)
AlcNon
ex-alcohol
Non-Smoker
ex-smoker

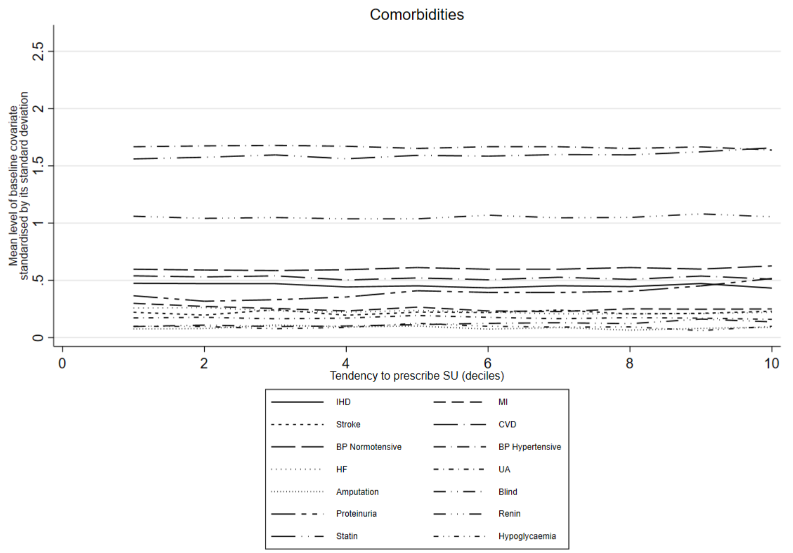
Comorbidities
Mean level of baseline covariate standardised by its standard deviation
0
.5
1
1.5
2
2.5
0
2
4
6
8
10
Tendency to prescribe SU (deciles)
IHD
MI
Stroke
CVD
BP Normotensive
BP Hypertensive
HF
UA
Amputation
Blind
Proteinuria
Renin
Statin
Hypoglycaemia

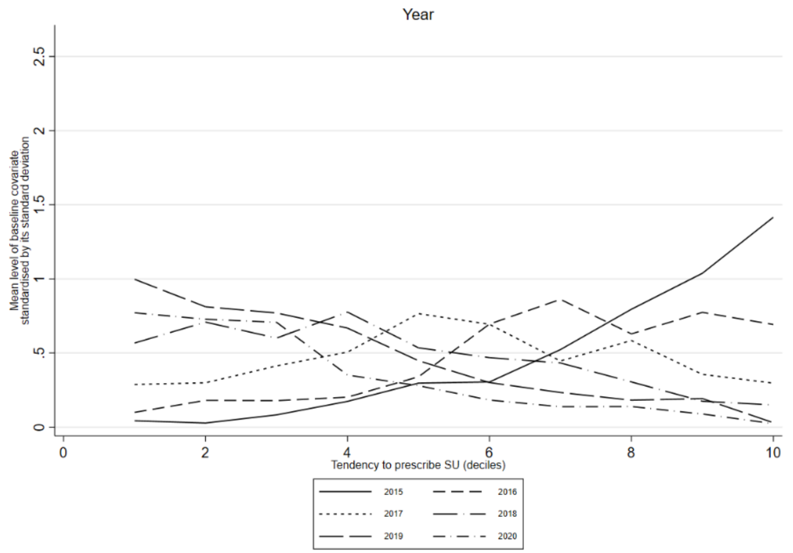
Year
Mean level of baseline covariate standardised by its standard deviation
0
.5
1
1.5
2
2.5
0
2
4
6
8
10
Tendency to prescribe SU (deciles)
2015
2016
2017
2018
2019
2020

Supplementary figure 2B: Covariate balance plots according to levels of the instrumental variable for DPP4i

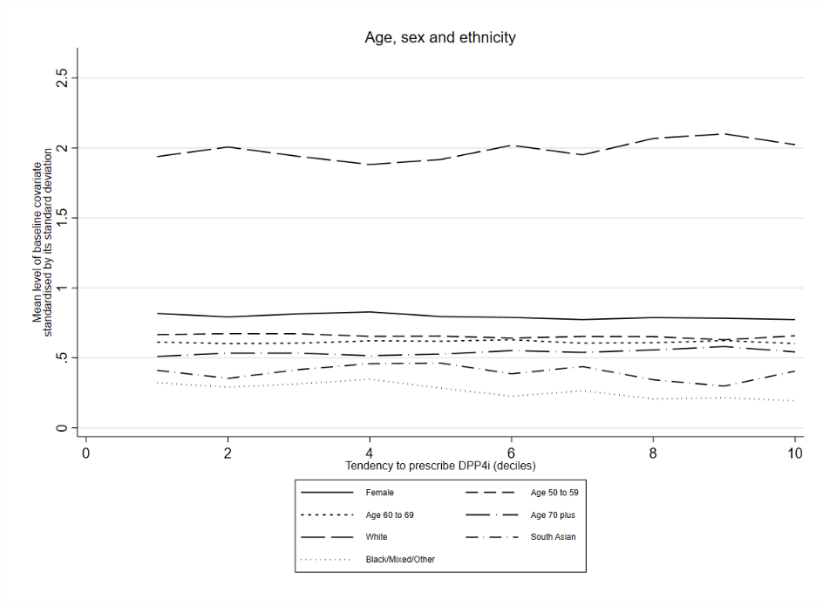

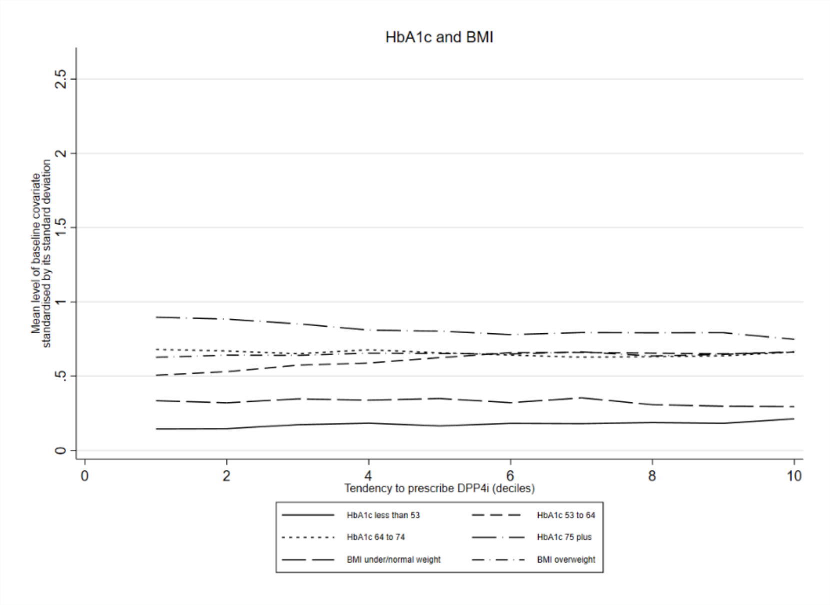

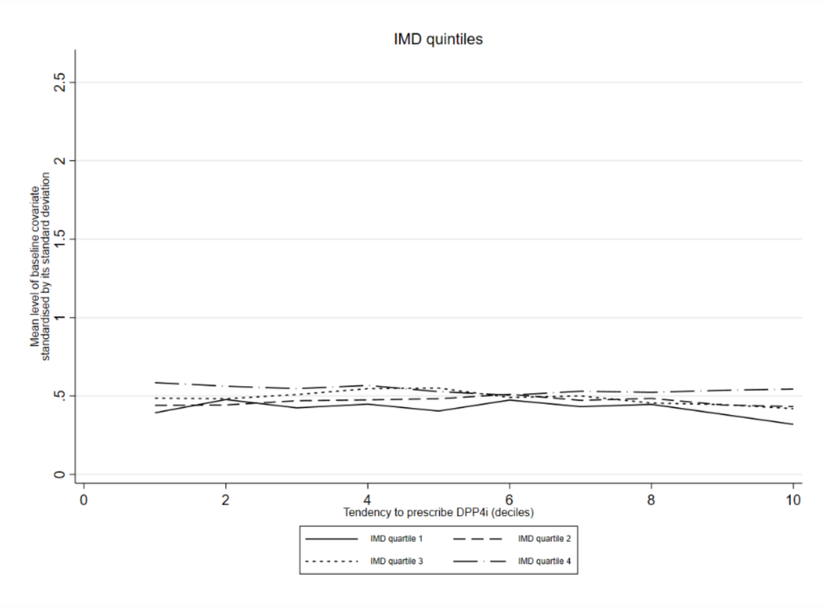
IMD quintiles
Mean level of baseline covariate standardised by its standard deviation
Tendency to prescribe DPP4i (deciles)
IMD quartile 1
IMD quartile 2
IMD quartile 3
IMD quartile 4

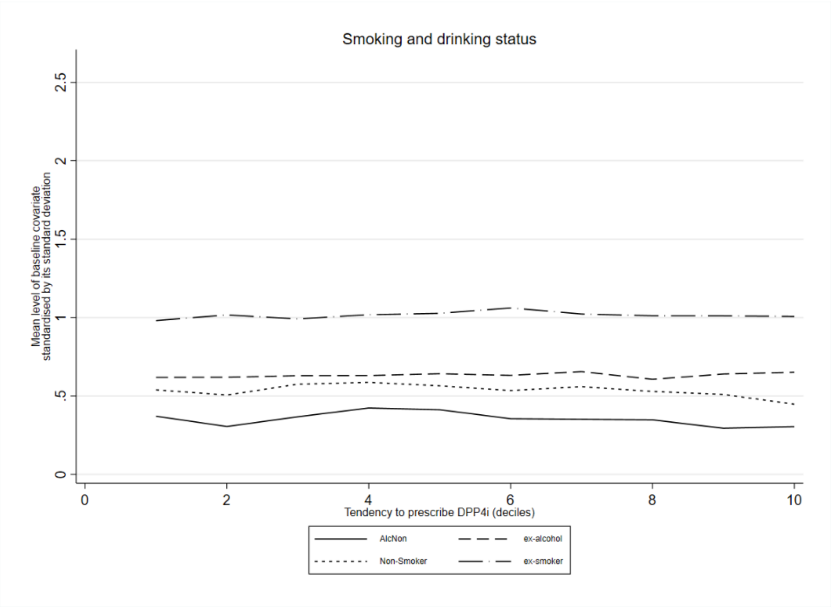
Smoking and drinking status
Mean level of baseline covariate standardised by its standard deviation
Tendency to prescribe DPP4i (deciles)
AlcNon
ex-alcohol
Non-Smoker
ex-smoker

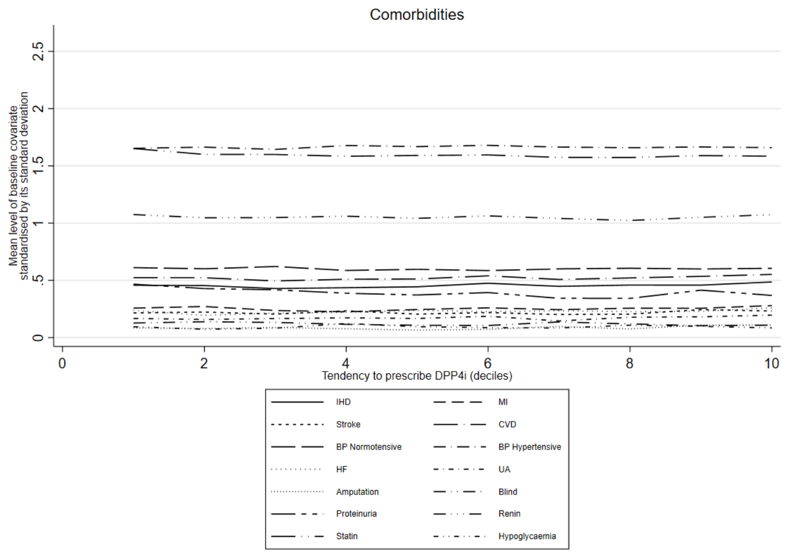

Comorbidities
Mean level of baseline covariate standardised by its standard deviation
0
.5
1
1.5
2
2.5
0
2
4
6
8
10
Tendency to prescribe DPP4i (deciles)
IHD
MI
Stroke
CVD
BP Normotensive
BP Hypertensive
HF
UA
Amputation
Blind
Proteinuria
Renin
Statin
Hypoglycaemia

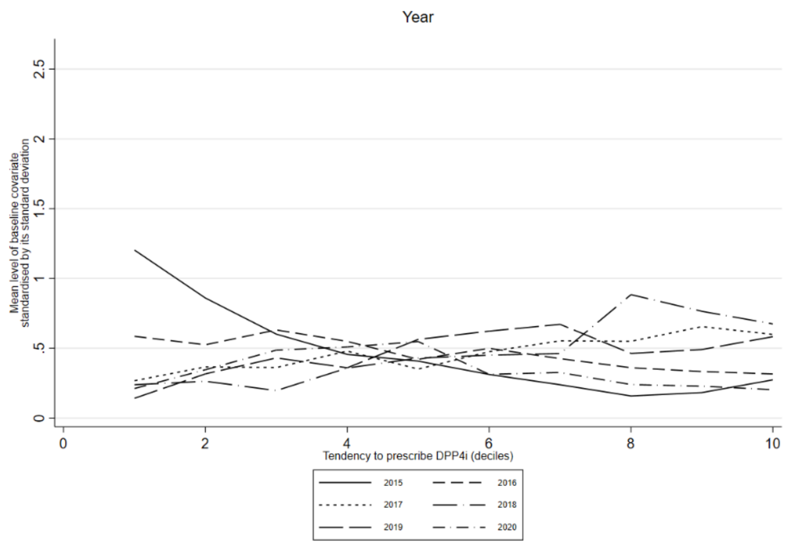

Year
Mean level of baseline covariate standardised by its standard deviation
0
.5
1
1.5
2
2.5
0
2
4
6
8
10
Tendency to prescribe DPP4i (deciles)
2015
2016
2017
2018
2019
2020

Supplementary figure 2C: Covariate balance plots according to levels of the instrumental variable for SGLT2i

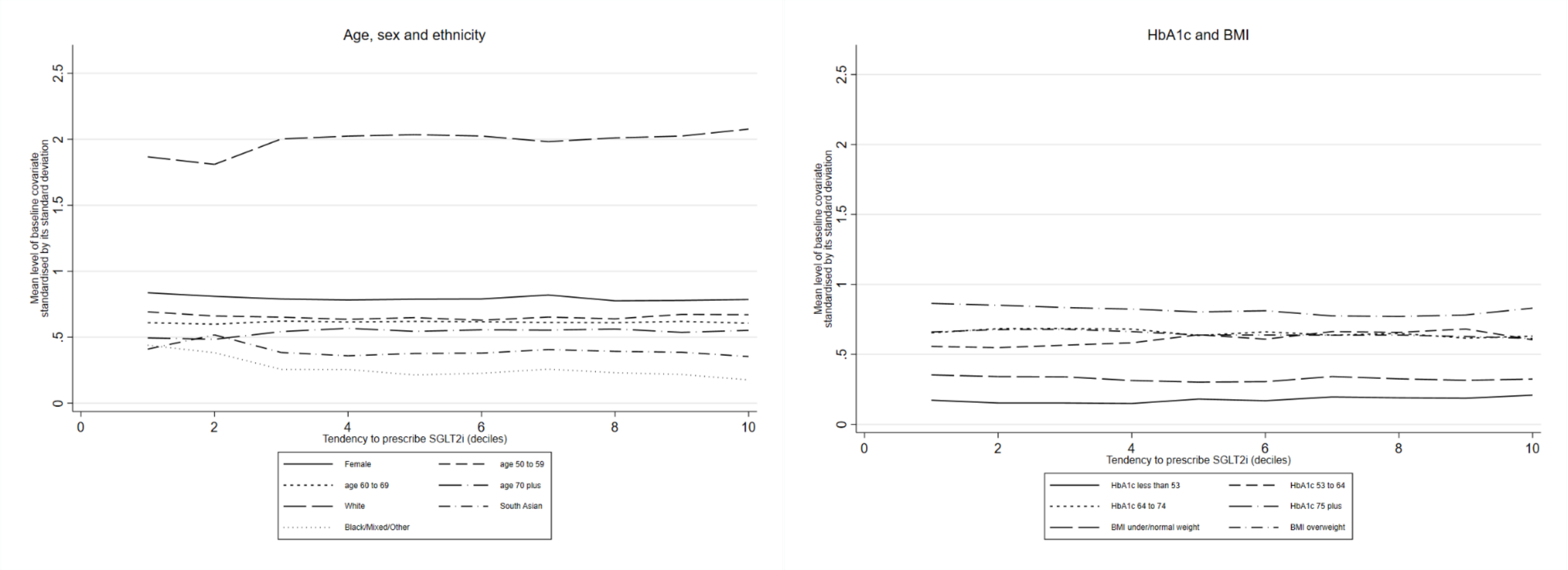

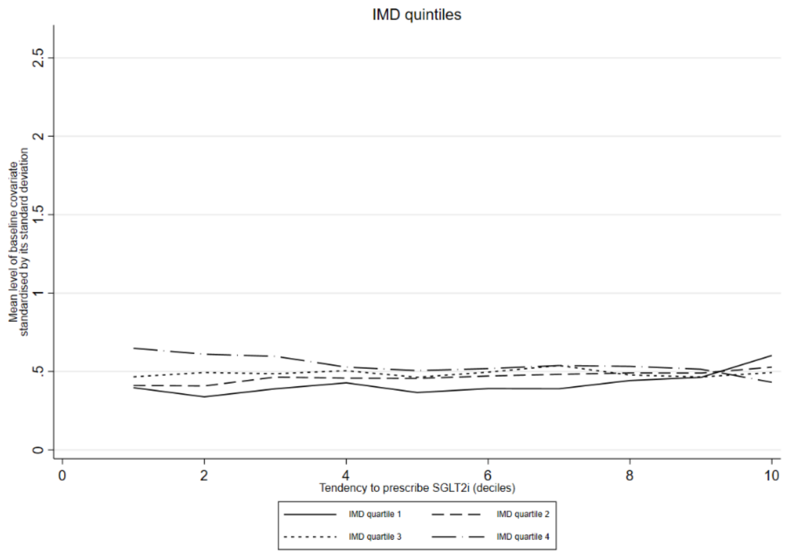
IMD quintiles
Mean level of baseline covariate standardised by its standard deviation
0
.5
1
1.5
2
2.5
0
2
4
6
8
10
Tendency to prescribe SGLT2i (deciles)
IMD quartile 1
IMD quartile 2
IMD quartile 3
IMD quartile 4

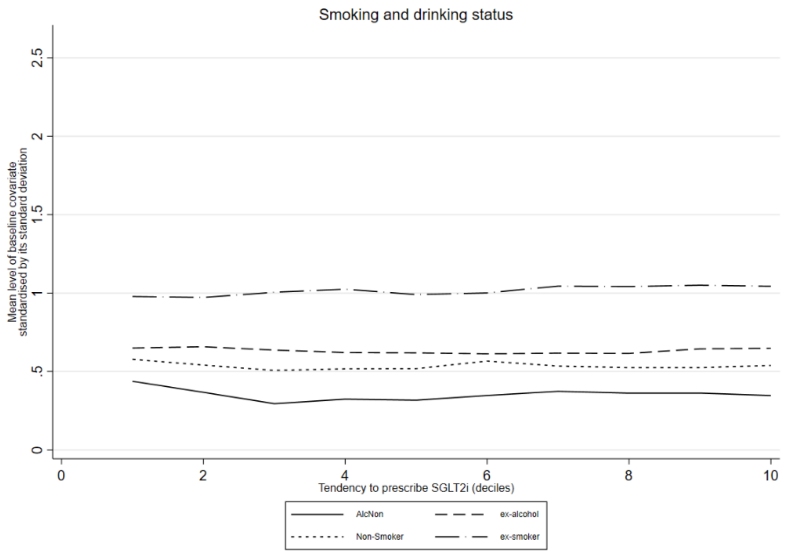
Smoking and drinking status
Mean level of baseline covariate standardised by its standard deviation
0
.5
1
1.5
2
2.5
0
2
4
6
8
10
Tendency to prescribe SGLT2i (deciles)
AlcNon
ex-alcohol
Non-Smoker
ex-smoker

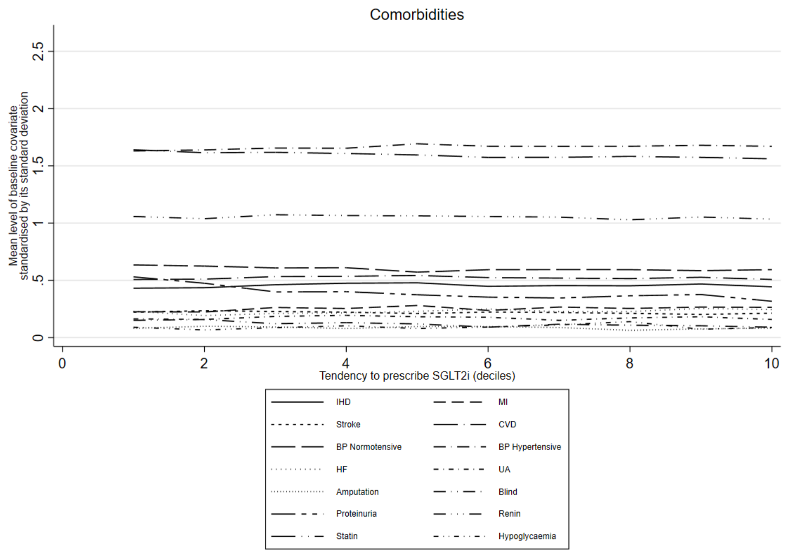
Comorbidities
Mean level of baseline covariate standardised by its standard deviation
0
.5
1
1.5
2
2.5
0
2
4
6
8
10
Tendency to prescribe SGLT2i (deciles)
IHD
MI
Stroke
CVD
BP Normotensive
BP Hypertensive
HF
UA
Amputation
Blind
Proteinuria
Renin
Statin
Hypoglycaemia

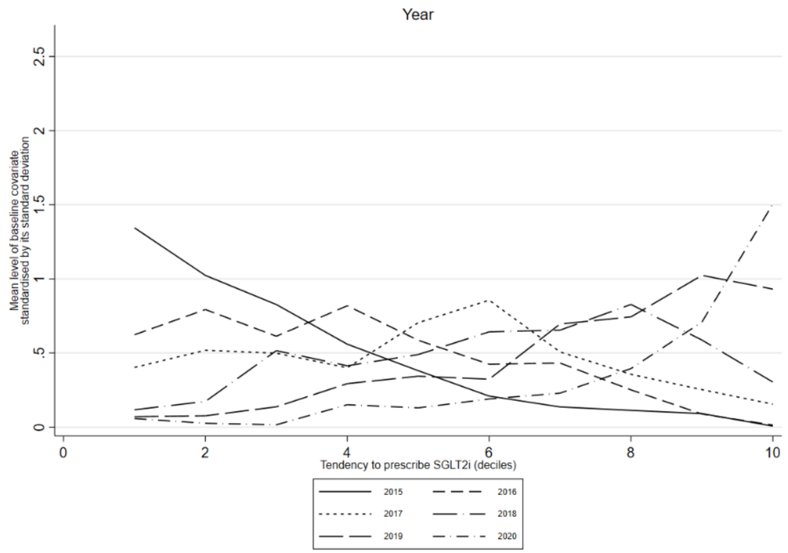
Year
Mean level of baseline covariate standardised by its standard deviation
0
.5
1
1.5
2
2.5
0
2
4
6
8
10
Tendency to prescribe SGLT2i (deciles)
2015
2016
2017
2018
2019
2020

Supplementary figure 3: Mean change in HbA1c (mmol/mol), eGFR (mL/min/1.73m^2), BMI (kg/m^2), and SBP (mm Hg) from baseline during follow-up

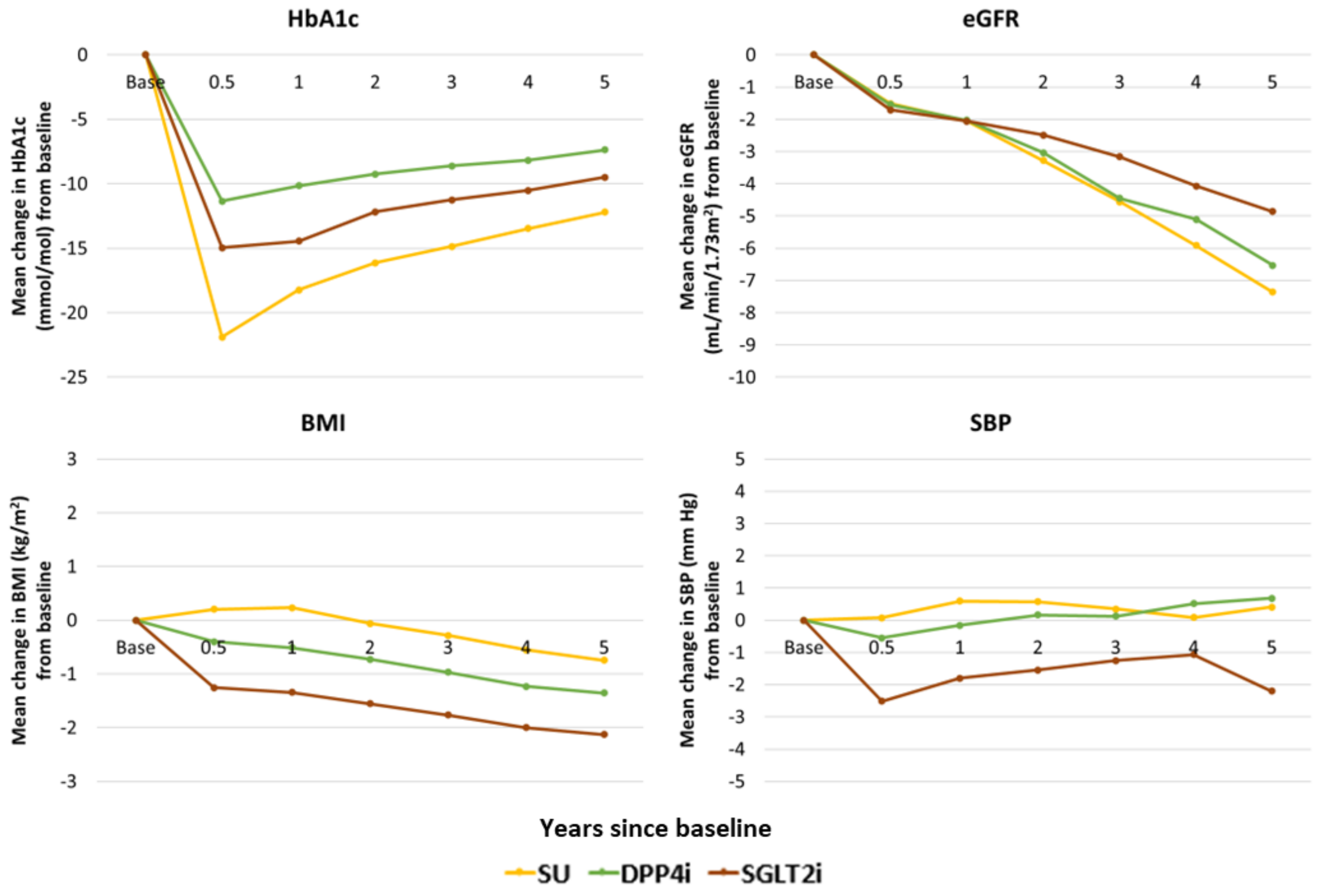

Supplementary figure 4: Cumulative failure curve for time to 40% decline in eGFR from baseline up to 2 years follow-up by exposure status

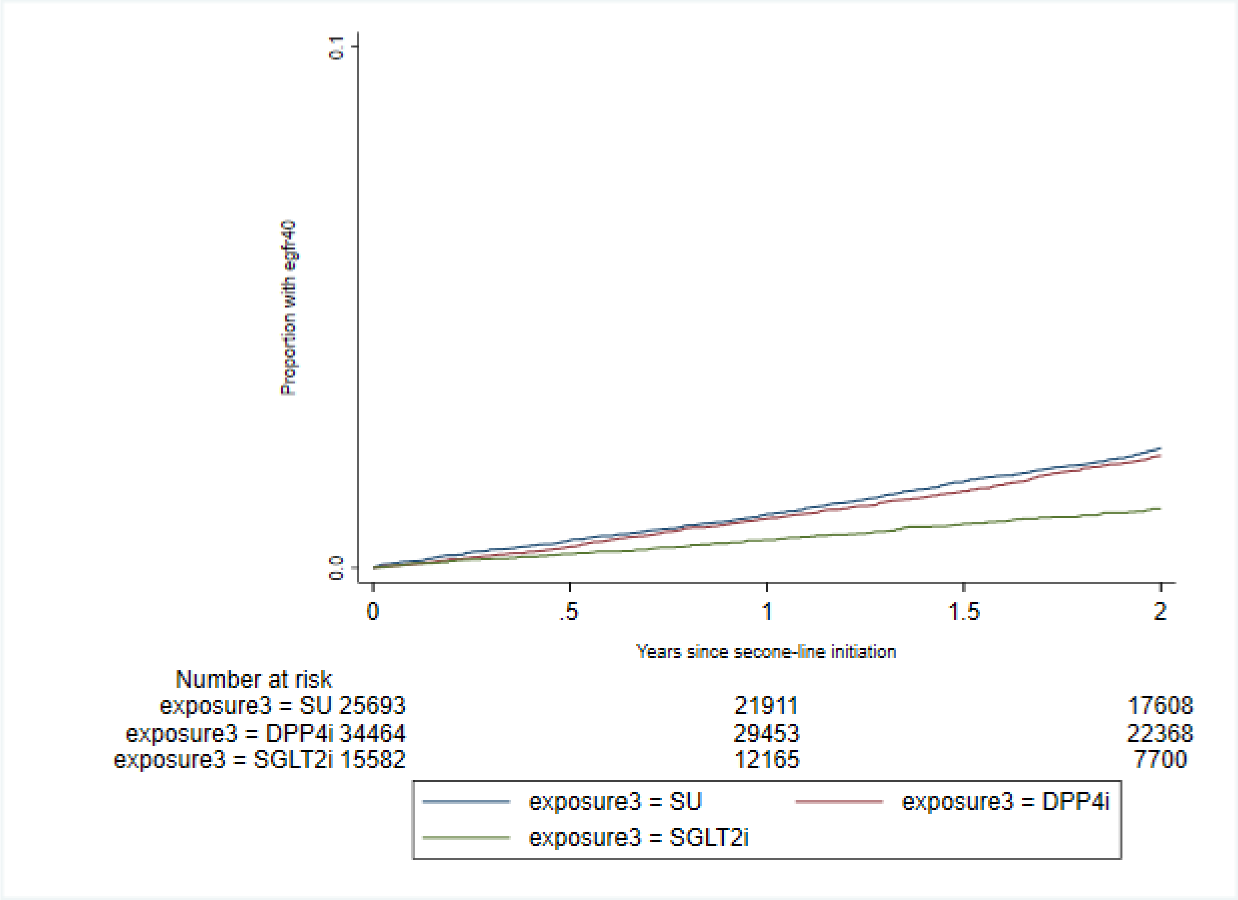

Supplementary figure 5: Cumulative failure curve for end-stage kidney disease (ESKD) up to 2-years follow-up stratified by exposure status

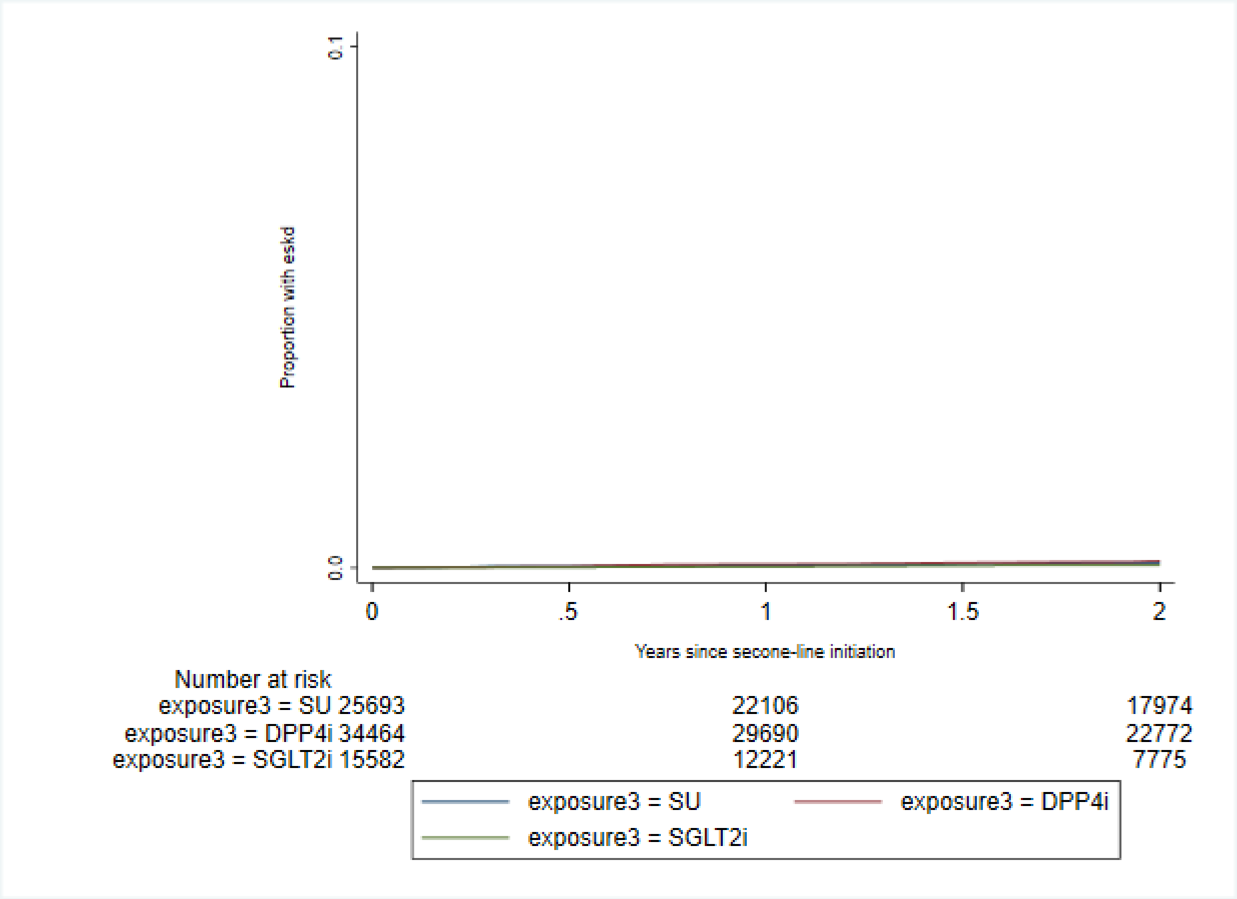

Supplementary figure 6: Cumulative failure curve for major adverse kidney event (MAKE) up to 2-years follow-up by exposure status

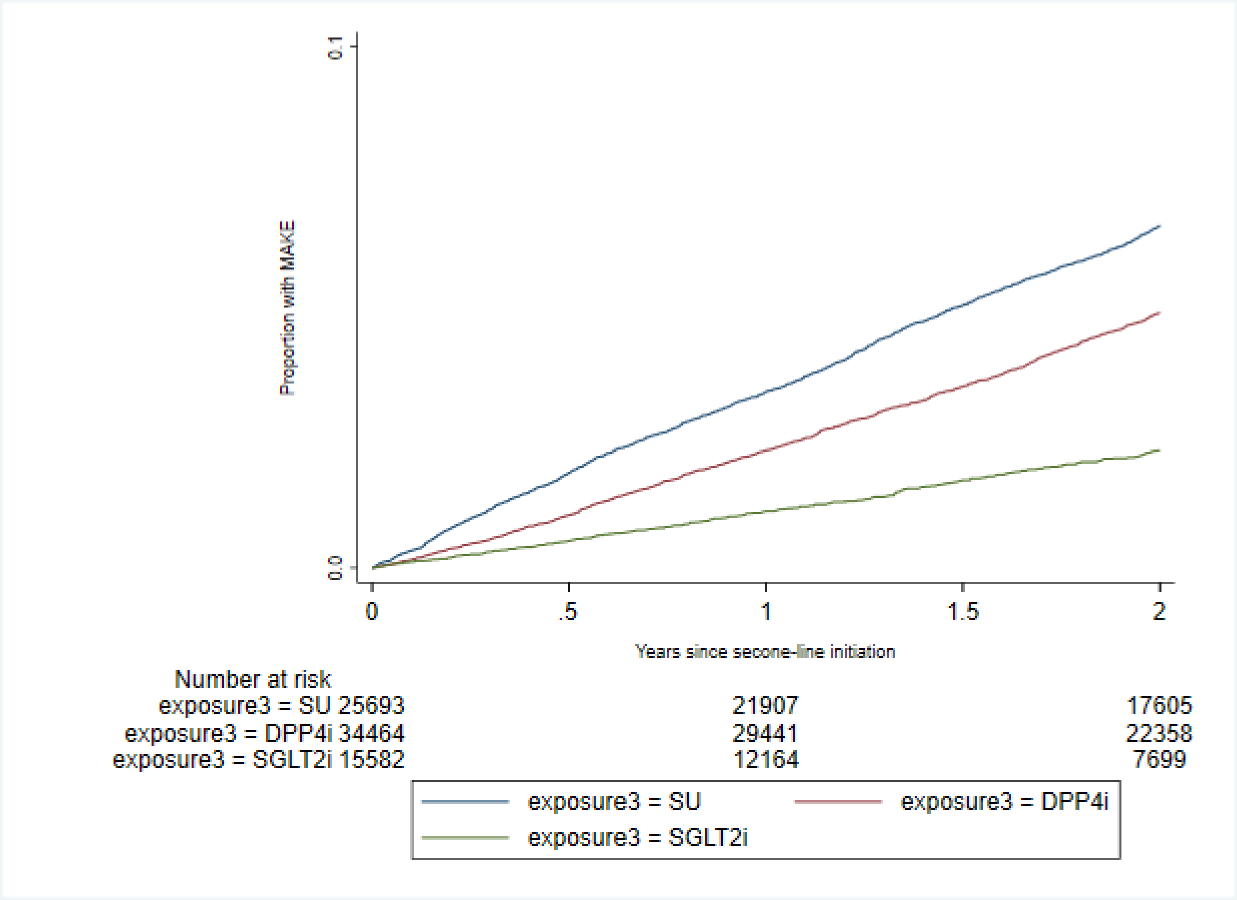

Supplementary figure 7: Cumulative failure curve for heart failure hospitalisation up to 2-years follow-up by exposure status

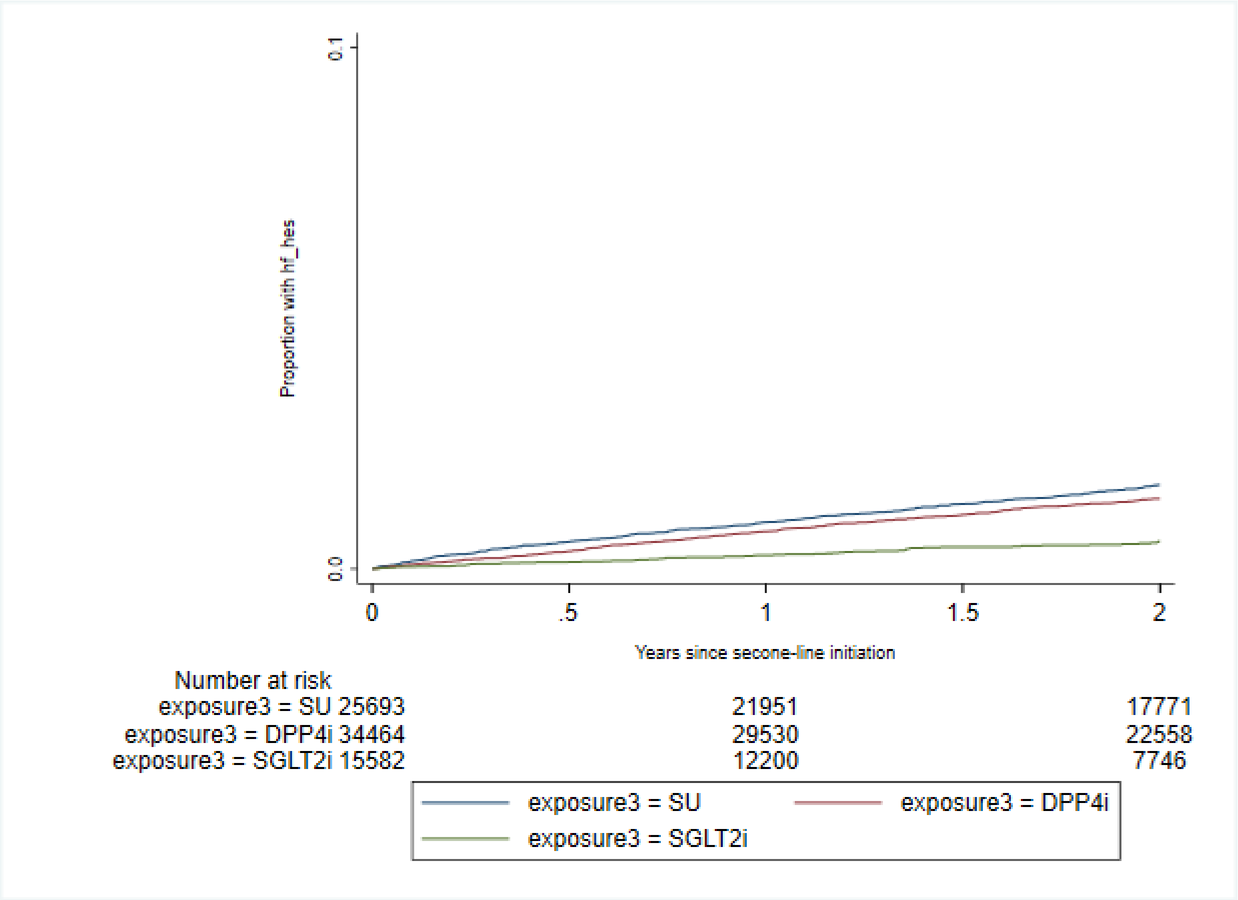

Supplementary figure 8: Cumulative failure curve for 3-point major adverse cardiovascular event (MACE) by exposure status

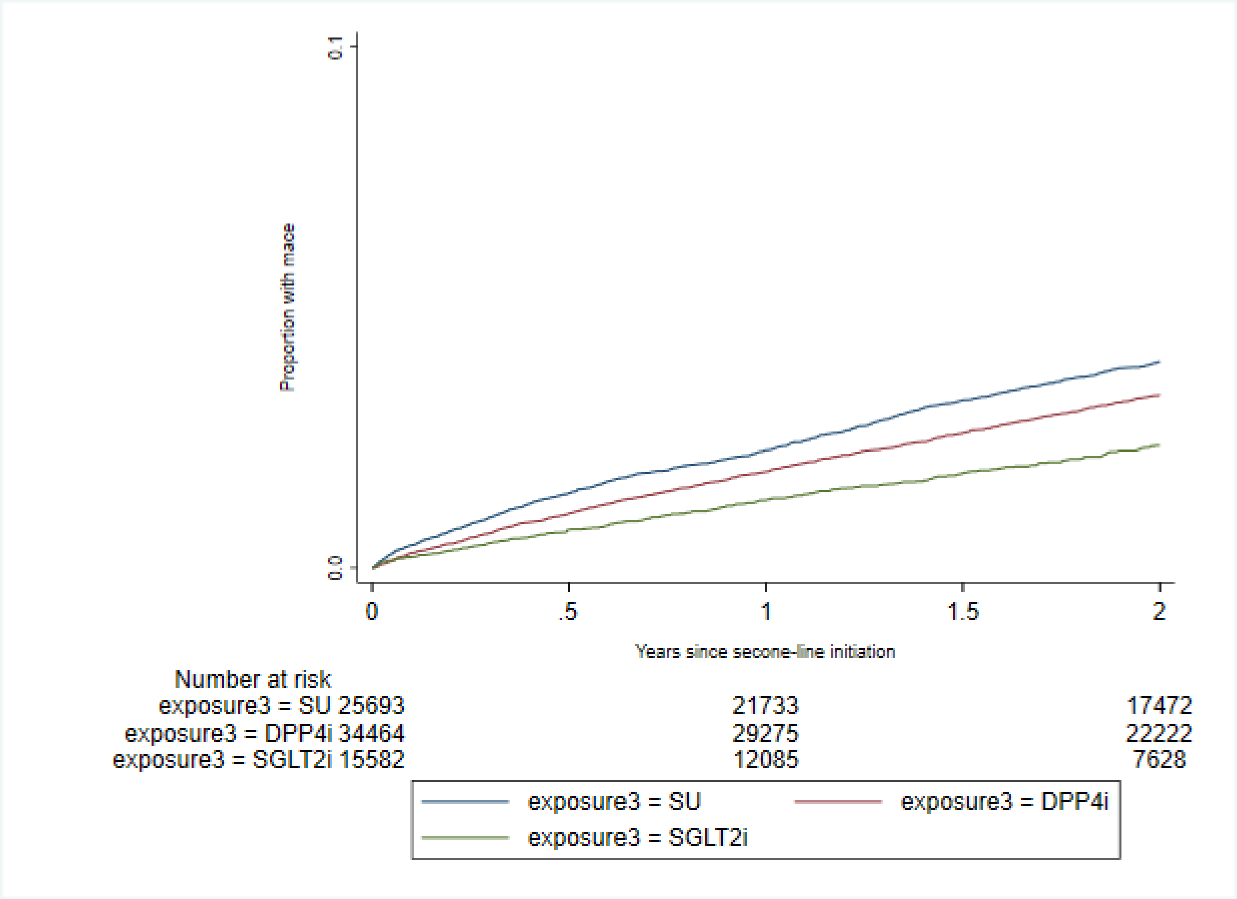

Supplementary figure 9: Cumulative failure curve for all-cause mortality by exposure status

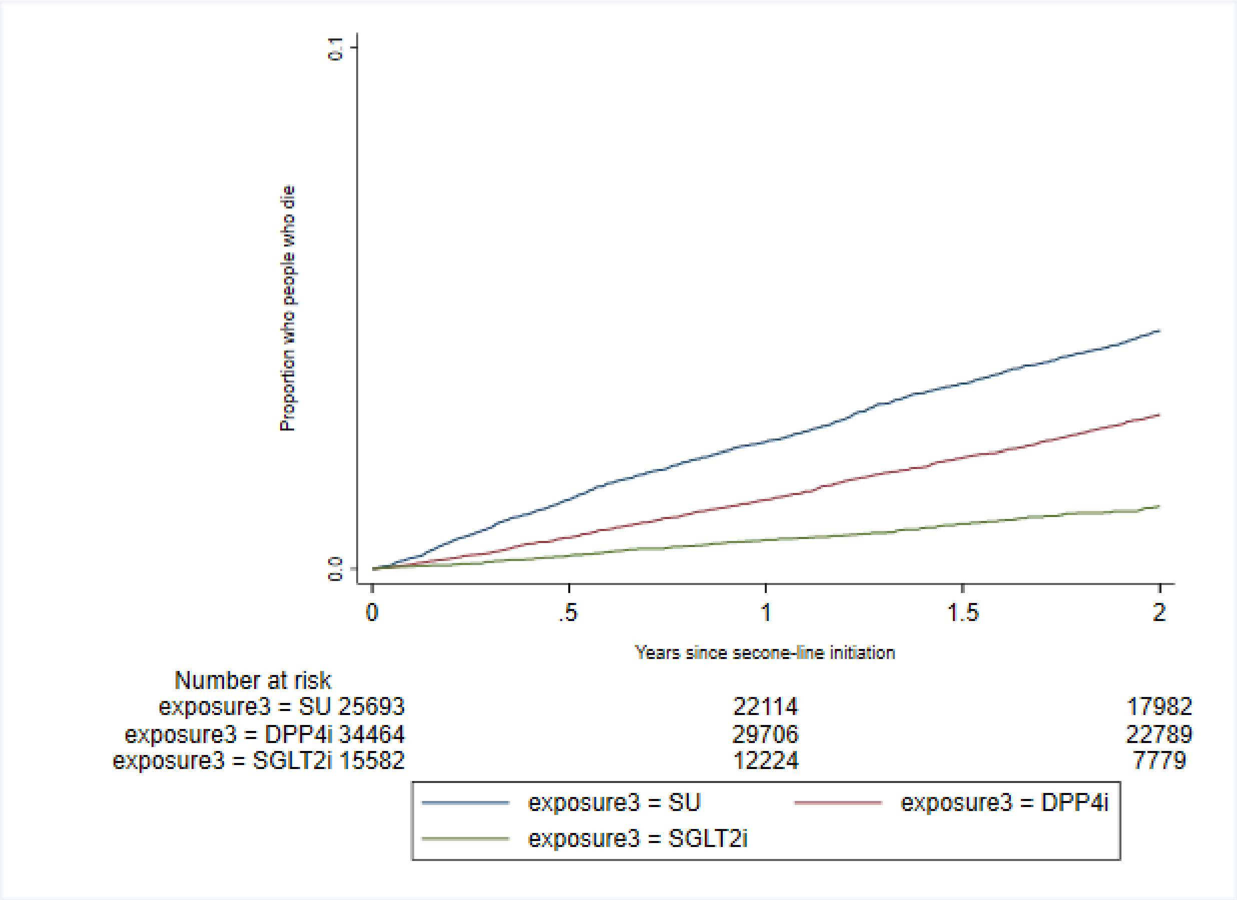

Supplementary figure 10: Propensity score distribution before adjustment for the inverse probability of treatment weighting analysis (IPTW)

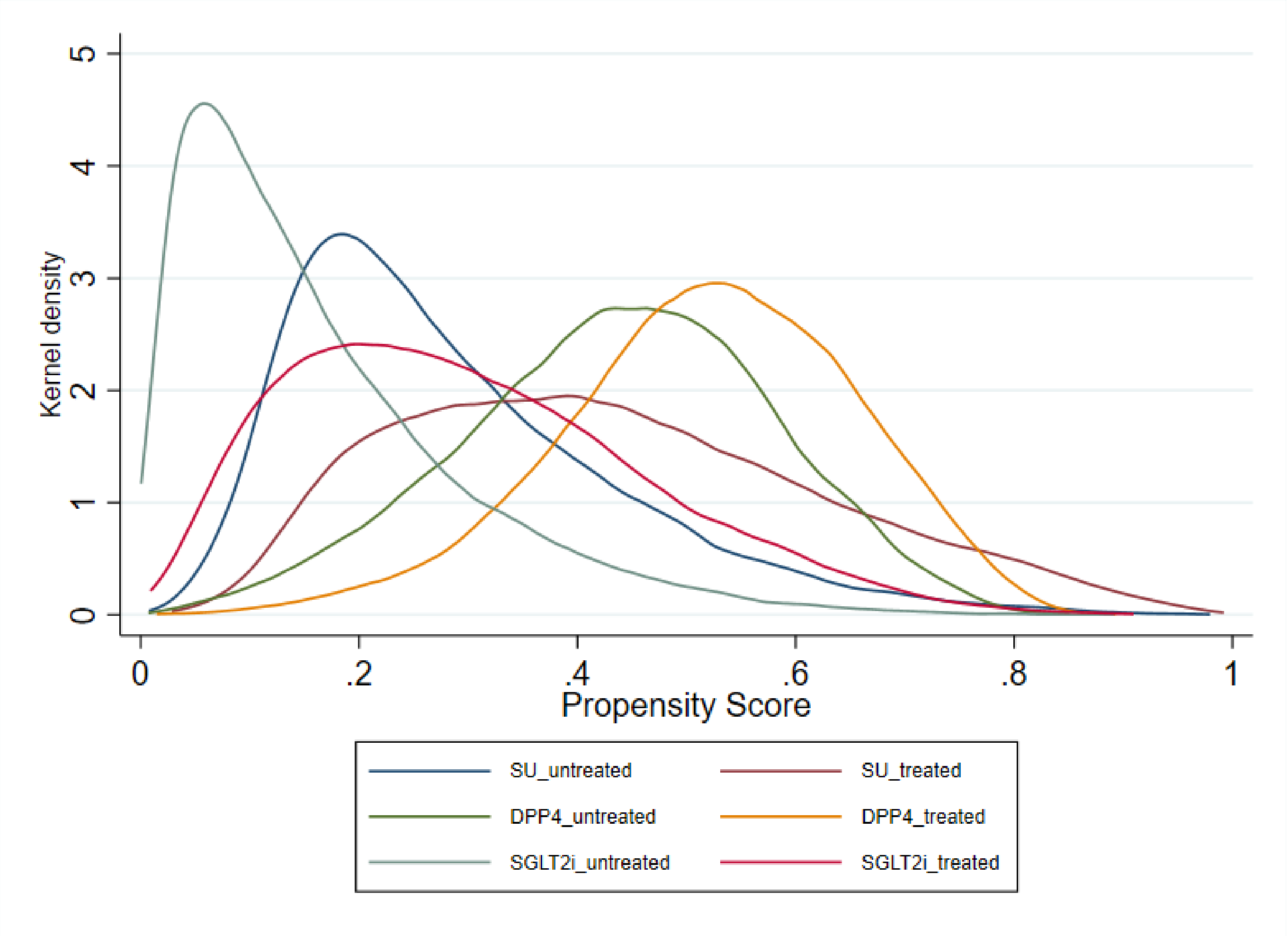

Supplementary figure 11: Propensity score distribution after adjustment for the inverse probability of treatment analysis (IPTW)

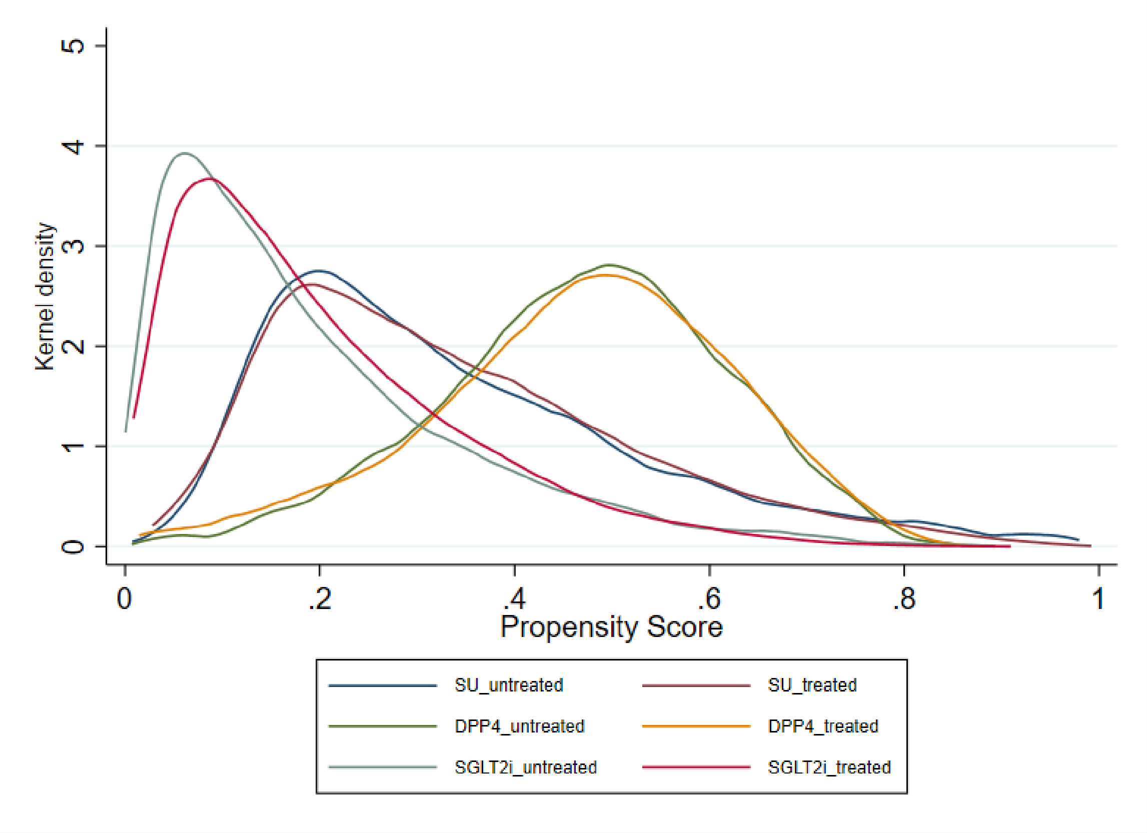

REFERENCES

1. Gourieroux C, Monfort A, Renault E, Trognon A. Generalised residuals. *Journal of Econometrics*. 1987/01/01/ 1987;34(1):5-32. doi:https://doi.org/10.1016/0304-4076(87)90065-0
2. Martínez-Camblor P, Mackenzie T, Staiger DO, Goodney PP, O'Malley AJ. Adjusting for bias introduced by instrumental variable estimation in the Cox proportional hazards model. *Biostatistics*. Jan 1 2019;20(1):80-96. doi:10.1093/biostatistics/kxx062
3. Martínez-Camblor P, Mackenzie T, Staiger DO, Goodney PP, O'Malley AJ. Adjusting for bias introduced by instrumental variable estimation in the Cox proportional hazards model. *Biostatistics*. 2017;20(1):80-96. doi:10.1093/biostatistics/kxx062
4. Frank lE, Friedman JH. A Statistical View of Some Chemometrics Regression Tools. *Technometrics*. 1993/05/01 1993;35(2):109-135. doi:10.1080/00401706.1993.10485033
5. Tibshirani R. Regression Shrinkage and Selection Via the Lasso. *Journal of the Royal Statistical Society: Series B (Methodological)*. 1996;58(1):267-288. doi:https://doi.org/10.1111/j.2517-6161.1996.tb02080.x
6. Belloni A, Chen D, Chernozhukov V, Hansen C. Sparse Models and Methods for Optimal Instruments With an Application to Eminent Domain. *Econometrica*. 2012;80(6):2369-2429. doi:https://doi.org/10.3982/ECTA9626
7. Ahrens A, Hansen CB, Schaffer ME. lassopack: Model selection and prediction with regularized regression in Stata. *The Stata Journal*. 2020;20(1):176-235. doi:10.1177/1536867x20909697
8. van Buuren S, Oudshoorn, C.G.M. *Multivariate Imputation by Chained Equations: MICE V1.0 User's manual. TNO Report PG/VGZ/00.038*. 2000. http://www.multiple-imputation.com/
9. Powney M, Williamson P, Kirkham J, Kolamunnage-Dona R. A review of the handling of missing longitudinal outcome data in clinical trials. *Trials*. Jun 19 2014;15:237. doi:10.1186/1745-6215-15-237
10. Lee KJ, Roberts G, Doyle LW, Anderson PJ, Carlin JB. Multiple imputation for missing data in a longitudinal cohort study: a tutorial based on a detailed case study involving imputation of missing outcome data. *International Journal of Social Research Methodology*. 2016/09/02 2016;19(5):575-591. doi:10.1080/13645579.2015.1126486
11. Morris TP, White IR, Royston P. Tuning multiple imputation by predictive mean matching and local residual draws. *BMC Medical Research Methodology*. 2014/06/05 2014;14(1):75. doi:10.1186/1471-2288-14-75
12. Williamson EJ, Walker AJ, Bhaskaran K, et al. Factors associated with COVID-19-related death using OpenSAFELY. *Nature*. 2020/08/01 2020;584(7821):430-436. doi:10.1038/s41586-020-2521-4

13. Mathur R, Rentsch CT, Morton CE, et al. Ethnic differences in SARS-CoV-2 infection and COVID-19-related hospitalisation, intensive care unit admission, and death in 17 million adults in England: an observational cohort study using the OpenSAFELY platform. *Lancet (London, England)*. May 8 2021;397(10286):1711-1724. doi:10.1016/s0140-6736(21)00634-6
14. Meng X-L. Multiple-Imputation Inferences with Uncongenial Sources of Input. *Statistical Science*. 1994;9(4):538-558, 21.
15. Seaman SR, Bartlett JW, White IR. Multiple imputation of missing covariates with non-linear effects and interactions: an evaluation of statistical methods. *BMC Medical Research Methodology*. 2012/04/10 2012;12(1):46. doi:10.1186/1471-2288-12-46
16. van Buuren S, Groothuis-Oudshoorn K. mice: Multivariate Imputation by Chained Equations in R. *Journal of Statistical Software*. 12/12 2011;45(3):1 - 67. doi:10.18637/jss.v045.i03
17. White IR, Royston P, Wood AM. Multiple imputation using chained equations: Issues and guidance for practice. *Stat Med*. Feb 20 2011;30(4):377-99. doi:10.1002/sim.4067
18. van Buuren S. *Flexible Imputation of Missing Data, Second Edition*. 2 ed. Chapman and Hall/CRC; 2018:444.
19. Carpenter JR, Kenward MG. *Multiple Imputation and its Application*. John Wiley & Sons, Ltd; 2012.
20. Bartlett JW, Hughes RA. Bootstrap inference for multiple imputation under uncongeniality and misspecification. *Statistical Methods in Medical Research*. 2020;29(12):3533-3546. doi:10.1177/0962280220932189
21. Schomaker M, Heumann C. Bootstrap inference when using multiple imputation. *Stat Med*. Jun 30 2018;37(14):2252-2266. doi:10.1002/sim.7654
22. Wilkinson S, Douglas IJ, Williamson E, et al. Factors associated with choice of intensification treatment for type 2 diabetes after metformin monotherapy: a cohort study in UK primary care. *Clin Epidemiol*. 2018;10:1639-1648. doi:10.2147/CLEP.S176142
23. Wilkinson S, Williamson E, Pokrajac A, et al. Comparative effects of sulphonylureas, dipeptidyl peptidase-4 inhibitors and sodium-glucose co-transporter-2 inhibitors added to metformin monotherapy: a propensity-score matched cohort study in UK primary care. *Diabetes, Obesity and Metabolism*. 2020;22(5):847-856. doi:https://doi.org/10.1111/dom.13970
24. NICE guideline [NG28]: Type 2 diabetes in adults: management. Web. NICE. Accessed 3 March, 2022. https://www.nice.org.uk/guidance/ng28/chapter/Recommendations#reviewing-drug-treatments
25. Bhaskaran K, Dos-Santos-Silva I, Leon DA, Douglas IJ, Smeeth L. Association of BMI with overall and cause-specific mortality: a population-based cohort study of 3·6 million adults in the UK. *Lancet Diabetes Endocrinol*. Dec 2018;6(12):944-953. doi:10.1016/s2213-8587(18)30288-2
26. Bhaskaran K, Forbes HJ, Douglas I, Leon DA, Smeeth L. Representativeness and optimal use of body mass index (BMI) in the UK Clinical Practice Research Datalink (CPRD). *BMJ Open*. 2013;3(9):e003389. doi:10.1136/bmjopen-2013-003389

27. Levey AS, Inker LA, Matsushita K, et al. GFR decline as an end point for clinical trials in CKD: a scientific workshop sponsored by the National Kidney Foundation and the US Food and Drug Administration. *American journal of kidney diseases : the official journal of the National Kidney Foundation*. Dec 2014;64(6):821-35. doi:10.1053/j.ajkd.2014.07.030
28. Stürmer T, Webster-Clark M, Lund JL, et al. Propensity Score Weighting and Trimming Strategies for Reducing Variance and Bias of Treatment Effect Estimates: A Simulation Study. *Am J Epidemiol*. Aug 1 2021;190(8):1659-1670. doi:10.1093/aje/kwab041
29. Stürmer T, Rothman KJ, Avorn J, Glynn RJ. Treatment effects in the presence of unmeasured confounding: dealing with observations in the tails of the propensity score distribution--a simulation study. *Am J Epidemiol*. Oct 1 2010;172(7):843-54. doi:10.1093/aje/kwq198
30. Bidulka P, O'Neill S, Basu A, et al. Protocol for an observational cohort study investigating personalised medicine for intensification of treatment in people with type 2 diabetes mellitus: the PERMIT study. *BMJ Open*. 2021;11(9):e046912. doi:10.1136/bmjopen-2020-046912
31. Zinman B, Wanner C, Lachin JM, et al. Empagliflozin, Cardiovascular Outcomes, and Mortality in Type 2 Diabetes. *New England Journal of Medicine*. 2015/11/26 2015;373(22):2117-2128. doi:10.1056/NEJMoa1504720
32. Neal B, Perkovic V, Mahaffey KW, et al. Canagliflozin and Cardiovascular and Renal Events in Type 2 Diabetes. *New England Journal of Medicine*. 2017/08/17 2017;377(7):644-657. doi:10.1056/NEJMoa1611925
33. Wiviott SD, Raz I, Bonaca MP, et al. Dapagliflozin and Cardiovascular Outcomes in Type 2 Diabetes. *New England Journal of Medicine*. 2019/01/24 2018;380(4):347-357. doi:10.1056/NEJMoa1812389
34. Rosenstock J, Kahn SE, Johansen OE, et al. Effect of Linagliptin vs Glimepiride on Major Adverse Cardiovascular Outcomes in Patients With Type 2 Diabetes: The CAROLINA Randomized Clinical Trial. *Jama*. Sep 19 2019;322(12):1155-66. doi:10.1001/jama.2019.13772
35. Cannon CP, Pratley R, Dagogo-Jack S, et al. Cardiovascular Outcomes with Ertugliflozin in Type 2 Diabetes. *New England Journal of Medicine*. 2020;383(15):1425-1435. doi:10.1056/NEJMoa2004967
36. Perkovic V, Jardine MJ, Neal B, et al. Canagliflozin and Renal Outcomes in Type 2 Diabetes and Nephropathy. *New England Journal of Medicine*. 2019/06/13 2019;380(24):2295-2306. doi:10.1056/NEJMoa1811744
37. Empagliflozin in Patients with Chronic Kidney Disease. *New England Journal of Medicine*. 2022;388(2):117-127. doi:10.1056/NEJMoa2204233
38. Glycemia Reduction in Type 2 Diabetes — Glycemic Outcomes. *New England Journal of Medicine*. 2022/09/22 2022;387(12):1063-1074. doi:10.1056/NEJMoa2200433
39. Glycemia Reduction in Type 2 Diabetes — Microvascular and Cardiovascular Outcomes. *New England Journal of Medicine*. 2022/09/22 2022;387(12):1075-1088. doi:10.1056/NEJMoa2200436
40. McMurray JJV, Solomon SD, Inzucchi SE, et al. Dapagliflozin in Patients with Heart Failure and Reduced Ejection Fraction. *New England Journal of Medicine*. 2019/11/21 2019;381(21):1995-2008. doi:10.1056/NEJMoa1911303

41. Heerspink HJL, Stefánsson BV, Correa-Rotter R, et al. Dapagliflozin in Patients with Chronic Kidney Disease. *New England Journal of Medicine*. 2020;383(15):1436-1446. doi:10.1056/NEJMoa2024816
42. Packer M, Anker SD, Butler J, et al. Cardiovascular and Renal Outcomes with Empagliflozin in Heart Failure. *New England Journal of Medicine*. 2020;383(15):1413-1424. doi:10.1056/NEJMoa2022190
